# Mobile health solutions for atrial fibrillation detection and management: a systematic review

**DOI:** 10.1007/s00392-021-01941-9

**Published:** 2021-09-21

**Authors:** Astrid N. L. Hermans, Monika Gawalko, Lisa Dohmen, Rachel M. J. van der Velden, Konstanze Betz, David Duncker, Dominique V. M. Verhaert, Hein Heidbuchel, Emma Svennberg, Lis Neubeck, Jens Eckstein, Deirdre A. Lane, Gregory Y. H. Lip, Harry J. G. M. Crijns, Prashanthan Sanders, Jeroen M. Hendriks, Nikki A. H. A. Pluymaekers, Dominik Linz

**Affiliations:** 1 Department of Cardiology, Maastricht University Medical Center and Cardiovascular Research Institute Maastricht, Maastricht, The Netherlands; 2 Institute of Pharmacology, West German Heart and Vascular Centre, University Duisburg-Essen, Duisburg, Germany; 3 1st Department of Cardiology, Medical University of Warsaw, Warsaw, Poland; 4 Department of Cardiology and Angiology, Hannover Heart Rhythm Center and Hannover Medical School, Hannover, Germany; 5 Department of Cardiology, Radboud Institute for Health Sciences and Radboud University Medical Center, Nijmegen, The Netherlands; 6 Cardiology Department, Antwerp University Hospital and Antwerp University, Antwerp, Belgium; 7 Faculty of Medicine and Life Sciences, Hasselt University, Hasselt, Belgium; 8 Department of Medicine, Karolinska Institutet, Karolinska University Hospital Huddinge, Stockholm, Sweden; 9 School of Health and Social Care, Edinburgh Napier University, Edinburgh, UK; 10 Department of Internal Medicine, University Hospital Basel, Basel, Switzerland; 11 Liverpool Centre for Cardiovascular Science, Liverpool Heart and Chest Hospital and University of Liverpool, Liverpool, UK; 12 Centre for Heart Rhythm Disorders, Royal Adelaide Hospital and University of Adelaide, Adelaide, Australia; 13 Caring Futures Institute, College of Nursing and Health Sciences, Flinders University, Adelaide, Australia; 14 Department of Biomedical Sciences, Faculty of Health and Medical Sciences, University of Copenhagen, Copenhagen, Denmark

**Keywords:** Atrial fibrillation, mHealth, Systematic review

## Abstract

**Aim:**

We aimed to systematically review the available literature on mobile Health (mHealth) solutions, including handheld and wearable devices, implantable loop recorders (ILRs), as well as mobile platforms and support systems in atrial fibrillation (AF) detection and management.

**Methods:**

This systematic review was conducted in accordance with the Preferred Reporting Items for Systematic Reviews and Meta-analyses (PRISMA) guidelines. The electronic databases PubMed (NCBI), Embase (Ovid), and Cochrane were searched for articles published until 10 February 2021, inclusive. Given that the included studies varied widely in their design, interventions, comparators, and outcomes, no synthesis was undertaken, and we undertook a narrative review.

**Results:**

We found 208 studies, which were deemed potentially relevant. Of these studies included, 82, 46, and 49 studies aimed at validating handheld devices, wearables, and ILRs for AF detection and/or management, respectively, while 34 studies assessed mobile platforms/support systems. The diagnostic accuracy of mHealth solutions differs with respect to the type (handheld devices vs wearables vs ILRs) and technology used (electrocardiography vs photoplethysmography), as well as application setting (intermittent vs continuous, spot vs longitudinal assessment), and study population.

**Conclusion:**

While the use of mHealth solutions in the detection and management of AF is becoming increasingly popular, its clinical implications merit further investigation and several barriers to widespread mHealth adaption in healthcare systems need to be overcome.

**Graphic abstract:**

Mobile health solutions for atrial fibrillation detection and management: a systematic review.

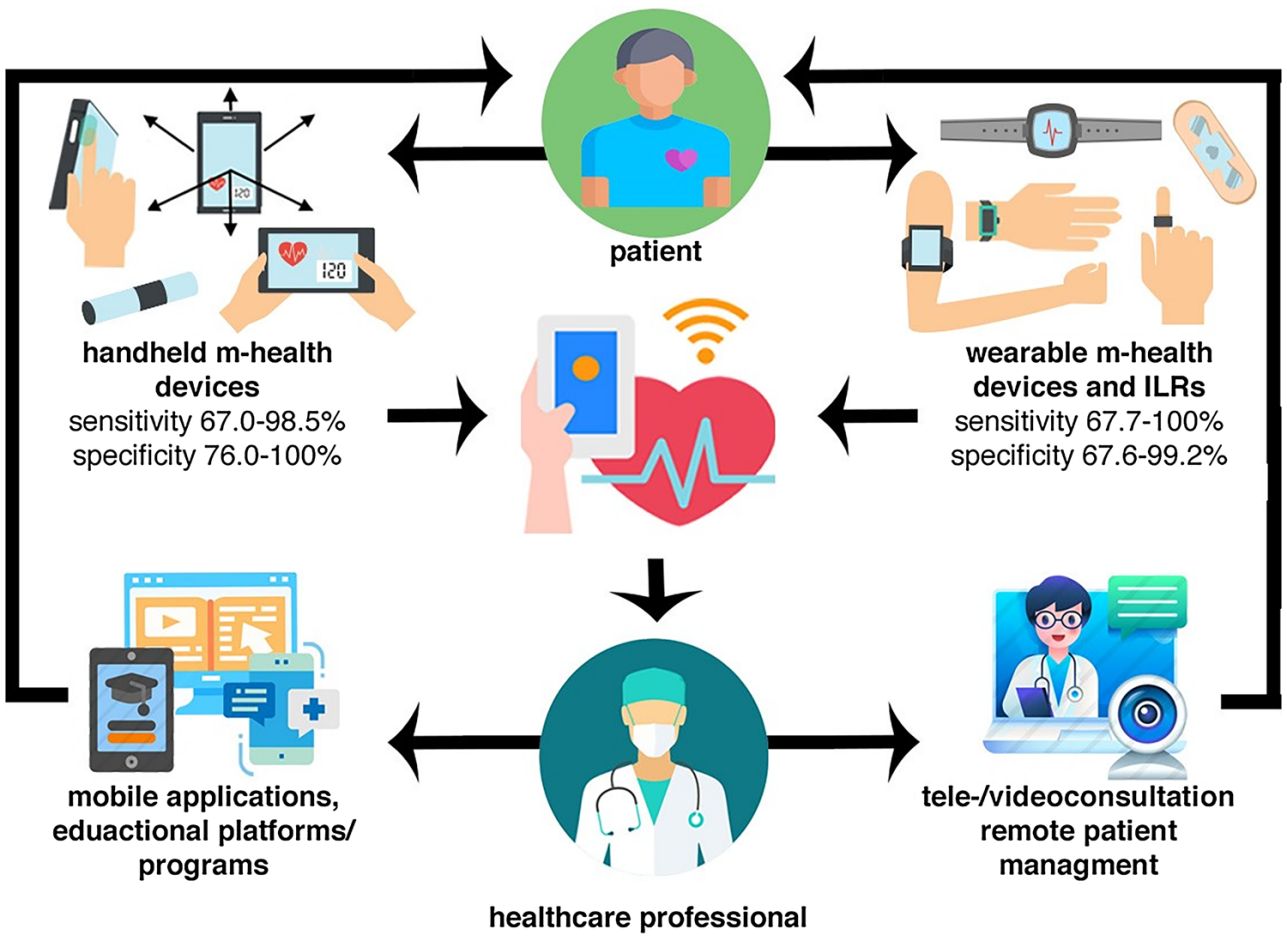

**Supplementary Information:**

The online version contains supplementary material available at 10.1007/s00392-021-01941-9.

## Introduction

**Fig. 1:**
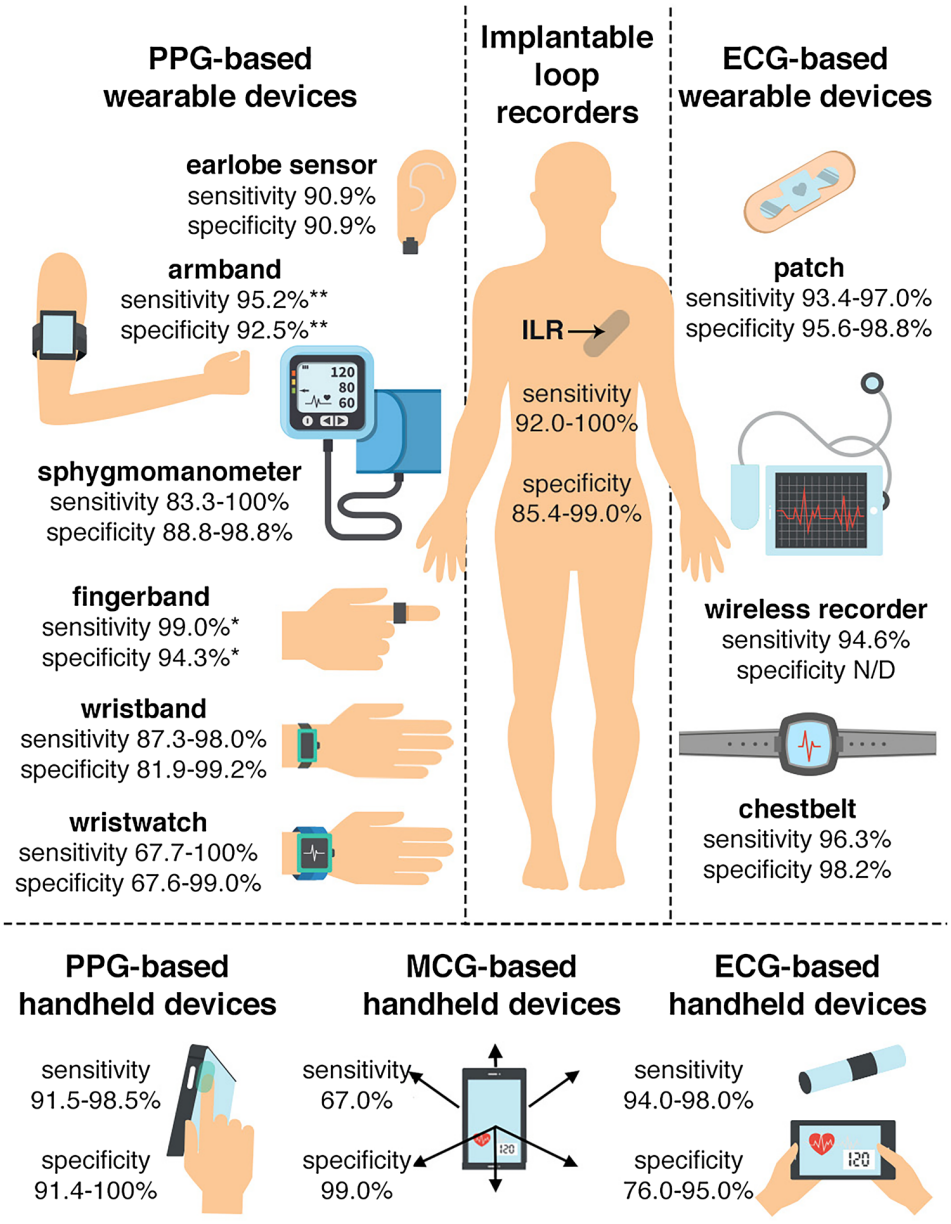
Presentation of mobile health devices and their sensitivity and specificity considering 12-lead electrocardiogram as the gold standard. This figure summarizes the literature as performed in this systematic review. Sensitivity and specificity range were given when an mHealth solution was clinically validated by > 1 study. *Single-lead electrocardiogram as the gold standard; **24-h Holter monitoring as the gold standard. *ECG* electrocardiography, *MCG* mechanocardiography, *PPG* photoplethysmography

Atrial fibrillation (AF) is the most prevalent sustained cardiac arrhythmia affecting more than 37 million people worldwide [1, 2]. According to current international guidelines, AF management should be organized in an integrated care model [3]. One important component of an integrated care model is usage of technology, such as mobile health (mHealth). mHealth is defined as “medical and public health practice supported by mobile devices, such as mobile phones, patient monitoring devices, personal digital assistants (PDAs), and other wireless devices” [4] which can engage patients in their treatment and support health care professionals (HCPs) to provide comprehensive and personalized diagnostic and therapeutic processes. Therefore, several handheld and wearable devices, implantable loop recorders (ILRs), mobile platforms, and support systems have been developed to support detection and integrated AF management. However, many of the available mHealth solutions are not clinically validated. Hence, caution is needed in their clinical use. To date, no systematic review has comprehensively evaluated the impact of the variety of mHealth tools developed for patients with AF and HCPs who manage this condition. In this systematic review article, we summarize the available literature on mHealth solutions including handheld and wearable devices, ILRs (Fig. 1), as well as mobile platforms and support systems in AF detection and management.

## Methods

### Search strategy

This systematic review was conducted in accordance with the Preferred Reporting Items for Systematic Reviews and Meta-analyses (PRISMA) guidelines [5]. The electronic databases PubMed (NCBI), Embase (Ovid), and Cochrane were systematically searched for articles published until 10 February 2021, inclusive. The main search strategy is available in Table S1 (supplementary material online).

### Eligibility criteria

We included case–control, cohort, and cross-sectional studies that evaluated the effects of mHealth solutions designed to screen and monitor AF, enhance patient’s and/or HCP’s education of AF, improve communication between AF patients and HCPs, or to encourage active AF patient involvement in the management of their condition. Randomized and non-randomized controlled trials (RCTs) were only considered if demographic and outcome data were available. We excluded duplicates, published conference abstracts, case reports, studies without original data (e.g., reviews, commentaries, editorials), non-English written articles, and studies that only included routine methods of cardiac monitoring (pacemaker, cardiac resynchronization therapy, implantable cardioverter defibrillators). We included both invasive and non-invasive technologies since there is growing number of invasive tools that could be managed remotely by Bluetooth technology.

### Data extraction

All identified studies were screened based on their title and abstract against the search criteria by two reviewers (A.N.L.H. and M.G.). The search was supplemented by manually screening the reference lists of the articles that were selected based on the search. The full texts of all articles were independently assessed by both reviewers and if they still met the eligibility criteria, the manuscript was included. Disagreements were resolved through assessment by a third reviewer (L.D.).

### Data synthesis

Given that the included studies varied widely in their design, interventions, comparators, and outcomes, no synthesis was undertaken, and we undertook a narrative review.

## Results

**Fig. 2:**
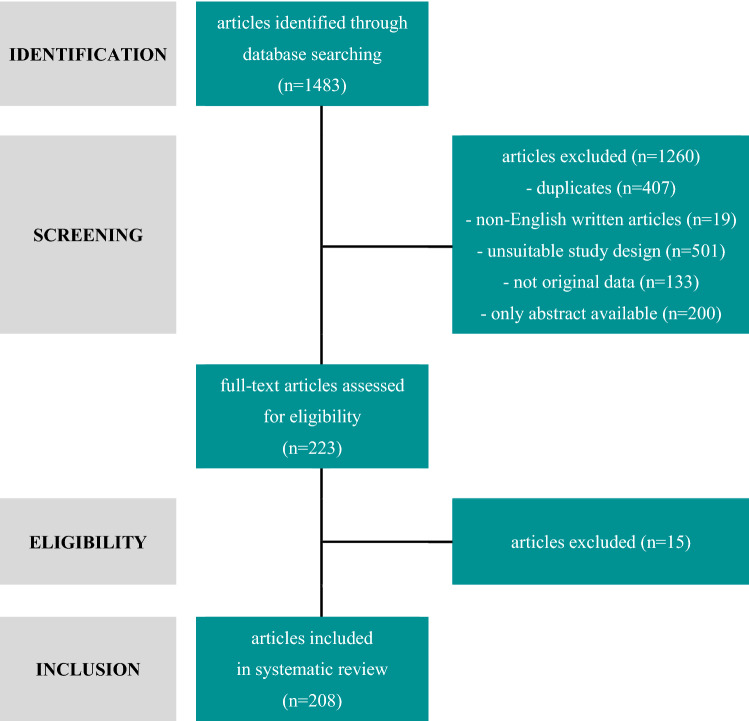
Flow diagram for study selection process

**Fig. 3:**
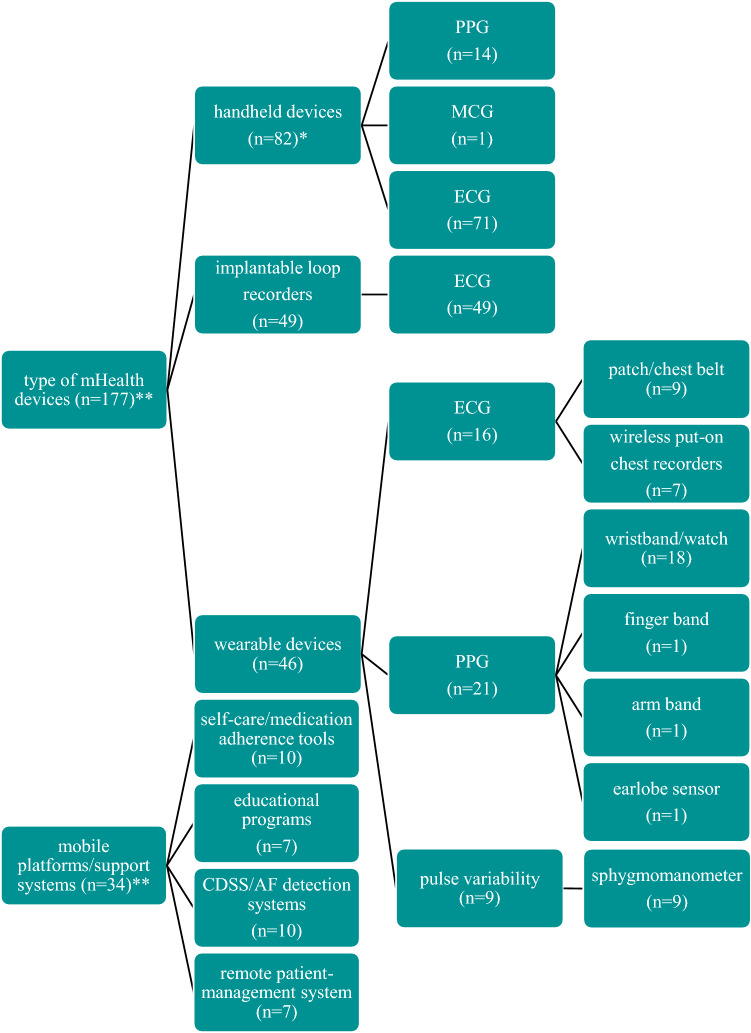
Type of mobile health solutions for atrial fibrillation detection and management. *Numbers do not add up to 82 as 4 studies assessed in parallel PPG- and ECG-based device, **numbers do not add up to 208 as 3 studies assessed in parallel handheld device with mobile platforms/support systems (*n* = 1) and wearable devices (*n* = 2). *AF* atrial fibrillation, *CDSS* clinical decision system support, *ECG* electrocardiography, *MCG* mechanocardiography, *mHealth* mobile health, *PPG* photoplethysmography

We identified 1483 studies (Fig. 2). After exclusion of duplicates (*n* = 407), non-English written articles (*n* = 19), studies with unsuitable study design (*n* = 501), not original data (*n* = 133), and articles without full text availability (*n* = 200), the titles, and abstracts of 223 articles were independently assessed for eligibility in their full text. Of these, 208 were deemed potentially relevant. A full list of the excluded studies after full-text reading and the reason for exclusion are provided in Table S8 (supplementary material online). Of the 208 studies included, 82, 46, and 49 studies aimed at validating handheld devices, wearables, and ILRs for AF detection and/or management, respectively, while 34 studies assessed mobile platforms/support systems (Fig. 3).

### Handheld devices

A handheld device is a piece of computing equipment that can be used by holding in hand or touching it by a finger, activated by user and capable of detecting, analyzing and transmitting information concerning body signals with provided biofeedback. There are three technologies available to detect and monitor AF by a handheld device or mobile phone: photoplethysmography (PPG)-, electrocardiography (ECG)-, and mechanocardiography (MCG)-based devices [4]. Brief population characteristics of the studies on handheld devices, including their sensitivity and specificity, for AF detection and monitoring are presented in Table S2 (supplementary material online).

#### Photoplethysmography-based devices

PPG technology is an optical technique that uses blood volume changes in the microvascular tissue bed that directly reflects pulse morphology. In PPG recordings, AF manifests as varying pulse-to-pulse intervals and pulse morphologies [4].

Despite the availability of multiple PPG-based mobile apps, only a few have been validated. Clinical validation studies (*n* = 14) have been performed for FibriCheck [6–8], CardiioRhythm [9–11], Preventicus [12–16], and PULSE-SMART [17]. Majority (10 [71%]) of the studies were prospective cohort studies. One study (7.7%) was a RCT. Studies included between 88 and 10,000 participants with a mean age between 49 and 78 years, and with a percentage of females ranging from 35 to 100%. Only FibriCheck is currently cleared by the Food and Drug Administration (FDA) and Preventicus is in the FDA certification process. Both FibriCheck and Preventicus had Conformité Européenne (CE) approval. All mobile phone applications use the fingertip to measure PPG signals. Only CardiioRhythm can also derive PPG signals from the face. The duration of one PPG recording differs between the mobile applications: FibriCheck and Preventicus use 60-s recordings for heart rate and rhythm assessment, CardiioRhythm records for 20 s, and PULSE-SMART for up to 2 min. The Preventicus app also provides the option to extend measurements up to 5 min to enable less frequent arrhythmic events to be recorded more accurately. All apps provide the possibility to combine heart rhythm monitoring with symptom annotation and tracking. Secure cloud solution allowing PPG recordings storage and access for both patients and their HCPs is available for all four abovementioned apps. In the case of FibriCheck and Preventicus, there is the possibility for a medical team of experts to perform an evaluation of the measurements to exclude possible measurement errors and to verify heart rhythm disturbances from a medical-technical point of view. Additionally, they provide a structured and detailed report in a standardized format, which may be shared with HCPs and allows implementation in a digital patient record. Despite some feature differences, sensitivity and specificity for all aforementioned applications are high, with the highest sensitivity reported for PULSE-SMART (97.1%) and the highest specificity reported for Preventicus (98.1%). However, the sensitivity and specificity reported for the different devices are based on simultaneous disposable PPG and ECG recordings (from 1 to 6 repetitions). Data on the accuracy for longitudinal heart rhythm monitoring are lacking. The recording time (ranging from 20 s to 5 min) did not impact the accuracy to detect AF. Only one study compared a 1-min measuring period to a 5-min measuring period and demonstrated no impact on sensitivity and specificity, while the signal quality decreased from 93.3% (1-min test) to 67.7% (5-min test) [12]. Studies in which a 12-lead ECG was used as reference to assess the sensitivity and specificity of the respective app revealed a slightly higher range of sensitivity as compared to those where single-lead ECG was used as the reference (sensitivity: 93.1–98% vs 89.9–95.4%; specificity: 88–96.2% vs 85–99.6%). The highest sensitivity was observed among the elderly population (98%), whereas the highest specificity was observed among hospitalized patients at cardiology/geriatric wards (99.7%). The accuracy to detect AF was comparable for on-demand PPG-based handheld devices and continuous heart rate and rhythm monitoring by wearable PPG-based devices (e.g., wristbands) (sensitivity: 95.4% vs 95%, specificity: 99.7% vs 99.7%, respectively) [13]. Moreover, the sensitivity to detect AF by PPG-based handheld devices seems to be equal to (both 98%) [7] or even higher (92.9% vs 71.4%) than ECG-based handheld devices with comparable specificity (88–97.7% vs 85–99.4%) [18].

#### Electrocardiography-based devices

MyDiagnostick [19, 20]^1^ [21–27] and KardiaMobile [7, 11, 16], [24, 28–75] represent the most widely used examples of ECG-based devices. Limited data are available for additional devices, such as DigiO2 Cardio Care ECG recorder [76], Zenicor-EKG [77–79], Card Guard [80], Sensor mobile 100 [81, 82], CardioBip [83, 84], and ECG check [85]. Overall, clinical validation studies (*n* = 71) have been performed, of which 54 (76%) were prospective cohort studies and 10 studies (14%) were RCTs. Studies included between 21 and 1,952,811 participants with a mean age between 40 and 79 years, and with a percentage of females ranging from 16 to 80%.

MyDiagnostick is a CE-marked, stick-shaped handheld device intended to detect AF within 45–60 s by holding both metallic handles. After recording, physicians can review, share, and store the ECG data by connecting the device via USB to a computer. Data can be analyzed by a standalone PC application or an internet-based web portal [27]. KardiaMobile is an FDA/CE-approved handheld tool that converts electrical ECG signals, from electrodes located on a metallic plate after 30-s finger touch to ultrasound signals, and transmits these signals to a smartphone as a single-lead ECG. Importantly, KardiaMobile 6L provides a third electrode which can be put on the left knee or ankle to imitate the 6 classic limb leads of a full 12-lead ECG. The recorded data are stored in an encrypted cloud accessible for the patient and HCP.

Most of the available studies regarding MyDiagnostick were performed for AF screening [19], [21–23, 25–27, 86, 87]; only one study used this device to monitor patients with recent-onset AF treated with cardioversion or rate control medication [20]. Overall diagnostic sensitivity and specificity ranged from 60.5 to 100% and 93 to 97.3%, respectively. The highest sensitivity was reported among patients admitted to cardiology outpatient clinics (100%) and specificity in those hospitalized in geriatric clinics (97%). In all analyzed studies, heart rate and rhythm measurements were performed at a single time with 12-lead ECG as gold standard for device accuracy.

The number of studies describing the diagnostic accuracy of KardiaMobile is almost five times higher compared to the studies on MyDiagnostick, which could explain the wide range of diagnostic sensitivity (38–100%) and specificity (29.2–100%) of KardiaMobile, potentially impacted by the heterogeneity of included patient groups. Most of the studies assessed accuracy of KardiaMobile to detect AF by 1–2 measurements at a single time point. Only three studies assessed sensitivity and specificity of KardiaMobile during more than one day, demonstrating a sensitivity of 94.6% and specificity of 92.9% in case of 4 measurements per day for 1 month [88], a sensitivity and specificity of 95.3% and 97.5% in case of 3 standard measurements per day and additional measurements in case of symptoms for 1 month [73], and a sensitivity of 38% in case of 2–3 measurements per day for 5 days [53]. Taking patient selection into account, the highest sensitivity and specificity were observed in elderly patients (aged ≥ 65 years). Higher sensitivity and specificity range were observed if a 12-lead ECG was used as gold standard compared to “expert” diagnosis (54.5–100% vs 38–100% and 65–100% vs 29.2–99%, respectively). To date, only one study has performed a direct comparison of MyDiagnostick and KardiaMobile for AF detection in both cardiology and geriatric wards showing suboptimal sensitivity and specificity values for both devices (cardiology ward: 81.8 and 94.2%, respectively, for MyDiagnostick; 54.5 and 97.5%, respectively, for KardiaMobile; geriatric ward: 89.5 and 95.7%, respectively, for MyDiagnostick; 78.9 and 97.9%, respectively, for KardiaMobile) [24].

#### Mechanocardiography-based devices

MCG-based apps record mechanical cardiac activity via accelerometers and gyroscopes, registering the tiny cardiogenic micro movements of the patient’s chest for signal acquisition [4]. The data on this technology are limited; to date only one study has evaluated this method for AF detection. This study included 300 participants (median age 75, 44% women) and demonstrated a low sensitivity (67%) and high specificity (99%) when compared to 5-lead telemetry ECG [89]. Further studies are needed to establish this method for accurate AF detection.

### Wearable devices

Wearables are lightweight, sensor-based devices, which are worn close to and/or on the surface of the skin, where they detect, analyze, and transmit information continuously or on-demand concerning body signals to an external device and provide biofeedback. These devices are becoming increasingly popular and encompass a wide range of PPG-based devices, including wristwatches/bands, armbands, fingerbands, and earlobe sensors, ECG-based devices, including patches, chest belts, and wireless recorders, as well as pulse variability-based devices, such as sphygmomanometers. Brief population characteristics of the studies on wearable devices are presented in Table S3 (supplementary material online).

#### Photoplethysmography-based wearables

Overall, clinical validation prospective cohort studies (*n* = 21) have been performed, included from 20 to 419,297 participants with a mean age between 41 and 76 years, and with a percentage of females ranging from 15 to 49%. Most studies using wristwatches/bands, armbands, and fingerbands incorporating PPG technology have focused on AF detection [90–105], and only a few on heart rate monitoring [106–110]. Most devices can be worn around the wrist, while Everion^®^ is worn on the upper arm and CardioTracker as a ring on the finger. Apple Watch, Fitbit, and Empatica E4 were cleared by CE and the FDA, and CardiacSense is in the advanced stage of FDA and CE certification. A secure cloud solution allowing storage of PPG recordings and access for patients is available for Apple Watch, Fitbit, CardiacSense, Samsung Simband, Empatica E4, Gear Fit 2, Wavelet Health, Amazfit, Honor Band 4, Huawei Watch GT, and Honor Watch. In the case of Samsung Simband, there is the possibility to ask a medical team of experts about specific health questions and to perform an evaluation of the measurements to exclude possible measurement errors and to verify heart rhythm disturbances from a medical-technical point of view.

Overall sensitivity and specificity for all validated wristwatches/bands, armbands, and fingerbands are high and range from 67.7 to 100% and 60.7 to 100%, respectively, with the highest sensitivity of CardiacSense (100%) [93] and of Honor Band 4, Huawei Watch GT, and Honor Watch (100%) [97], and specificity of CM3 Generation-3 (100%) [95]. However, reported sensitivity and specificity are based on different monitoring periods that vary from 60 s [100] to 855 h [95]. Interestingly, despite differences in measurement times, the accuracy to detect AF using these wearables was comparable between all studies. Additionally, accuracy levels of the wristbands/watches [90–101, 105–110], fingerbands [102], and upper armbands [103] and PPG-based earlobe sensor [104] were similar. Studies in which a 24-h Holter monitor was used as a reference to assess the wearable’s specificity for AF detection revealed a slightly higher range of specificity when compared to those where 12-lead ECG or single-lead ECG was considered the gold standard (84.9–100% vs 67.6–99% and 60.7–100%, respectively). Studies in which single-lead ECG was used as a reference to assess the wearable’s sensitivity revealed a slightly higher range of sensitivity when compared to those where 24-h Holter or 12-lead ECG was considered as the gold standard (79–99% vs 71.6–95.2% and 67.7–100%, respectively). Two studies directly compared the diagnostic accuracy of PPG-based wristwatches/bands and ECG-based wristbands. The study by Chen found a higher sensitivity and a lower specificity for wristwatches/bands using PPG compared to wristbands using ECG (88% vs 87.3% and 96.4% vs 99.2%, respectively) [99]. In contrast, in the study by Selder, PPG-based wristwatches/bands had a lower sensitivity and a similar specificity compared to ECG-based wristbands (79% vs 93% and 98% vs 98%, respectively) [100]. Further research is warranted to further investigate the diagnostic accuracy of PPG-based and ECG-based wristwatches/bands, armbands, and fingerbands in a selective high-risk population.

#### Electrocardiography-based wearables

Overall, clinical validation studies (*n* = 16) have been performed, of which 13 (81%) were prospective cohort studies with no RCTs. Studies included between 10 and 27,841 participants with a mean age between 53 and 66 years, and with a percentage of females ranging from none to 56%. Patch-based wearables record ECG signals without visible electrodes and lead wires. Eight studies focused on AF detection using patch-based devices, such as Zio^XT^ [111–114], RhythmPad [115], and Firstbeat Bodyguard 2 [57], which were all CE approved. Only Zio^XT^ was cleared by the FDA. Most patches provide single-lead or 3-lead ECG recordings and are attached to the patient’s chest, whereas RhythmPad consists of 3 sensors placed around both arms and the right leg and records a 6-lead ECG. A patch can be used for uninterrupted heart rhythm monitoring for different time periods [from 10 s (RhythmPad) to 2 weeks or even longer (Zio^XT^)]. Several patches have the possibility to combine heart rhythm monitoring with symptom annotation, as they contain a trigger button that can be pressed when patients experience symptoms. A secure cloud solution allowing ECG recordings storage and access for both patients and HCPs is available for Zio^XT^, RhythmPad, and Firstbeat Bodyguard 2. Only Zio^XT^ has the possibility to provide structured and detailed reports in a standardized format that could be shared with HCP and implemented in a digital patient record.

Two studies investigated the diagnostic accuracy of patches in AF detection [57, 115]. Overall diagnostic sensitivity and specificity of RhythmPad and Firstbeat Bodyguard 2 ranged from 93.4 to 96.3% and 96.8 to 98.8%, respectively, with the highest sensitivity of Firstbeat Bodyguard 2 (96.3%) and specificity of RhythmPad (98.8%). However, reported sensitivity and specificity levels are based on short continuous heart rhythm monitoring, ranging from 10 s to 2 min. Data on patches’ long-term accuracy are lacking. The highest sensitivity was observed in patients above 65 years of age, whereas the highest specificity was observed in patients at high AF risk. Accuracy of both aforementioned patches was determined using 12-lead ECG as the gold standard. The accuracy level of ECG-patches is comparable with the sensitivity and specificity of ECG-based chest belt devices (ranging from 96.3 to 97% and 95.6 to 98.2%, respectively) [57, 116]. Additional research is needed to establish the accuracy level of patches in detection of AF.

Wireless recorders enable automatic arrhythmia detection and transmission of patient data files to service centers for immediate analysis and physician attention. ECG data of most wireless recorders are stored in an encrypted cloud. Transmission occurs without patient interaction. Wireless recorders can provide single-, 3-, or 12-lead ECG recordings. In several studies [117–122], wireless ECG recorders were used for short- and long-term continuous monitoring (ranging from 4 min to 24 h) and for long-term intermittent monitoring using single-lead ECG (ranging from 30 s daily for 4 weeks to 30 s twice daily for 6 months). Despite several ECG-based wireless recorders being available, only Medi-Trace 200 has been validated in clinical studies and had FDA and CE approval. The study of Lin revealed a high sensitivity of 94.6% and positive predictive value of 99.4% for Medi-Trace 200 in patients with and without a coded diagnosis of AF [117]. Additional research is warranted to establish the accuracy level of wireless ECG recorders in detection of AF in comparison to other wearable ECG- or PPG-based devices.

#### Pulse variability-based wearables

Overall, clinical validation prospective cohort studies (*n* = 9) have been performed that included between 73 and 2052 participants with a mean age between 58 and 80 years, and with a percentage of females ranging from 35 to 63%. Sphygmomanometers are automatic upper arm or wrist oscillometric blood pressure monitors, which can incorporate an algorithm for AF detection. Based on sphygmomanometer recordings, AF is concluded if at least two of three measurements show pulse irregularities. Nine studies focused on AF detection using sphygmomanometers [60, 123–130]. The Microlife BP [60, 124, 126–130] and OMRON [125, 130] have been validated in these clinical studies, and both have FDA and CE approval. Secure cloud solution allowing PPG recordings storage and access for patients and HCPs is available for both types of sphygmomanometers. In addition, they provide structured and detailed reports in a standardized format, which could be shared with HCPs and implemented in a digital patient record. Despite some differences in features, overall sensitivity and specificity for the validated sphygmomanometers are high, ranging from 83.3 to 100% and 88.8 to 98.8%, respectively. Most reported sphygmomanometers’ sensitivity and specificity are based on single measurements (from 1 to 3 repetitions), and only Wiesel et al. provided long-term intermittent accuracy data of sphygmomanometer (4 repetitions per day for 30 days) [124]. Irrespective of measurement time, the accuracy levels of sphygmomanometers to detect AF were comparable. The study in which single-lead ECG was the reference to assess sphygmomanometer’s accuracy [124] revealed a higher sensitivity and a slightly lower specificity as compared to those in which 12-lead ECG was considered as the gold standard (99.2% vs 94.6 and 92.9% vs 93.4%, respectively). A sphygmomanometer is a promising technology for the first step of AF screening. As hypertension is a major modifiable risk factor for AF, using a sphygmomanometer for AF detection would be valuable for the large number of hypertensive patients who monitor their blood pressure. To date, only one study examined the diagnostic accuracy of a sphygmomanometer in hypertensive patients. Therefore, additional research on accuracy of sphygmomanometer in AF detection in this specific population would be interesting.

### Implantable loop recorders

ILRs are small devices inserted beneath the skin of the chest. Once implanted, the devices automatically capture continuous ECGs or can be activated manually by the patient if symptoms occur using optional external handheld patient devices or smartphone applications (Fig. 3). Wireless technology enables communication between the ILR and the clinician programmer, smartphone, or tablet. The information from the device is used to regularly inform the patient about abnormal heart rhythm, evaluate symptom–rhythm relations and, through manual activation, enable patients to self-management. ILR detection parameters, data storage, and methods of data transmission are presented in Table S4 (supplementary material online). The most frequently used ILRs are the Reveal [44, 131–172], BioMonitor [140, 173–175], and Confirm [146, 176, 177].

Overall, clinical validation studies (*n* = 49) have been performed, of which 30 (61%) were prospective cohort studies with 4 (8.2%) RCTs. Studies included between 30 and 1247 participants with a mean age between 49 and 76 years, and with a percentage of females ranging from 9 to 53%. Brief population characteristics of the ILR studies are presented in Table S5 (supplementary material online). Most of the included studies reported different lengths of follow-up, but most patients were diagnosed with AF within the first 6 months [150, 155, 167]. In a study by Healey, the AF detection rate roughly doubled by 6 months compared to 1 month (64% versus 34%) [176]. This is in line with the CRYSTAL-AF study where the AF detection rate increased from 8.9 to 12.4% and 30% at 6-, 12-, and 30-month follow-up, respectively [168]. Interestingly, most detected AF episodes were asymptomatic [145, 150, 164] and would probably have been missed without continuous ILR monitoring.

None of the studies provided comparative diagnostic test accuracy between a group of patients who were monitored with an ILR and a group that received standard monitoring. However, two studies [155, 158] used traditional AF detection data for a group of patients who were monitored for AF allowing the estimation of the diagnostic accuracy. The study of Choe found sensitivities of between 1.3%, from a single 24-h Holter monitor, and 20.8%, from quarterly 7-day Holter monitoring [158], which was confirmed in a study by Ziegler (24-h Holter monitor: 2.9%; quarterly 7-day Holter monitoring: 22.9%) [155]. Therefore, even the best-performing intermittent monitoring strategy detected less than one-third of the AF detected by ILR. Although ILR is often used as the gold standard to determine the diagnostic accuracy of other AF detection monitors, some studies [140, 157] reported an up to 90% false-positive rate for the Reveal and BioMonitor [140]. From 15% (subpectoral) to 46% (subcutaneous ILR localization Reveal) [157] of all AF episodes detected by the ILR algorithm were not subsequently verified as AF by a reviewing HCP. The mixed population diagnostic test accuracy studies suggest that the performance of the AF diagnosis algorithm in the Reveal XT and Reveal LINQ to diagnose AF improved over time. The study of Pürerfellner used the XPECT trial and Reveal LINQ usability study data sets and reanalized the data by a new ILR AF detection algorithm also incorporating the detection of P-waves [147]. They demonstrated that the accuracy of the Confirm DM2102 and Reveal LINQ improved by the adaptive P-sense algorithm (TruRhythm) to 100% sensitivity for AF detection, while the specificity varied (85.7% and 99.0%, respectively). Data on the diagnostic accuracy of the new version of the BioMonitor (BioMonitor 2-AF) and the Confirm (Confirm Rx) devices are still lacking (Table S4, supplementary material online). High-quality head-to-head clinical trials of the Reveal, BioMonitor, and Confirm are required to enable a direct comparison between the ILR in terms of clinical effectiveness.

### Mobile platforms and support systems

The adoption and use of mobile devices (smartphones, watches or tablets) is widespread, with 88% of all users spending time in mobile applications [178]. Over 318 000 mobile applications are now available worldwide [179], including more than 500 dedicated to AF management [180]. Potential uses of those tools in daily practice include self-care/medication adherence tools [29, 181–189], educational programs [190–196], clinical decision support systems/AF detection systems [197–206], and remote patient–management systems [207–213]. Despite a widespread availability, most of the AF mobile platforms and support systems are not evaluated for effectiveness and lack regulatory oversight [214]. To date, only a minority of them are FDA/CE-approved (Table S6, supplementary material online) and/or evaluated in clinical studies Table S7 (supplementary material online). In a recent review of mobile applications for the detection and management of AF, the most common app functionalities were capturing and graphically displaying user self-reported and self-entered data (75%) and PPG waveform monitoring (92%). However, only 42% were scored above average for quality (MARS score ≥ 3.0) [180]. This highlights the need for clinically validated mobile applications to support patients and HCPs in the management of AF.

#### Self-care/medication adherence tools

Ten studies (60% prospective cohort studies and 40% RCTs) validated mobile app dedicated to comprehensive AF management, including between 10 and 2473 participants (mean age range: 59–69 years; female percentage range: 33–50%). The Health Buddies application was developed to improve adherence to oral anticoagulation in an elderly AF patients spelling out daily challenges for them and their grandchildren. Three-month study duration resulted in a mean increase in AF knowledge level of 5.8%, whereas anticoagulation adherence was as high as 99% [181]. Computer-animated application, designed by Magnani to improve patient education on AF, medication adherence and symptom management, significantly improved quality of life based on AF Effect on Quality of life (AFEQT) score from 64 to 76%, and medication adherence based on the Morisky 8-item Medication Adherence Scale (MMAS-8) from 7.3 to 7.7 during relatively short time [29].

Several other applications have been developed to enhance patient education, improve communication between patients and HCP, and encourage active patient involvement. The mobile AF application (mAFA-II) trial [183, 215] reported that this holistic app-based management with dynamic risk monitoring and reassessment of the bleeding and thromboembolic risk scores reduced the risks of bleeding (mAFA vs usual care, 2.1% vs 4.3%) [188] and clinical adverse events, including thromboembolic events, rehospitalization, and all-cause death (1.9% vs 6.0%) [187], and increased total oral anticoagulation usage from 63 to 70% [188]. Continuous home monitoring with PPG technology via mAFA recognized AF with a positive predictive value (PPV) of 91.6% [216] suggesting feasibility of this approach for AF screening.

Based on available data, clinicians agreed that mobile applications facilitate AF patient’s management with low decisional conflict [186, 213], whereas patients reported an improvement in their quality of life [29, 183, 213], AF- and procedure-related knowledge [186, 195], and medication adherence [29, 181, 183, 184, 186, 213].

#### Educational programs

Seven studies (2 [29%] prospective cohort studies and 5 [71%] RCTs) validated mobile app dedicated to comprehensive AF management, including from 12 to 720 participants (mean age range: 30–72 years; female percentage range: 24–82%). The OCULUS study was aimed to evaluate the effectiveness of the three-dimensional movie in teaching patients about AF associated consequences and stroke prevention. Patient AF risk and anticoagulation knowledge increased from 70 to 96% and from 22 to 83%, respectively, immediately after movie-based education and remained stable after 1 year [190]. Online tailored education of AF patients, requiring cardioversion or pulmonary vein isolation, improved their procedure knowledge from 65 to 75% based on the Jessa AF Knowledge Questionnaire (JAKQ), and this knowledge persisted at 6 (78%) and 12 (80%) weeks after the AF-related procedures [191]. EVICOAG, a Qstream spaced education platform comprising 12 case-based AF and anticoagulation learning scenarios, improved overall knowledge scores by 54% and use of the CHA_2_DS_2_-VAS_c_ and HAS-BLED scores among nurses during 6-week education [194]. Graded systematic exposure to online automated webinar dedicated for electrophysiologists improved their baseline identification of AF source on panoramic AF maps by 13% [195].

#### Clinical decision support systems/atrial fibrillation detection systems

Ten studies (30% prospective cohort studies and 60% RCTs) validated mobile app dedicated to comprehensive AF management, including from 60 to 13,379 participants (mean age range: 44–73 years; female percentage range: 23–44%). Discovery Link AFinder, a web-based application scanning all CareLink^®^ Network transmissions to identify patients with AF among those with cardiac implantable electronic devices, enhanced AF detection sensitivity by 10% and improved oral anticoagulation optimal treatment by 6% [197]. A shared decision-making interaction, facilitated by Atrial Fibrillation Shared Decision Making (AFSDM) [199], Clinical Decision Support for AF (CDS-AF) [202], and Decision Analysis in Routine Treatment Study (DARTS) [205], decreased the rate of discordant antithrombotic therapy leading to improved medication adherence and patient satisfaction. AKENATON, an artificial intelligence tool to filter AF alerts, resulted in an 84% reduction in notification workload, while preserving patient safety [203].

#### Remote patient–management systems

Seven studies (2 [29%] prospective cohort studies and 5 [71%] RCTs) validated mobile app dedicated to comprehensive AF management including from 10 to 2281 participants (mean age range: 61–74 years; female percentage range: 32–48%). In a study by Shacham, instructions delivered by telephone to patients supported a conversion rate of almost 80% of AF episodes, whereas additional interventions by an attending physician within a mobile intensive care unit resulted in a conversion rate of only 70% [209]. Recently, the Characterizing AF by Translating its Causes into Health Modifiers in the Elderly (CATCH ME) Consortium, in collaboration with the European Society of Cardiology (ESC), has funded the creation of two applications for patients with AF and their HCPs. The patient application (myAF) aims to enhance patient education, self-management and interaction with HCPs, and the HCP application simplifies the choice of treatment and optimizes AF guideline adherence [217]. AF educational intervention within Integrated Management Program Advancing Community Treatment of AF (IMPACT-AF) trial [212] resulted in a significant increase in the proportion of oral anticoagulation use by AF patients and reduction in thromboembolic events during 12-month observation.

## Conclusive remarks and perspectives

In this systematic review we have analyzed 208 studies. Compared to other systematic reviews that focused only on diagnostic accuracy of mHealth devices in screening for and detecting AF [218, 219], platforms, and programs to improve patients’ knowledge of AF [220], we performed comprehensive summary of available mHealth devices, mHealth platforms, and mHealth applications for the screening, detection and management of AF for the first time.

The diagnostic accuracy of mHealth devices differs with respect to the type (handheld vs wearable vs ILR) and technology (ECG vs PPG-based devices) used. Based on the current international AF management guidelines of the ESC, ECG confirmation (even single-lead ECG of 30 s or more) is mandated for the diagnosis of AF [3]. PPG technology is not sufficient to diagnose AF based on current ESC guidelines [221]. However, there are already some data demonstrating that PPG technology is nearly as accurate as ECG to detect AF [18], [222]. The ongoing randomized-controlled Heartline Study (NCT04276441) will additionally investigate whether PPG-based devices could reduce thromboembolic events by early AF detection. Most of the ECG- and PPG-based algorithms are validated to detect AF with a high sensitivity and specificity, as most algorithms are developed for AF screening scenarios [223].

mHealth solutions with the same technologies may collect data in different ways (intermittent vs continuous, spot vs longitudinal assessment) as well as different methods of measurement (handheld vs wearable device) which could influence the sensitivity and specificity for AF detection [224]. In addition, AF burden and AF density, which both are gaining importance in the evaluation of AF treatment efficacy, can only be assessed by continuous longitudinal heart rhythm monitoring. Intermittent longitudinal heart rhythm monitoring, as provided by most mHealth handheld devices and wearables, may represent a surrogate variable of true AF burden and density. An important limitation in the utilization of user-controlled devices is the potential underdiagnosis of subclinical and asymptomatic arrhythmias, which do not trigger a rhythm documentation by the wearer. Since AF is often asymptomatic, especially in the early stages of paroxysmal AF, the use of mHealth devices in these subjects can only be applied with caution. Therefore, there is a need to perform head-to-head comparisons between handheld vs wearable devices, as their comparative effectiveness is limited, hence unclear.

To enable a smooth flow of information between the patient and the HCP, a shared infrastructure, in most cases a secured and certified cloud, is crucial to make patient data remotely available. A physician-initiated or at least guided approach appears to be necessary to allow personalized mHealth use and the selection of the right tool for each patient. Moreover, in addition to heart rate and rhythm monitoring, which can be performed by several mHealth solutions, the KardiaMobile and the Apple Watch allow a more detailed ECG interpretation incorporating QRS duration and QT interval analysis, [225–228] supraventricular tachycardia differentiation [229], and estimation of potassium levels by changes in T-wave morphologies [230].

Although mHealth solutions are becoming increasingly popular in the detection and management of AF, there are several barriers to widespread mHealth adaption in healthcare systems. Until reimbursement will not be provided by health insurances or the government, use of mHealth solutions will be limited to those patients who are willing to pay out-of-pocket for such a support, which may contribute to digital and mHealth inequity and fragmented care. Based on our review the studies which used randomly selected, mostly, low-risk population, reported the lowest accuracy for AF detection, whereas those focused on AF screening among high-risk and/or elderly population, reported the highest sensitivity and specificity for AF detection. Therefore, more targeted population selection and identifying those patients at higher risk of AF by using specific biomarkers [79] is a reasonable way to boost the pre-test, hence reduce false-positive results, especially among populations that are not represented by the usual risk scores (especially CHADS_2_ and CHA_2_DS_2_-VASc). Furthermore, more studies assessing therapeutic consequence results from an incidentally diagnosed AF in this population are on highly importance. Additionally, no standards for minimal requirements of validation studies and the format of data reports have been agreed on. This results in heterogeneous data collection processes with various devices and technologies of different reliability and validity complicating the implementation of mHealth-based results into healthcare system. Some infrastructures have been developed to manage the enormous amount of data supplied by mHealth tools to extract the most important data for the decision-making processes, but the concern over privacy is also legitimate. Sensitive health data of patients are exchanged through wireless networks and thus addressing the privacy and security concerns in the usage of mHealth apps is essential. Regulators must develop standards that the developers and all involved stakeholders need to adhere to in order to ensure data privacy and security in the healthcare system. Involvement of HCPs in the evaluation of functionality, usability, and security will enhance the trustworthiness of the apps and increase their adoption. A good mHealth policy should inform the users of what data are collected, how it is stored and used. This will enable users to weigh the benefits and risks of specific mHealth apps. All involved stakeholders, including HCPs, patients, mHealth companies, and health insurance companies, also need to discuss and agree on how best to use and implement mHealth solutions in clinical care in the future. Initiatives, such as the TeleCheck-AF project, that was introduced to keep AF patients’ care during the coronavirus-2019 pandemic via teleconsultations coupled with heart rate and rhythm monitoring [231, 232], will help step-by-step to implement tailored mHealth solutions in clinical care pathways.

## Strengths and limitations

This is the first systematic review of studies evaluating mHealth devices and applications in screening, detecting, and managing AF, aimed to support clinicians in choosing the appropriate way of monitoring patients and educating them in everyday practice, and to support researchers in finding research directions that should be extended.

Nonetheless, several limitations should be noted. The variation in interventions, settings, and study designs precluded meta-analyses. The methodological quality of the studies was suboptimal and prone to bias as most studies had an observational or quasi-experimental design and only a minority of studies were randomized control trials.

## Supplementary Information

Below is the link to the electronic supplementary material.

Supplementary file1 (PDF 1327 KB)

Supplementary file2 (DOCX 41 KB)

## Footnotes

1 References numbered 21–232 are included in supplementary material online.

## References

[1] Chugh SS, Roth GA, Gillum RF, Mensah GA (2014). Global burden of atrial fibrillation in developed and developing nations. Glob Heart.

[2] Dai H, Zhang Q, Much AA, Maor E, Segev A, Beinart R (2020). Global, regional, and national prevalence, incidence, mortality, and risk factors for atrial fibrillation, 1990-2017: results from the Global Burden of Disease Study 2017. Eur Heart J Qual Care Clin Outcomes.

[3] Hindricks G, Potpara T, Dagres N, Arbelo E, Bax JJ, Blomstrom-Lundqvist C (2020). ESC Guidelines for the diagnosis and management of atrial fibrillation developed in collaboration with the European Association of Cardio-Thoracic Surgery (EACTS). Eur Heart J.

[4] Moher D, Liberati A, Tetzlaff J, Altman DG, Group P (2009). Preferred reporting items for systematic reviews and meta-analyses: the PRISMA statement. BMJ.

[5] Proesmans T, Mortelmans C, Van Haelst R, Verbrugge F, Vandervoort P, Vaes B (2019). Mobile phone-based use of the photoplethysmography technique to detect atrial fibrillation in primary care: diagnostic accuracy study of the FibriCheck app. JMIR mHealth uHealth.

[6] Van Haelst R (ed) (2017) The diagnostic accuracy of smartphone applications to detect atrial fibrillation: a head-to-head comparison between Fibricheck and AliveCor. Acta Cardiologica. Taylor & Francis Ltd 2-4 Park Square, Milton Park, Abingdon OR14 4RN, Oxon, England: Taylor & Francis Ltd

[7] Verbrugge FH, Proesmans T, Vijgen J, Mullens W, Rivero-Ayerza M, Van Herendael H (2019). Atrial fibrillation screening with photo-plethysmography through a smartphone camera. EP Europace.

[8] Yan BP, Lai WH, Chan CK, Chan SCH, Chan LH, Lam KM (2018). Contact-free screening of atrial fibrillation by a smartphone using facial pulsatile photoplethysmographic signals. J Am Heart Assoc.

[9] Rozen G, Vaid J, Hosseini SM, Kaadan MI, Rafael A, Roka A (2018). Diagnostic accuracy of a novel mobile phone application for the detection and monitoring of atrial fibrillation. Am J Cardiol.

[10] Chan PH, Wong CK, Poh YC, Pun L, Leung WWC, Wong YF (2016). Diagnostic performance of a smartphone-based photoplethysmographic application for atrial fibrillation screening in a primary care setting. J Am Heart Assoc.

[11] Brasier N, Raichle CJ, Dörr M, Becke A, Nohturfft V, Weber S (2019). Detection of atrial fibrillation with a smartphone camera: first prospective, international, two-centre, clinical validation study (DETECT AF PRO). Ep Europace.

[12] Fan Y-Y, Li Y-G, Li J, Cheng W-K, Shan Z-L, Wang Y-T (2019). Diagnostic performance of a smart device with photoplethysmography technology for atrial fibrillation detection: pilot study (Pre-mAFA II registry). JMIR mHealth uHealth.

[13] Birkemeyer R, Müller A, Wahler S, von der Schulenburg J-M (2020). A cost-effectiveness analysis model of Preventicus atrial fibrillation screening from the point of view of statutory health insurance in Germany. Heal Econ Rev.

[14] Krivoshei L, Weber S, Burkard T, Maseli A, Brasier N, Kühne M (2017). Smart detection of atrial fibrillation. Europace.

[15] Mutke MR, Brasier N, Raichle C, Ravanelli F, Doerr M, Eckstein J (2020). Comparison and combination of single-lead ECG and photoplethysmography algorithms for wearable-based atrial fibrillation screening. Telemed e-Health.

[16] McManus DD, Chong JW, Soni A, Saczynski JS, Esa N, Napolitano C (2016). PULSE-SMART: pulse-based arrhythmia discrimination using a novel smartphone application. J Cardiovasc Electrophysiol.

[17] O'Sullivan JW, Grigg S, Crawford W, Turakhia MP, Perez M, Ingelsson E (2020). Accuracy of smartphone camera applications for detecting atrial fibrillation: a systematic review and meta-analysis. JAMA Netw Open.

[18] Chan PH, Wong CK, Poh YC, Pun L, Leung WW, Wong YF (2016). Diagnostic performance of a smartphone-based photoplethysmographic application for atrial fibrillation screening in a primary care setting. J Am Heart Assoc.

[19] Jacobs MS, Kaasenbrood F, Postma MJ, van Hulst M, Tieleman RG (2018). Cost-effectiveness of screening for atrial fibrillation in primary care with a handheld, single-lead electrocardiogram device in the Netherlands. Ep Europace.

[20] Pluymaekers N, Dudink E, Luermans J, Meeder JG, Lenderink T, Widdershoven J (2019). Early or delayed cardioversion in recent-onset atrial fibrillation. N Engl J Med.

