# Supplementary material for: Mobile health solutions for atrial fibrillation detection and management: a systematic review
Source: Clin Res Cardiol. 2021 Sep 21;111(5):479–91. doi: 10.1007/s00392-021-01941-9 (PMC8454991; doi:10.1007/s00392-021-01941-9)
Supplement: Supplementary file 1 — Supplementary file1 (PDF 1327 KB) [file 392_2021_1941_MOESM1_ESM.pdf]

Supplementary material online

Table S1. Search strategy.

**Electronic database**	**Search strategy**
PubMed (NCBI)	(((((atrial fibrillation[MeSH Terms]) OR atrial fibrillation[Title]) OR AF[Title]) OR Afib[Title])) AND (((((((((((((((((app) OR application-based) OR app-based) OR smartphone) OR smartphone-based) OR mobile health) OR mhealth) OR ehealth) OR ecardiology) OR telemedicine) OR wearable) OR digital treatment) OR alivecor) OR mydiagnostick) OR fibricheck) OR apple watch) OR myaf) OR remote monitoring)
Embase (Ovid)	(atrial fibrillation or AF or Afib).ti. and (app or app-based or application-based or smartphone-based or smartphone or mobile health or mhealth or ehealth or ecardiology or telemedicine or wearable or digital treatment or alivecor or mydiagnostick or fibricheck or apple watch or myaf or remote monitoring).ab.
Cochrane	(((MeSH descriptor: [Atrial Fibrillation] explode all trees) OR ((atrial fibrillation):ti OR (AF):ti OR (Afib):ti)) AND ((app) OR (app-based) OR (application-based) OR (smartphone) OR (smartphone-based) OR (mobile health) OR (mhealth) OR (ehealth) OR (ecardiology) OR (telemedicine) OR (wearable) OR (digital treatment) OR (alivecor) OR (mydiagnostick) OR (fibricheck) OR (apple watch) OR (myaf) OR (remote monitoring)))

Table S2. Baseline characteristic and outcomes of analyzed studies regrading handheld devices in patients with atrial fibrillation.

Study	Country	Design	Device	Type	Technology	Population	# included patients	Age (years)	Females	Monitoring time	AF rate	Sensitivity	Specificity	Reference test
**PPG-based handheld devices**														
Birkemeyer et al [1]	Germany	Case-control	Smartphone camera	Preventicus	PPG	Simulated group	10000	≥75-years	N/D	N/D	N/D	N/D	N/D	N/D
Brasier et al [2]	Germany Switzerland	Prospective single arm	Smartphone camera	Preventicus	PPG	Hospitalized (cardiology/ pulmonology ward)	592	78±13	45.3%	5min x 1 single-time	41.9%	89.9% (1min) 91.3% (3min) 91.5% (5min)	99.1% (1min) 98.7% (3min) 99.6%(5min)	single-lead ECG
Chan et al [3]	China	Prospective single arm	Smartphone camera	CardioRythm	PPG	With diabetes/ hypertension/elderly (≥65-years old)	1013	68.4±12.2	53.2%	20s x 1 single-time	2.76%	92.9%	97.7%	expert' diagnosis
Fan et al [4]	China	Prospective	Smartphone camera	Preventicus	PPG	Hospitalized (cardiology/ geriatric ward)	108	62 (mean)	41.7%	3 min x 1 single-time	48.1%	95.0%	99.7%	12-lead ECG
Guo et al [5]	China	RCT	Smartphone camera	mAFA	PPG	AF	3324 1646 (IC) 1646 (UC)	67 (mean) 67±15 (IC) 70±12 (UC)	38% 38% (IC) 38% ((UC)	N/D	N/D	N/D	N/D	N/D
Krivoshei et al [6]	Switzerland	Case-control	Smartphone camera	Preventicus	PPG	Ambulatory	80	78 (mean)	N/D	1 min x 1 single-time	50%	90%	85%	single-lead ECG
McManus et al [7]	USA	Prospective single arm	Smartphone camera	PULSE-SMART	PPG	Planned for ECV	121	66 (mean)	19%	2 min x 2 single-time	100%	97%	93.5%	12-lead ECG
Mutke et al [8]	Germany Switzerland	Prospective single arm	Smartphone camera Smartwatch	Preventicus	PPG	Data from DETECT-AF [2] and WATCH-AF[9]	1 101	N/D	N/D	N/D	46.7%	95.4%	91.6%	single-lead ECG
Proesmans et al [10]	Belgium	Prospective single arm	Smartphone camera	FibriCheck	PPG	Elderly (≥65-years old)	223	77±8	53.4%	1 min x 3 single-time	45.7%	95.3%	96.2%	12-lead ECG
Rozen et al [11]	USA	Prospective single arm	Smartphone camera	CardioRythm	PPG	Planned for ECV	98	67.7±10.5	24.5%	20s x 6 single-time	100%	93.1%	90.9%	12-lead ECG
Van Haelst [12]	The Netherlands	Prospective single arm	Smartphone camera	FibriCheck	PPG	Elderly (≥65-years old)	190	77.3±8.0	57.4%	1min x1 single-time	48.4%	98%	88%	12-lead ECG
Verbrugge et al [13]	Belgium	Prospective single arm	Smartphone camera	FibriCheck	PPG	General population	1179	49±14	42%	1min ≥1/day 7 days	1.1%	N/D	N/D	N/D
Yan et al [14]	China	Prospective single arm	Smartphone camera	CardioRythm	PPG	Hospitalized (cardiology ward)	217	70.3±13.9	28.6	20s x 3 single-time	34.6%	95%	96%	12-lead ECG
**ECG-based handheld devices**														
Battipaglia et al [15]	UK	Prospective single arm	Stick	MyDiagnostick	Single-lead ECG	General population	855	N/D	N/D	15s single-time	0.8%	100%*	100%*	Expert expertise
Desteghe et al [16]	Belgium	Prospective two arm	Stick	MyDiagnostick	Single-lead ECG	Hospitalized (cardiology/geriatric ward)	320	67.9±14.6	43.1%,	1 min single-time	11.9% (cardiology ward) 36% (geriatric ward)	60.5% (PM; cardiology ward) 81.8% (no PM; cardiology ward) 81.8% (PM; geriatric ward) 89.5% (no PM; geriatric ward)	93.3% (PM; cardiology ward) 94.2% (no PM; cardiology ward) 96.1% (PM; geriatric ward) 95.7% (no PM; geriatric ward)	12-lead ECG
Jacobs et al [17]	The Netherlands	Prospective two arm	Stick	MyDiagnostick	Single-lead ECG	Influenza-vaccinated	1 952 811	77.4 (mean)	N/D	1min single-time	1.3%	N/D	N/D	N/D

Kaasenbrood et al [18]	The Netherlands	Prospective single arm	Stick	MyDiagnostick	Single-lead ECG	Influenza-vaccinated	3269	69.4±8.9	51%	1 min<br>single-time	3.7%<br>5.9% (detected by device)	96%*	100%*	Expert expertise
Pluymaekers et al [19]	The Netherlands	Multicenter RCT	Stick	MyDiagnostick	Single-lead ECG	Planned for ECV	437	65±11	40%	1 min x 3<br>4 weeks	30% (RAF)	N/D	N/D	N/D
Rivezzi et al [20]	Italy	Prospective single arm	Stick	MyDiagnostick	Single-lead ECG	Elderly (≥65-years old)	1820	50% aged 65-74<br>50% aged ≥75	53.4%	1 min<br>single-time	5.5%<br>7.9% (detected by device0	94%*	100%*	12-lead ECG
Tavernier et al [21]	Belgium	Prospective single arm	Stick	MyDiagnostick	Single-lead ECG	Hospitalized (geriatric ward)	214	84±6	61.7%	1 min<br>single-time	33%	88%	97%	12-lead ECG
Tieleman et al [22]	The Netherlands	Prospective single arm	Stick	MyDiagnostick	Single-lead ECG	Influenza-vaccinated	192	69.4±12.6	51.6%	1 min<br>single-time	27.6%	100%	95.9%	12-lead ECG
Vaes et al [23]	Belgium	Prospective single arm	Stick	MyDiagnostick	Single-lead ECG	Primary care patients (84% with AF)	191	74.6±9.7	48%	1-2 min x 3<br>single-time	54%	94%	93%	12-lead ECG
Anderson et al [24]	USA	Prospective single arm	Plate	KardiaMobile	Single-lead ECG	General population (health fair)	697	56±15	71%	30s x 1<br>single-time	2.3% (16/697)	N/D	N/D	N/D
Andrade et al [25]	Canada	Prospective single arm	Plate	KardiaMobile	Single-lead ECG	Elderly (≥65-years old) without prior AF	16 817	N/D	N/D	30s x 1<br>single-time	7% (1171/16817 detected by device0	69%*	N/D	12-lead ECG
Bumgarner et al [26]	USA	Prospective single arm	Plate attached to watch	KardiaMobile	Single-lead ECG	Planned for ECV	100	68.2±10.9	17%	30s x 1<br>single-time	N/D	93%	84%	12-lead ECG
Caceres et al [27]	USA	RCT	Plate	KardiaMobile	Single-lead ECG	Planned for ECV/AF-ablation	238<br>115 (IC)<br>123 (UC)	61.3±11.9<br>61.4±11.9 (IC)<br>61.2±11.8 (UC)	27%<br>27% (IC)<br>27% (UC)	30s 3x/week<br>6 months	55%<br>61% (IC)<br>49% (UC)	N/D	N/D	N/D
Chan et al [3]	China	Prospective single arm	Plate	KardiaMobile	Single-lead ECG	Elderly (≥65-years old)/ diabetic/ hypertensive	1013	68.4±12.2	53.2%	30s x 1<br>single-time	2.78% (28/1013)	71.4%	99.4%	Expert expertise
Chan et al [28]	China	Prospective single arm	Plate	KardiaMobile	Single-lead ECG	Elderly (≥65-years old) diabetic/ hypertensive	2052	67.8±10.6	54.2%	30s x 1<br>single-time	1.2% (24/2052)	66.7%	99.5%	12-lead ECG
Chan et al [29]	China	Prospective two arm	Plate	KardiaMobile	Single-lead ECG	General population	13122	64.7±13.4	71.5%	30s x 1<br>single-time	1.8% (239/13122 detected by device)	98%	29.2%	Expert expertise
Chan et al [30]	China	Prospective single arm	Plate	KardiaMobile	Single-lead ECG	Elderly (≥50-years old)	10735	78.6±8.1	79.8%	30s x 1<br>single-time	2.3% (244/10735)<br>2.6% detected by device (282/10753)	75%	98.2%	Expert expertise
Chen et al [31]	China	Multicenter prospective single arm	Plate	KardiaMobile	Single-lead ECG	Elderly (≥65-years old)	4531	71.6±6.3	66%	30s x 1<br>single-time	4.0% (183/4531)	N/D	N/D	N/D
Cunha et al [32]	Portugal	Multicenter longitudinal	Plate	KardiaMobile	Single-lead ECG	General population (health fair)	205	66±15	64 %	30s x 1<br>single-time	22% (22/100)<br>22% (22/100 detected by device)	90.9%	97.4%	12-lead ECG
Desteghe et al [16]	Belgium	Prospective two arm	Plate	KardiaMobile	Single-lead ECG	Hospitalized (cardiological or geriatric ward)	320	67.9±14.6	43.1%,	30s x 1<br>single-time	11.9% (cardiology ward)<br>36% (geriatric ward)	36.8% (PM; cardiology ward)<br>54.5% (no PM; cardiology ward)<br>72.7% (PM; geriatric ward)<br>78.9% (no PM; geriatric ward)	96.1% (PM; cardiology ward)<br>97.5% (no PM; cardiology ward)<br>98.1% (PM; geriatric ward)<br>97.9% (no PM; geriatric ward)	12-lead ECG
Evans et al [33]	Kenya	Prospective single arm	Plate	KardiaMobile	Single-lead ECG	Hospitalized (internal, diabetic, ophthalmology, emergency, inpatient men's ward)	50	54.3±20.5	66%	30s x 1<br>single-time	8% (4/50)	100%	100%	12-lead ECG

Godin et al [34]	Canada	Prospective single arm	Plate	KardiaMobile	Single-lead ECG	Elderly (≥65-years old) without prior AF	7585	35% aged ≥65	N/D	30s x 1 single-time	3.9%% (297/7585)<br>6.2% (471/7585 detected by device)	63%	N/D	Expert expertise
Goldenthal et al [35]	USA	RCT	Plate	KardiaMobile	Single-lead ECG	Planned for ECV/AF-ablation	238<br>115 (IC)<br>123 (UC)	61±12<br>61±12 (IC)<br>61±12 (UC)	23%<br>23% (IC)<br>22% (UC)	30s 1/day<br>6 months	50% (IC)<br>42% (UC)	N/D	N/D	N/D
Gropler et al [36]	Singapore	Prospective single arm	Plate	KardiaMobile	Single-lead ECG	Children	30	8.2 (0-17)	53%	30s x 1 single-time	0%<br>13% (4/30 detected by device)	N/D	87%	12-lead ECG
Grubb et al [37]	UK	Prospective single arm	Plate	KardiaMobile	Single-lead ECG	Elderly (≥65-years old) primary healthcare	1805	74.9±7.1	38.9%	30s x 1 single-time	5.1% (92/1805)	N/D	N/D	N/D
Gwynn et al [38]	Australia	Multicenter cross-sectional	Plate	KardiaMobile	Single-lead ECG	Mid-aged (≥45-years old)	619	N/D	56.1%	30s x 1 single-time	4.7% (29/619)<br>4.8% (30/619 detected by device0	85%*	65%*	12-lead ECG
Haberman et al [39]	USA	Prospective single arm	Plate	KardiaMobile	Single-lead ECG	Athletes/young adults/ hospitalized (cardiology ward)	381	35±20	51%	30s x 1 single-time	N/D	94.4%	99.4%	12-lead ECG
Halcox et al [40]	UK	RCT	Plate	KardiaMobile	Single-lead ECG	Elderly (≥65-years old) High stroke risk Without prior AF	1001<br>500 (IC)<br>501 (UC)	72.6±5.4<br>72.6±5.4 (IC)<br>72.6±5.4 (UC)	53.4%<br>51.8% (IC)<br>55.1% (UC)	30s, 2x/week<br>12 months	3.8% (19/500)<br>1.0% (5/501)	N/D	N/D	N/D
Hermans et al [41]	The Netherlands	Prospective single arm	Plate	KardiaMobile	Single-lead ECG	Post AF-ablation	115	64.0 (58.0-68.0)	30.4%	30s, 3x/day in case of symptoms, 4 weeks	25.2% (29/115)	95.3%	97.5%	≥24-h Holter
Hickey et al [42]	USA	RCT	Plate	KardiaMobile	Single-lead ECG	Cardiac electrophysiology and ambulatory care	46<br>23 (IC)<br>23 (UC)	55±10 (IC)<br>55±9 (UC)	29% (IC)<br>29% (UC)	30s >1/day<br>(max 5 min/day)<br>6 months	61% (14/23; IC)<br>30% (7/23; UC)	N/D	N/D	N/D
Kim et al [43]	Korea	Multicenter prospective single arm	Plate	KardiaMobile	Single-lead ECG	Elderly (≥60-years old)	2422	75.5±6.5	68.5%	40s x 1 single-time	3%<br>(73/2422)<br>5.1% (124/2422 detected by device)	59%	N/D	12-lead ECG
Koltowski [44]	Poland	Prospective single arm	Plate	KardiaMobile	Single-lead ECG	Hospitalized (cardiology ward)	100	68±14.2	34%	30s x 1 single-time	N/D	92.8%	100%	12-lead ECG
Kropp et al [45]	USA	Prospective single arm	Plate	KardiaMobile	Single-lead ECG	High AF risk; pharmacy customers	250	61.7±15.3	60%	30s x 1 single-time	3%<br>4% (10/250 detected by device)	N/D	N/D	N/D
Lown et al [46]	USA	Case-control	Plate	KardiaMobile	Single-lead ECG	Elderly (>65-years old) primary healthcare	418	73.9±6.1	N/D	30s x 1 single-time	19% (79/418 detected by device)	87.8%	98.81%	12-lead ECG
Lowres et al [47]	Australia	Prospective single arm	Plate	KardiaMobile	Single-lead ECG	Hospitalized (surgery)	42	69±9	20%	30s, 4x/day<br>4 weeks	24% (10/42 detected by device)	94.6%	92.9%	12-lead ECG
Lowres et al [48]	Australia	Prospective single arm	Plate	KardiaMobile	Single-lead ECG	Elderly (≥65-years old) pharmacy customers	1 000	76±7	56%	30-60s single-time	6.7% (67/1000)	98.5%	91.4%	12-lead ECG
Macniven et al [49]	Australia	Prospective single arm (on device's feasibility)	Plate	KardiaMobile	Single-lead ECG	Primary healthcare	18	N/D	N/D	N/D	N/D	N/D	N/D	N/D
Magnani et al [50]	USA	Prospective single arm	Plate	KardiaMobile	Single-lead ECG	AF	31	68±11	39%	30s, 20 times over 30 days	N/D	N/D	N/D	N/D
Mutke et al [8]	Switzerland	Prospective single arm	Plate	KardiaMobile	Single-lead ECG	Data from DETECT-AF [2] and WATCH-AF[9]	1 101	N/D	N/D	30s x 1 single-time	46.7%	99.6%	97.4%	Expert expertise
Orchard et al [51]	Australia	Prospective single arm	Plate	KardiaMobile	Single-lead ECG	Elderly (≥65-years old) without prior AF	1805	75.7	66%	30s x 1 single-time	3.7% (67/1805 detected by device)	N/D	N/D	N/D
Orchard et al [52]	Australia	Prospective single arm	Plate	KardiaMobile	Single-lead ECG	Elderly (>65-years old) primary healthcare	88	74.8±8.8	49%	30s x 1 single-time	19% (17/88)	N/D	N/D	N/D
Orchard et al [53]	Australia	RCT	Plate	KardiaMobile	Single-lead ECG	Elderly (≥65-years old) without prior AF	3103	75.1±6.8	36%	30s x 1 single-time	1.2% (36/3103)	97%	92%	12-lead ECG

						primary health care								
Orchard et al [54]	Australia	Cross-sectional	Plate	KardiaMobile	Single-lead ECG	Elderly (≥65-years old) influenza-vaccinated	972	N/D	N/D	30s x 1 single-time	3.7% (36/972)<br>4.5% (44/972 detected by device)	95%	99%	Expert expertise
Rajakariar et al [55]	Australia	Prospective single arm	Plate attached to watch	KardiaMobile	Single-lead ECG	Hospitalized (medical, cardiac ward or ICU)	200	67±16	43.5%	30s x 1 single-time	19% (38/200)	94.4%	81.9%	12-lead ECG
Reed et al [56]	UK	RCT	Plate	KardiaMobile	Single-lead ECG	Hospitalized (emergency ward)	243<br>126 (IC)<br>117 (UC)	39.6±13.8<br>40±14 (IC)<br>39.1±13.5 (UC)	66.6%<br>59.2% (IC)<br>53.8% (UC)	30s x 1-2<br>90 days	6.5% (8/124)<br>0 (0/116)	N/D	N/D	N/D
Reed et al [57]	UK	Prospective single arm	Plate	KardiaMobile	Singe-lead ECG	Hospitalized (emergency ward)	68	45.8±15.1	441%	30s x 1-2<br>90 days	2 (3%)	N/D	N/D	N/D
Rischard et al [58]	France	Multicenter prospective single arm	Plate	KardiaMobile	Single-lead ECG	Hospitalized (cardiology ward)	1322	68.7±16.2	34.7%	30s x 1 single-time	20.8% (275/1322)	95%	86%	12-lead ECG
Rosenfeld et al [59]	USA	Multicenter prospective single arm	Plate	KardiaMobile	Single-lead ECG	High AF risk	772	65.2±15.4	67.5%	60s x 1 single-time	2.2% (17/670 detected by device)	N/D	N/D	N/D
Savickas et al [60]	UK	Prospective single arm	Plate	KardiaMobile	Single-lead ECG	Elderly (≥65-years old) influenza-vaccinated	604	73 (69-78)	57.3%	30s x 1-2 single-time	4.3% (26/6040	92.3%	97.4%	12-lead ECG
Selder et al [61]	The Netherlands	Prospective single arm	Plate	KardiaMobile	Single-lead ECG	Ambulatory	233	58.4±14	48%	30s x 1 single-time	N/D	92%	95%	Expert expertise
Soni et al [62]	India	Prospective single arm	Plate	KardiaMobile	Single-lead ECG	General population	2074	33.7% aged ≥66	52.2%	30s, 2-3x/day,<br>5 days	1.6% (33/2074)<br>4.2% (88/2074 detected by device)	38%	N/D	Expert expertise
Stavrakis et al [63]	USA	RCT	Plate	KardiaMobile	Single-lead ECG	Paroxysmal AF<br>High stroke risk	58<br>29 (IC)<br>29 (IC)	62.5±12.2 (IC)<br>60.9±14.4 (UC)	43%<br>41% (IC)<br>45% (UC)	30s ≥1/day<br>7 days	62% (18/29)	N/D	N.D	N/D
Tarakji et al [64]	USA	Prospective single arm	Plate	KardiaMobile	Single-lead ECG	Planned for AF ablation	55	60±12	22%	30s x 1 single-time	N/D	100%	97%	Expert expertise
Van Haelst et al [12]	The Netherlands	Multicenter prospective single arm	Plate	KardiaMobile	Single-lead ECG	Elderly (>65-years old)	190	77.3±8.0	57.4%	30s x 1 single-time	48.4% (92/190)	98%	85%	12-lead ECG
Wasserlauf et al [65]	USA	Prospective two-arm	Plate attached to watch	KardiaMobile	Single-lead ECG	Paroxysmal AF with ICM	24	72.1±7.2	34.6%	30s x 1 single-time	N/D	100%	83.3%	ICM
Wegner et al [66]	Germany	Prospective single arm	Plate	KardiaMobile	Single-lead ECG	Hospitalized (cardiac ward)	99	64±15	38.4%	30s x 1 single-time	22% (22/99)	70%	69%	12-lead ECG
William et al [67]	USA	Prospective single arm	Plate	KardiaMobile	Single-lead ECG	AF	52	68.1 (42.6-85.6)	32.7%	30s x 1 single-time	N/D	96.6%	94.1%	12-lead ECG
Williams et al [68]	UK	Prospective single arm	Plate	KardiaMobile	Single-lead ECG	AF	95	N/D	N/D	30s x 1 single-time	31% (29/95)	93%	76%	12-lead ECG
Yan et al [69]	Australia and China	Prospective two arm	Plate	KardiaMobile	Single-lead ECG	Post stroke/TIA	1079<br>785 (iECG)<br>294 (Holter +iECG)	66 (55-75)<br>64 (54-75)<br>68 (57-77)	38.6%<br>35.8%<br>45.9%	30s x 1 single-time	8.8% (69/785)<br>8.5% (25/294)	N/D	N/D	N/D
Zado et al [70]	USA	Prospective single arm	Plate	KardiaMobile	Single-lead ECG	Post-AF ablation	99	64±8	16%	30s x 2<br>30 days	16% (16/99)	N/D	N/D	N/D
Zaprutko et al [71]	Poland	Prospective single arm	Plate	KardiaMobile	Single-lead ECG	Elderly (≥65-years old) without prior AF pharmacy customers	525	73.7 ±6.5	68.2%	30s x 1 single-time	2.4% (12/490)<br>3.5% (17/490 detected by device)	100%	98.7%	Expert expertise
Aljuaid et al [72]	USA	Retrospective two-arm	Device	ECG check	Single-lead ECG	Post AF-ablation	90<br>45 (IC)<br>45 (UC)	66 (mean)<br>61.3±7.7 (IC)<br>60.9±7.4 (UC)	40%<br>37.8% (IC)<br>42.2% (UC)	30s, 1x/day<br>100 days	N/D	100%	97%	Expert expertise

Anczykowski et al [73]	Germany	Retrospective single arm	Device	Card Guard	Single-lead ECG	With symptoms of cardiac arrhythmia	790	54±18	60%	32s, ≥1/day 2-4weeks	14% (110/790)	N/D	N/D	N/D
Boriani et al [74]	World	Survey	Device/app	Several devices and applications	ECG and PPG	Healthcare professionals	588	N/D	N/D	N/D	N/D	N/D	N/D	N/D
Busch et al [75]	Germany	Retrospective two arm	Device	Sensor mobile 100	Single-lead ECG	General population	1678	54 (28-65)	N/D	30s, 2x/day 4 weeks	1.3% (21/1678) 2.6% (43/1678 detected by device)	N/D	N/D	12-lead ECG
Chen et al [76]	Taiwan	Prospective single arm	Device	DiGiO2	Single lead-ECG	General population	922	58.1±15	53.8%	15s x 1 single-time	2.4% (22/922)	95.5%	97.7%	Expert expertise
Gudmundsdottir et al [77]	Sweden	RCT	Device	Zenicor	Single lead-ECG	Elderly (75/76-years old)	6868	(75-76)	N/D	30s x 1 single-time<br><br>in case of SR and high AF risk: 30s 4x/day 2 weeks	8.1% (553/6688) known AF 0.5% (29/6315) newly detected AF 4.4% (164/3766) newly detected AF in 2 weeks	N/D	N/D	N/D
Gussak et al [78]	Serbia	Prospective single arm	Device	CardioBip	3 lead ECG	Post AF-ablation	25	51.5±2.8	16%	3x/day, 60 days +2x/day 30 days (at 6 month)	32% (8/25) 88% (22/25 detected by device)	100%*	24%*	12-lead ECG and 24-h Holter
Jaakkola et al [79]	Finland	Case-control	Device	Mechanocardiography	Mechanocardiography	Hospitalized (cardiology/internal ward)	300	74.8 (73.7-75.9)	44%	3min x 1 single-time	N/D	95.3 %	96%	Tele-ECG
Liu et al [80]	China	Prospective two arm	Device	Sensor mobile 100	Single-lead ECG	Post AF-ablation	92	54.4±9.8	78.3%	30s, 1x/day, 90 days	29.3% (27/92) 42.4% (39/92 detected by device)	N/D	N/D	12-lead ECG and 24-h Holter
Olsson et al [81]	Sweden	Retrospective single arm	Device	Zenicor	Single lead-ECG	Post stroke/TIA	370	66±12	47%	30s 2x/day 2 weeks	7.6% (27/356)	N/D	N/D	N/D
Svennberg et al [82]	Sweden	RCT	Device	Zenicor	Single lead-ECG	Elderly (75/76-years old)	7173	(75-76)	N/D	30s 2x/day 2 weeks	12.3% (884//7173) 9.3% (666/7173) known AF 3.0%(218/7173) newly detected AF	N/D	N/D	N/D
Vukajlovic et al [83]	Serbia	Prospective single arm	Device	CardioBip	3 lead ECG	Post AF-ablation	21	50.0±13.8	N/D	≤3/day 60 days	195 (4/21) 90% (19/21 detected by device)	N/D	N/D	12-lead ECG and 24-h Holter

Values are presented as median (interquartile range), (range), mean±standard deviation, or number with percentages. **Abbreviations:** AF, atrial fibrillation; DM, diabetes mellitus; ECG, electrocardiogram; ECV, electrical cardioversion; HT, hypertension; IC, intervention care; UC, usual care ; N/D, no data; PM, pacemaker; PPG, photoplethysmography.

***data counted based on available data**

Table S3. Baseline characteristic and outcomes of analyzed studies regrading wearable devices in patients with atrial fibrillation.

Study	Country	Design	Device	Type	Technology	Population	No. of patients	Age (years)	Females	Monitoring time (day)	AF rate	Sensitivity	Specificity	Reference test
**PPG-based wearables**														
Al-Kaisey et al [84]	Australia	Prospective single arm	Wristwatch	Fitbit Charge HR and Apple Watch Series 3	PPG	Referred for Holter monitoring	32	68±12	38%	24 hours	26 (81%)	N/D	N/D	24-hour Holter
Bonomi et al [85]	The Netherlands	Prospective single arm	Wrist device	CM3 Generation-3	PPG	1. Planned for ECV 2. Referred for Holter monitoring	1. 18 2. 34	1. 73.1±11.6 2. 67.4±12.1	1. 44% 2. 38%	1. 42 hours 2. 855 hours	1. 18 (100%) 2. 34 (100%)	1. 97% 2. 93%	1. 100% 2. 100%	1. Single-lead ECG 2. 24-hour Holter
Chen et al [86]	China	Prospective randomized	Wristband	Amazfit Health Band 1S	1. PPG 2. Single-lead ECG	Hospitalized/ ambulatory	401	N/D	49.1%	1. 3 minutes 2. 60 seconds	150 (37%)	1. 88.00% 2. 87.33%	1. 96.41% 2. 99.20%	12-lead ECG
Conroy et al [87]	USA	Prospective single arm	Earlobe sensor	HeartSensor HRS-07UE	PPG	Planned for ECV	55	N/D	N/D	One time for >4 minutes	N/D	90.9%	90.9%	Single-lead ECG
Corino et al [88]	Italy	Prospective single arm	Wristband	Empatica E4	PPG	Hospitalized	70	N/D	49%	10 minutes	30 (43%)	75.4%	96.3%	N/D
Dörr et al [9]	Switzerland	Two-center prospective single arm	Wristband and wristwatch	Wavelet Health and Gear Fit 2	PPG	Hospitalized	508	76.4±9.5	44.3%	5 minutes	237 (46.7%)	93.7%	98.2%	Expert expertise based on single-lead ECG
Eerikäinen et al [89]	The Netherlands	Prospective single arm	Wrist device	CM3 Generation-3	PPG	1. Planned for ECV 2. Ambulatory	1. 18 2. 16	1. 75±11 2. 65±14	1. 44% 2. 37%	1. 1-hour before and after ECV 2. 24 hours	1. 18 (100%) 2. 4 (25%)	1. 92.3% 2. 71.6%	1. 60.7% 2. 84.9%	1. Single-lead ECG 2. 24-hour Holter
Guo et al [90]	China	Prospective single arm	Wristband and wristwatch	Honor Band 4, Huawei Watch GT and Honor Watch	PPG	Ambulatory	187 912	34.7±11.5	13.3%	60 seconds every 10 minutes for 14 days	227/262 (87%)	N/D	N/D	12-lead ECG and 24-hour Holter
Hochstadt et al [91]	Israel	Prospective single arm	Wristwatch	CardiacSense	PPG	Planned for ECV	20	74.1±8.7	25%	30 minutes before ECV and 10 minutes after ECV	20 (100%)	100%	93.1%	12-lead ECG
Huynh et al [92]	USA	Prospective single arm	Wristwatch	Apple Watch A1554	PPG	Obstructive sleep apnea	20	66±6.5	15%	60 seconds seven times	20 (100%)	N/D	N/D	12-lead ECG
Huynh et al [93]	USA	Prospective single arm	Wristwatch	Apple Watch	PPG	OSA in AF	20	66±6.5	15%	60 seconds every 10 seconds	20 (100%)	N/D	N/D	Telemetry
Inui et al [94]	Japan	Prospective single arm	Wristwatch and wristband	Apple Watch Series 3 and Fitbit Charge HR	PPG	Hospitalized (cardiac surgery)	40	70.9±11.1	32%	Two weeks	20 (50%)	N/D	N/D	12-lead ECG

				Wireless Activity										
Jacobsen et al [95]	Germany	Prospective single arm	Upper armband	Everion®	PPG	AF	102	71.0±11.9	48%	24 hours	48 (47%)	95.2%	92.5%	24-hour Holter
Koshy et al [96]	Australia	Prospective single arm	Wristwatch	Apple Watch and Fitbit	PPG	Hospitalized	102	68±15	35%	1 minute every 15 seconds for 30 minutes	32 (31%)	N/D	N/D	ECG telemetry
Kwon et al [97]	Republic of Korea	Prospective single arm	Fingerband	CardioTrac ker	PPG	Persistent AF; planned for ECV	100	63.8±8.5	19%	15 minutes before and after ECV	100 (100%)	99.0%	94.3%	Single-lead ECG
Nemati et al [98]	USA	Retrospecti ve single arm	Wristwatch	Samsung Simband	PPG	Hospitalized	36	N/D	N/D	(3.5-8.5) minutes	12 (33%)	97%	94%	Single-lead ECG
Perez et al [99]	USA	Prospective single arm	Wristwatch	Apple Watch	PPG	Without AF	419 297	41±13	42%	117 (113-186) days	153/450 (34%)	N/D	N/D	7-day ECG patch
Selder et al [100]	Belgium	Prospective single arm	Wristband	1. Wavelet Health with FibriCheck algorithm 2. KardiaBand	1. PPG 2. Single-lead ECG	Elderly	60	69.6±16.9	68%	Three times 1. 60 seconds 2. 30 seconds	6 (10%)	1. 79% 2. 93%	1. 98% 2. 98%	1. Single lead ECG 2. Expert expertise
Seshadri et al [101]	USA	Prospective single arm	Wristwatch	Apple Watch 4	PPG	Hospitalized (cardiac surgery)	50	61.4±10.4	28%	Six times	25 (50%)	N/D	N/D	6-lead telemetry
Tison et al [102]	USA	Multicenter prospective single arm	Wristwatch	Apple Watch	PPG	1. Ambulatory 2. Planned for ECV	1. 1617 2. 51	1. N/D 2. 66.1±10.7	1. N/D 2. 16%	1. N/D 2. 20 minutes	1. 64 (4%) 2. 51 (100%)	1. 67.7% 2. 98.0%	1. 67.6% 2. 90.2%	12-lead ECG
Zhang et al [103]	China	Prospective single arm	Wristband and wristwatch	Honor Band 4, Huawei Watch GT and Honor Watch	PPG	Ambulatory	361	50 (36-62)	49.3%	60 seconds every 10 minutes for 14 days (band) and 45 seconds (watch)	31 (8.6%)	100%	99%	12-lead ECG
**ECG-based wearables**														
Fukuma et al [104]	Japan	Prospective single arm	Patch	T-shirt with a highly conductive material	Single-lead ECG	Low AF risk	100	52.5±5.4	0%	40 hours a week over 2 months	10 (10%)	N/D	N/D	Expert expertise
Heo et al [105]	USA	Prospective single arm	Patch	iRhythm Zio^{XT}	Single-lead ECG	Diabetic without prior AF	608	70.9±6.7	31.9%	2 weeks, twice	19 (3%)	N/D	N/D	Expert expertise
Lown et al [46]	UK	Multicenter prospective single arm	1. Chest belt 2. Patch	1. Polar-H7 2. Firstbeat Bodyguard 2	Single-lead ECG	Elderly (>65-years old)	418	N/D	N/D	1. 45 seconds 2. 2 minutes	79 (19%)	1. 96.3% 2. 96.3%,	1. 98.2% 2. 98.5%,	12-lead ECG
Reverberi et al [106]	Italy	Prospective single arm	Chest belt	Consumer-grade Bluetooth low-energy HR monitor with RITMIA™ application	Single-lead ECG	Planned for ECV	95	66.2±10.7	21.1%	10 minutes	Pre- ECV, 92%; post-ECV 13%	97%	95.6%	12-lead ECG

Sabar et al [107]	UK	Prospective single arm	Patch	RhythmPad	6-lead ECG	High AF risk	750	(18-97)	51%	10 seconds	66 (10%)	AF detection, 93.4%; AF diagnosis 95.4%	AF detection, 96.8%; AF diagnosis, 98.8%	12-lead ECG
Steinhubl et al [108]	USA	Prospective randomized	Patch	Zio^{XT}	Single-lead ECG	High AF risk	2659	72.4±7.3	38.6%	4 weeks	53 (3.9%) vs 12 (0.9%)	N/D	N/D	Expert expertise
Torfs et al [109]	Belgium	Prospective single arm	Patch	N/D	3-lead ECG	1. Chronic AF 2. Planned for ECV	10	N/D	N/D	1. 24 hours 2. 2/3 hours	N/D	N/D	N/D	24-hour Holter
Turakhia et al [110]	USA	Prospective single arm	Patch	Zio^{XT}	Single-lead ECG	High AF risk	75	69±8.0	0%	2 weeks	4 (5.3%)	N/D	N/D	Expert expertise
Wineinger et al [111]	USA	Retrospecti ve single arm	Patch	Zio^{XT}	Single-lead ECG	Paroxysmal AF	13 293	69.4±11.1	40.3%	2 weeks	1 041 504 paroxysmal episodes >30 seconds	N/D	N/D	N/D
Atarashi et al [112]	Japan	Prospective randomized	Wireless recorder	Cardiophon e^{TM}	Single-lead ECG	Paroxysmal AF/ AFL	123	N/D	21%	30 seconds daily for 4 weeks	95 (77%)	N/D	N/D	Expert expertise
Brunetti et al [113]	Italy	Prospective single arm	Wireless recorder	CardioVox P12	12-lead ECG	Hospitalized (emergency ward)	27 841	N/D	51%	N/D	3 249 (11.67%)	N/D	N/D	Expert expertise
Højager et al. [114]	Denmark	Prospective single arm	Wireless recorder	R.Test Evolution 4	ECG	High stroke risk; diabetes	200	66±0.7	41.5%	Every 2-3s; 5-7 days	21 (10.5%)	N/D	N/D	N/D
Kimura et al [115]	Japan	Prospective single arm	Wireless recorder	Cardiophon e^{TM}	Single-lead ECG	Planned for AF ablation	30	59±9	13%	30 seconds twice daily for 6 months	30 (100%)	N/D	N/D	12-lead ECG and 24-hour Holter
Lin et al [116]	Taiwan	Prospective single arm	Wireless recorder	Medi-Trace 200	3-lead ECG	Hospitalized (cardiac ward, ICU)	30	N/D	N/D	6 minutes	20 (67%)	94.6%	N/D	12-lead ECG
Scalvini et al [117]	Italy	Prospective single arm	Wireless recorder	CG-7100	12-lead ECG	Primary healthcare	7 516	61±20	56%	N/D	719 (9%)	N/D	N/D	N/D
Wu et al [118]	Taiwan	Prospective single arm	Wireless recorder	CG-7100 and CG-2100	12-lead ECG (7100) and single-lead ECG (2100)	Cardiovascular disease	70	N/D	N/D	N/D	25 (36%)	N/D	N/D	N/D
**Pulse variability-based wearables**														
Chan et al [28]	China	Prospective single arm	Sphygmom anometer	Microlife WatchBP Office AFIB	Pulse beat interval algorithm	Diabetic or hypertensive	2052	67.8±10.6	54.2%	N/D	24 (1.2%)	83.3%	98.7%	12-lead ECG
Gandolfo et al [119]	Italy	Prospective single arm	Sphygmom anometer	Microlife BP3MQ1-2D	Pulse beat interval algorithm	Post stroke/TIA	207	77.7±11.34	50.2%	Three times	38 (18.4%)	89.5%	98.8%	12-lead ECG
Kearley et al [120]	UK	Prospective single arm	Sphygmom anometer	WatchBP	Pulse beat interval algorithm	Ambulatory	999	79.7 (75.1-99.8)	50.7%	One time	79 (7.9%)	94.9%	89.7%	12-lead ECG
Marazzi et al [121]	Italy	Prospective single arm	Sphygmom anometer	1. Microlife® BP A200 Plus	Pulse beat interval algorithm	Hypertensive	503	67.0±10.5	45.7%	Three times	101 (20%)	1. 92% 2. 100%	1. 97% 2. 94%	12-lead ECG

				2. OMRON® M6										
Omboni et al [122]	Italy	Prospective single arm	Sphygmomanometer	Microlife WatchBP Office AFIB	Pulse beat interval algorithm	Ambulatory	220	57.5±15.3	48.6%	Three times	4 (1.8%)	N/D	N/D	Single-lead ECG
Stergiou et al [123]	Greece	Prospective single arm	Sphygmomanometer	Microlife BPA 100 Plus	Pulse beat interval algorithm	Hospitalized/ ambulatory/ aged ≥35	73	70.5±10.6	34.2%	Three times	27 (37%)	100%	89%	12-lead ECG
Wiesel et al [124]	USA	Prospective single arm	Sphygmomanometer	Microlife BP monitor model BPM BP3MQ1-2D	Pulse beat interval algorithm	High AF risk	139	67 (26-89)	63%	Three times a day for 30 days	14 (10.1%)	99.2%	92.9%	Single-lead ECG
Wiesel et al [125]	USA	Prospective single arm	Sphygmomanometer	Microlife BP monitor model BP3MQ1-2D	Pulse beat interval algorithm	Ambulatory	405	73 (34-98)	49%	Three times	93 (23%)	96.8%	88.8%	12-lead ECG
Wiesel et al [126]	USA	Prospective single arm	Sphygmomanometer	Omron 712C	Pulse beat interval algorithm	Ambulatory	450	69 (31-99)	41%	Two times	54 (12%)	100%	91%	12-lead ECG

Values are presented as median (interquartile range), (range), mean±standard deviation, or number with percentages. **Abbreviations:** AF, atrial fibrillation; AFL, atrial flutter; ECG, electrocardiogram; ECV, electrical cardioversion; ICU, intensive care unit); N/D, no data; OSA, obstructive sleep apnea; PPG, photoplethysmography.

Table S4. Overview of the implantable cardiac monitor technologies under assessment.

**Device features**	**BioMonitor 2-AF™ (Biotronik SE& Co. KG, Berlin, Germany)**	**Reveal LINQ™ (Medtronic plc, Minneapolis, MN, USA)**	**Reveal XT (Medtronic plc, Minneapolis, MN, USA)**	**Confirm Rx™ (Abbott Laboratories, Lake Bluff, IL, USA)**
Standard components	• BioMonitor 2-AF device with flexible lead body<br>• Insertion tools<br>• SensingConsult™ (Biotronik SE & Co. KG) programmer, software	• Reveal LINQ device<br>• Insertion tools<br>• CareLink™ (Medtronic) programmer, software<br>• MyCareLink™ monitor	• Reveal XT device<br>• Insertion tools<br>• CareLink™ (Medtronic) programmer, software	• Confirm Rx device<br>• Insertion tools<br>• Merlin™ (Abbott) programmer, software<br>• myMerlin™ mobile app
Additional components		• Patient activity accelerometer<br>• Triage and monitoring service (FocusOn™; Medtronic)		• Symptom annotator via app<br>• Free technical support available via helpline or local staff
Patient activation	• Optional patient assistant device	• Patient assistant device as standard	• Optional patient assistant device	• Integrated™ in myMerlin app
Detection triggers and sensing parameters	• Atrial fibrillation (adjustable or pre-set functions to detect various atrial fibrillation characteristics)<br>• Bradycardia<br>• Sudden rate drop<br>• Asystole<br>• High ventricular rate	• Atrial tachyarrhythmia (including atrial fibrillation/ flutter) (exclusive algorithm) P-wave morphology discriminator algorithm<br>• Bradyarrhythmia<br>• Ventricular tachyarrhythmia<br>• Pause episodes	• Atrial tachyarrhythmia (including atrial fibrillation/ flutter) (exclusive algorithm)<br>• Bradyarrhythmia<br>• Ventricular tachyarrhythmia<br>• Pause episodes	• Atrial fibrillation (regularity, R–R variance and sudden onset)<br>• Bradyarrhythmia<br>• Tachyarrhythmia<br>• Pause episodes<br>• Transient loss of consciousness conditions<br>• Epilepsy exclusion
Dimension	88.4 x15.2 x 6.2mm	44.8 x 7.2 x 4.0mm	95 x 62 x 8.0mm	49.0 x 9.4 x 3.1mm
Weight	10.1g	2.5 ± 0.5g	15g	3.0g
Device storage	• total duration of 60 min<br>• 55 x 40s automatically activated episodes<br>• 4 x 7.5 min patient-activated episodes	• total duration of 57 min<br>• 27 min of automatically activated episodes<br>• 30 min of patient-activated episodes	• total duration of 49.5 min<br>• 27 min of automatically activated episodes<br>• 22.5 min of patient-activated episodes	• total duration of 60 min<br>• up to 250 atrial fibrillation episodes plus 250 auto-/patient-activated episodes of other arrhythmias
Estimated battery life	4 years	3 years	3 years	2 years
Telemetry	• via mobile phone network to Home Monitoring Service Centre	• via myCareLink™ monitor to a CareLink™ server using a mobile phone network	• via CareLink™ programmer to CareLink™ server	• via app to Merlin.net patient care network accessed by clinicians
Sensitivity	92%[127] – 100%[128]	97.4%[129] a – 100%*[130] a<br>98.4%[129] b<br>93.7%[129] c<br>97.3%[129] d<br>97.2%[129] e	96.1%[131] a – 96.1%*[130] a<br>98.1%[130] b<br>89.0%[130] c<br>85.2%[130] d<br>87.9%[130] e	100%[132] a<br>84.2%[132] b<br>96.4%[132] c<br>94.5%[132] d<br>95.6%[132] e
Specificity	67%[128]	97.0%[129] a – 99.0%*[130] a<br>99.5%[129] b<br>99.6%[129] c	85.4%[131] a – 90.0%*[130] a<br>98.5%[130] b<br>91.3%[130] c	85.7%[132] a<br>99.4%[132] b<br>99.4%[132] c<br>86.6%[132] d<br>98.2%[132] e
PPV	59% [127] – 83%[128]	92.5%[129] a – 97.4%*[130] a<br>97.2%[129] b<br>90.6%[129] c<br>74.8%[129] d<br>90.4%[129] e	79.3%[131] a – 84.9%*[130] a<br>91.9%[130] b<br>75.2%[130] c<br>38.8%[130] d<br>73.6%[130] e	64%[132] a<br>93.5%[132] b<br>63.3%[132] c<br>64.0%[132] d<br>60.7%[132] e
NPV	100%[128]	99.0%[129] a – 100%*[130] a<br>99.7%[129] b<br>96.4%[129] c	97.4%[131] a – 97.5%*[130] a<br>99.7%[130] b<br>97.1%[130] c	100%[132] a<br>98.4%[132] b<br>92.2%[132] c<br>76.4%[132] d<br>89.8%[132] e
Accuracy	N/A	97.1%[129] a – 99.3%*[130] a	89.3%[131] a – 92.2%*[130] a	N/A

*adaptive P-Sense algorithm. Diagnostic significance in BioMonitor and Confirm devices are reported for their earlier versions. a, patient based (gross); b, duration based (gross); c duration gross (patient average); d, episode based (gross); e episode gross (patient average). **Abbreviations:** PPV, positive predictive value; N/A, non-available; NPV, negative predictive value.

Table S5. Baseline characteristic and outcomes of analyzed studies regrading implantable cardiac monitor in patients with atrial fibrillation.

Study	Country	Design	Device	No. of patients	Age (years)	Females	Population	Monitoring time (day)	Implantation after event (day)	Insertion to AF detection (day)	AF definition	AF rate
Asaithambi et al [133]	USA	Retrospective single arm	Reveal LINQ	234	72 [61-78]	45%	CS	536 [282-848]	4 [2-9]	94.5 [16-239]	N/D	20 (9%) (1mo)<br>47 (20%) (6mo)<br>57 (24%) (12mo)<br>68 (29%) (22mo)
Bergau et al [134]	Germany	Retrospective randomized	Reveal XT	30	67±10	44%	Post-AF ablation	1011±388	at the same day	N/D	≥30s	N/D
Bertelsen et al [135]	Denmark	RCT	Reveal LINQ	68	76.2±4.5	33.8%	Post-MRI	41 (36-43)	N/A	N/D	≥6min	32 (47%)
Carrazco et al [136]	USA	Prospective and retrospective single arm	Reveal LINQ (90%)/ XT (10%)	100	65.8 [28-93]	52.5%	CS	(240-540)	4.2±2.6	108 (0-514)	≥2min	25 (25%)
Choe et al [137]	International	ICM registry vs. simulated intermittent monitoring	Reveal XT	168	61.3± 11	32%	CS	>345	N/D	N/D	>30s	30 (18%)
Chorin et al [138]	USA	Retrospective single arm	Reveal LINQ<br>Reveal XT	145	67 [53-70]	43%	CS	28 ± 12mo	N/D	7.4 ± 21.3 mo	≥2min	4 (2.8%) (1mo)<br>8 (5.5%) (6mo)<br>11 (7.6%) (12mo)<br>13 (9.6%) (24mo)<br>17 (12%) (36mo)
Christensen et al [139]	Denmark	Prospective single arm	Reveal XT	85	56.7 (mean)	45%	CS	569±310	107±117	109±48	>2min	14 (16%)
Ciconte et al [128]	Italy	Prospective single arm	BioMonitor	63	60.4±9.4	13.6%	AF-episodes/ management	N/D	N/D	N/D	>2min	39 (62%)
Cotter et al [140]	UK	Prospective single arm	Reveal XT	51	51±13.9	45.1%	CS	229±116	174±134	48 [34-118]	≥2min	13 (25%)
Dekker et al [141]	International	Prospective single arm	Reveal LINQ	121	57±11.4	25.6%	Pre/post AF-ablation	56 [37-174] (pre-) >180 (post ablation)	N/D	N/D	≥5min	28/71 (39%)
De With [142]	The Netherlands	RCT	Reveal LINQ	202	64±9	42%	Paroxysmal AF	183	N/D	N/D	≥2 min	139 (69%)
Diederichsen et al [143]	International	Prospective single arm	Reveal LINQ	597	76±4.0	43%	High stroke risk	1200 [1110-1260]	N/D	165 [42-510]<br>420 [144-780]<br>600 [192-900]	≥6min<br>≥5h<br>≥24h	209 (35%)
Dion et al [144]	France	Prospective single arm	Reveal Plus	24	49±13.6	37.5%	CS	435 (mean)	90±30.3	N/D	≥30s	1 (4.2%)
Etgen et al [145]	Germany	Prospective single arm	Reveal XT	22	61.6 [51.9-71.3]	50%	CS	360	8.5 [6.5-10.5]	152.8 [61.6-244.1]	≥6min	6 (27%)
Forkmann et al [146]	Germany	Prospective single arm	Reveal LINQ	126	63±10	38.9%	Post AF-ablation	90 (ER)<br>360 (LR)	at the next day	N/D	≥30s	72 (57%) (ER)<br>57 (45%) (LR)
Haldar et al [147]	International	RCT	Reveal LINQ	120	62.3±9.6	36%	Post AF-ablation	22 (16-31) mo	0	N/D	≥30s	83 (73%)
Healey et al [148]	Canada	Prospective single arm	Confirm-AF	256	74±6	34.4%	High stroke risk	489±114	N/D	153±165	≥5min	31 (12.1% (1mo)<br>58 (22.3%) (6mo)<br>78 (30.5%) (12mo)<br>90 (35.2%) (18mo)
Hindricks et al [131]	International	Prospective single arm	Reveal XT	247	57±10	33.2%	High AF risk	N/D	N/D	N/D	≥2min	76 (37%)
Israel et al [149]	Germany	Prospective single arm	Reveal XT (87%)/ BioMonitor (13%)	123	65±9.0	39.8%	ESUS	381±165	20 (mean)	108±102	≥2min	8 (6.5%) (1mo)<br>15 (12%) (3mo)<br>21 (17%) (6mo)

												29 (24%) (13mo)
Jorfida et al [150]	Italy	Prospective single arm	Reveal XT	54	67.8±9.4	42.6%	CS	435 (261-675)	108±60	162 [30-540]	≥5min	25 (46%)
Kitsiou et al [151]	Germany	Prospective single arm	Reveal XT	123	65±9	40%	ESUS	1095	20 (mean)	354 (mean)	≥2min	51 (41.4%)
Kusiak et al [152]	Poland	Prospective single arm	Reveal LINQ	29	55.6 (34-72)	31%	Post AF-ablation	90 (before) 180 (after)	N/D	N/D	≥2min	N/D
Lacour et al [153]	Germany	Retrospective single arm	BioMonitor 2-AF	19	61.3±13.0	42.1%	CS, HCM, SSS, syncope, AT, VT	42	N/D	N/D	N/D	1 (5.3%)
Lauschke et al [127]	International	Prospective single arm	BioMonitor	153	62.5±14.0	49%	Post AF-ablation	321±108	at the same day	N/D	≥2min	3/77 (3.9%)
Makimoto et al [154]	Germany	Prospective single arm	N/D	146	62±12	42.5%	ESUS	387 [283-552]	N/D	N/D	>30s	30 (21%)
Marks et al [155]	USA	Retrospective single arm	Reveal XT /LINQ	178	65 (mean)	52%	CS	384.1 ±218.9	5 (mean)	131.5 (median)	>2min	9 (5%) (1mo)<br>14 (16.2%) (12mo)<br>35 (19.6%) (30mo)
Merce et al [156]	Spain	Prospective single arm	Reveal XT	14	65.4±10.9	28.6%	CS	870±177	<30	174 (mean)	N/D	5 (36%)
Muller et al [157]	Germany	Prospective single arm	Reveal XT	90	57.7±12.3	48%	CS	331±186	<60	40.7±42.2	≥30s	8 (9%) (1mo)<br>16 (18%) (6mo)
Nasir et al [158]	USA	Prospective single arm	Reveal LINQ(?)/ XT(?)	245	74.3±7.7	41.2%	High stroke risk	451±185	<30	141.3±139.5	≥6min	54 (22%)
Nölker et al [132]	International	Prospective single arm	Confirm DM2102	90	65.7± 9.6	39%	High AF/AF risk	N/D	N/D	N/D	≥2min	N/D
Pedersen et al [159]	Denmark	Prospective single arm	Reveal LINQ (72%)/ XT (28%)	105	65.4 [27.1-80.8]	54%	TIA	381 [371-390]	113 (30-294)	21 (5-146)	≥2min	7 (6.7%)
Poli et al [160]	Germany	Prospective single arm	Reveal LINQ (51%)/ XT (49%)	74	66.4±12.5	53.3%	CS	311±251	27±24	105±135	>2min	25 (34%)
Prabhu et al [161]	Australia	RCT	Confirm<br>Reveal LINQ	68	(65-74)	9%	Post AF-ablation/medical rate control	6 months	At the procedure	N/D	>30s	average AF burden post-AF ablation was 1.6±5.0%
Purerfellner et al [130]	International	Enhanced AF algorithm detection using XPECT [131] and LINQ Usability Study [129]	Reveal LINQ (40%)/ XT (60%)	346	N/D	N/A	Post AF-ablation<br>AF-management<br>Syncope, CS<br>high AF risk	N/D	N/D	N/D	≥2min	114 (33%)
Reiffel et al [162]	International	Prospective single arm	Reveal LINQ (69%)/XT(31%)	394	71.5±9.9	47.8%	High stroke risk	675±231	N/D	123 [41-330]	≥6min	6.2% (1mo)<br>20.4% (6mo)<br>27.1% (12mo)<br>29.3% (18mo)<br>33.6% (24mo)<br>40% (30mo)
Reinke et al [163]	Germany	Prospective single arm	Reveal XT	105	64.4±12.6	44%	CS	217[72.5-338]	0-28	N/D	≥30s	19 (18%)
Ritter et al [164]	Germany	Within-patient comparison of 7-day ECG vs. ICM	Reveal XT	60	63 [48.5-72]	43%	CS	382 [89-670]	13 [10-67]	64 (1-556)	≥30s	4 (6.7%) (1mo)<br>7 (12%) (3mo)<br>9 (15%) (6mo)<br>9 (15%) (12mo)<br>10 (17%) (21mo)
Romanov et al [165]	International	Prospective randomized	Reveal XT	60	62.5±6.5	20%	Post CABG	1080	at the same day	N/D	N/D	22 (37%)
Sanders et al [129]	International	Prospective single arm	Reveal LINQ	138	56.6±12.1	33%	Post AF-ablation<br>AF-management<br>Syncope, CS<br>high AF risk	30	N/D	N/D	≥2min	38 (28%)

Sanna et al [166]	International	Prospective randomized	Reveal XT	441 (221with ICM)	61.5±11.3	36.5%	CS	1080	38.1±27.6	41 [14-84](6mo)<br>84 (18-265) (12mo)	≥30s	(8.9%) (6mo)<br>(12.4%) (12mo)<br>(30%) (36mo)
Seow et al [167]	Singapore	Prospective single arm	Reveal LINQ	71	61.9±13.5	22.5%	CS	345±229	66 (median)	50 (median)	≥2min	9 (12.9%) (6mo)<br>11 (15.2%) (12mo)
Victor et al [168]	Spain	Prospective single arm	Reveal LINQ (86%)/XT (14%)	65	65.4±13.8	44.6%	ESUS	513±321	56 [28-109]	31 [11-59]<br>28 [20.5-117](XT)<br>31 [11-56] (LINQ)	≥30s (XT)<br>≥2min (LINQ)	19 (29%)
Wasserlauf et al [169]	USA	Within-patient comparison of AF-sensing watch vs. ICM	Reveal LINQ	24	72.1±7.2	34.6%	Syncope, AF-management, CS	110.3±35.7	N/D	N/D	N/D	N/D
Watson et al. [170]	USA	Retrospective single arm	Reveal LINQ	273	64.8±13.4	49.5%	CS<br>CRAO	5.1 (max; CS) 3.6 (max; CRAO)	18 (median; CRAO) 21 (median; CS)	N/D	≥2min	64 (23%)
Wechselberger et al [171]	International	Prospective single arm	Reveal LINQ	419	65±10	43%	Post AF-ablation	450±180	at the same day	N/D	≥2min	227 (54%)
Xu et al. [172]	USA	Prospective single arm	Reveal LINQ	389	67 (mean)	46%	CS	542 days (median)	N/D	133 days (mean)	≥2min	102 (27.2%)
Yaeger et al [173]	USA	Unclear<br>(ICM group within PENN AF Care Program)	Reveal XT	251	N/D	N/D	Post AF-ablation	30	at the same day	N/D	≥30s	90 (36%)
Ziegler et al [174, 175]	USA	ICM registry vs. simulated intermittent monitoring	Reveal LINQ	1 247	65.3±13.0	47%	CS	579±222	N/D	112 [35-293]	≥2min	4.6% (1mo)<br>12.2% (6mo)<br>16.3% (12mo)<br>21.5% (24mo)

Values are presented as median (interquartile range), (range), mean±standard deviation, or number with percentages. **Abbreviations:** AF, atrial fibrillation; AT, atrial tachyarrhythmia; CABG, coronary artery bypass graft; CS, cryptogenic stroke; ECG, electrocardiogram; ER, early recurrences; ESUS, embolic stroke of undetermined source; HCM, hypertrophic cardiomyopathy; ICM; implantable cardiac monitor; LR, late recurrences; mo; month; N/D, no data; SC, subcutaneous implantation; SP, subpectoral implantation; SSS, sick sinus syndrome; VT, ventricular tachyarrhythmia.

Table S6. Available FDA/CE-approved mobile applications.

Name	Company	FDA/ CE	Method, technology	Clinical application (selected)		Sensitivity	Specificity	PPV
				AF detection	Other			
**Devices and mobile applications that are SPECIFIED in AF detection**								
ECG App	Apple	FDA	smartwatch, PPG	yes	no	98,3%	99,6%	N/A
ECG Check	Cardiac Designs, Inc.	FDA, CE	ECG-handle device, ECG	yes	no	75%	97%	N/A
FibriCheck	Qompium nv	FDA, CE	smartphone's camera, PPG	yes	no	97%	94%	61.3%
Kardia	AliveCor, Inc.	FDA, CE	handle device, ECG	yes	no	89%	89%	89%
Microlife Connected Health	Microlife Corp.	CE	arm-worn device, MCG	yes	blood pressure	81-100%	89-99%	N/A
MoMe Kardia	InfoBionic, Inc.	FDA, CE	handle device, ECG	yes	no	N/A	N/A	N/A
myMerlin for Confirm Rx ICM	Abbott (formerly St. Jude Medical)	FDA, CE	implantable cardiac monitor	yes	no	97%	N/A	N/A
PatientCare; BodyGuardian Heart	Preventice, Inc.	FDA, CE	wearable patch ECG (chest)	yes	no	95.9%	N/A	99%
Peerbridge Cor	Peerbridge Health, Inc.	FDA	wearable patch ECG (chest)	yes	no	N/A	N/A	N/A
Verily Study Watch	Verily (Google Alphabet)	FDA	Smartwatch, PPG	yes	no	N/A	N/A	N/A
Eko Stethoscope CORE/DUO	Eko Devices, Inc.	FDA, CE	electronic stethoscope, MCG	yes	auscultation	99%	97%	NA

**Devices and mobile applications that COULD be used in AF detection (ECG feature)**				
**Name**	**Company**	**FDA/ CE**	**Method, technology**	**Clinical application (selected)**
Health Mate	Withings France SA (formerly Nokia)	FDA, CE	arm-device, watch/mobile application	ECG, blood pressure, skin temperature, activity tracking, sleep tracking, respiratory tracking, women health
Global ECG Management System (GEMS)	CardioComm Solutions, Inc.	FDA, CE	handle ECG device	ECG
Qardio heart health	Qardio, Inc.	FDA, CE	arm-device, chest belt with sensors/mobile application	ECG, blood pressure, heart rate, respiratory rate, activity tracking, body composition, women health
SimplECG	Nanowear, Inc.	FDA	bra or undershirt with sensors	ECG, blood pressure, heart rate, respiratory rate
Master Caution	HealthWatch Technologies, Ltd.	FDA, CE	vest with sensors	ECG, heart rate, respiratory rate, skin temperature, body posture
McKesson ECG Mobile	McKesson Corp.	FDA	wearable patch ECG (chest)	ECG
Invision ECG system	InvisionHeart, Inc.	FDA	12-Lead ECG device	ECG
Physiotrace Smart	NimbleHearth, Inc.	FDA	wearable patch ECG (chest)	ECG
Rooti Rx	Rooti Labs, Ltd.	FDA	wearable patch ECG (chest)	ECG
Smartheart	Shl Telemedicine, Ltd.	FDA, CE	ECG put-on chest device	ECG
CoVa™ 2	toSense, Inc.	FDA	neck-worn sensor	ECG, stroke volume, cardiac output, chest fluid, heart rate, heart rate variability, respiration, skin temperature; remote monitoring portal access

CADence IRONMAN	AUM Cardiovascular, Inc.	FDA	electronic stethoscope	auscultation (heart, lung), ECG, blood pressure
**No matching applications in AF detection used in "Heart/Circulatory System"**				
Viz.ai	Viz.ai, Inc.	FDA, CE	mobile application	automatically identify suspected large vessel occlusion strokes on computed tomography angiogram imaging
RhythmAnalytics	Biofourmis, Inc	FDA	arm-worn device	heart rate, skin temperature, respiratory rate, blood oxygenation, blood pulse wave, fitness trackers, sleep quality, heart rate variability, inter-beat-interval & a stress score
StethoMe	StethoMe sp. z o.o.	CE	electronic stethoscope	auscultation (heart, lung), heart rate, respiration rate
CareTaker	CareTaker, LLC	FDA, CE	hand-cuff device	blood, pressure, heart rate, respiration rate, oxygen saturation, arterial stiffness
eMurmur ID	CSD Labs International Inc. d.b.a. eMurmur	FDA, CE	electronic stethoscope	auscultation (heart, lung), heart rate, respiration rate, health assistance (video-chat)
PhysioWave Pro	PhysioWave, Inc.	FDA	stand-on device	pulse wave velocity, pulse rate, body weight
Syndo Health	Syndo Health nv	CE	mobile application	blood pressure, fitness and body mass trackers, medication alerts, health educational tips, health assistance (chat)
Samsung Health	Samsung Electronics Co, Ltd.	FDA	mobile application	heart rate, oxygen saturation, stress, women health, fitness and body trackers
iHealth MyVitals	iHealthLabs, Inc.	FDA, CE	mobile application	blood pressure, scales, pulse oximeters, fitness and body mass trackers.
Masimo Professional or Personal Health	Masimo Corp.	FDA, CE	mobile application	heart rate, oxygen saturation, respiratory rate, respiratory effort index, pleth variability index, perfusion index

Abbreviations: CE, Conformité Européenne; ECG, electrocardiogram; FDA, Food & Drug Administration; MCG, mechanocardiography; N/A, non-applicable; PPG, photoplethysmography; PPV, positive predictive value.

Table S7. Mobile applications and platforms supporting management of atrial fibrillation.

Study	Country	Design	Intervention	No. of patients	Age (years)	Females	%of AF patients	Study duration	Outcomes measured	Results
**For patients**										
Desteghe et al [176]	USA	Prospective single arm	Mobile app = Health Buddies<br>- medication tracker, educational quizzes (grandparent)<br>- healthy challenge tracker, educational games (grandchild)<br>- communication with HCP<br>- OAC refilling reminders	15 + grandchildren (n=20)	69.2 ±3.7	33%	100%	3 months	Patient AF knowledge (JAKQ)<br>Patient drug adherence (MMAS-8)<br>Patient motivation to use<br>Mobile app feasibility, usability, satisfaction	• Patient AF knowledge: improved from 64.6+14.7% at baseline to 70.4+10.4% after 3 months (p = 0.09)<br>• Medication adherence: 7.7±0.6 at baseline and 7.4±0.9 at end of study; electronic monitoring showed lower taking and regimen adherence than self-reported on app (taking adherence 88.6±15.4%) and regime adherence (81.8±18.7%); pill count adherence 94.5±9.2%<br>• Motivation to use app: decreased towards end of study in both pts (p= 0.009) and grandchildren (p < 0.001). 87% of patients completed the 90-day contract<br>• Mean days using app significantly higher in pts vs grandchildren (57.7±30.0% and 24.3±23.8%, respectively; p= 0.002)<br>• App experience: rated positively on clarity (1.500), novelty (0.942) and stimulation (0.923) and attractiveness (0.859); efficiency (0.577) and dependability (0.481) received neutral rating
Magnani et al [50]	USA	Prospective single arm	Mobile app<br>(animated character with speech, body gesture, facial expression)<br>- AF education<br>- symptom tracker<br>- medication adherence<br>- heart rate/rhythm monitoring (by ACK)	31	68±11	39%	100%	1 month	Patient QoL (AFEQT, HRQoL)<br>Patient medication adherence (MMAS-8)<br>Patient activation (PAM)	• Patient QoL: improved from 64.5±22.9 at baseline to 76.3±19.4 (p<0.01)<br>• Patient drug adherence: improved from 7.3±0.9 to 7.7±0.5 (p=0.01)<br>• Most of the participants found the relational agent useful, informative, and trustworthy
Ghanbari et al [177]	USA	Prospective single arm	Mobile app = miAfib<br>- AF symptom tracker<br>- affect tracker	10	ND	50%	100%	1 month	Patient engagement and acceptability<br>Mobile app usability (5-ponit Likert scale)	• Patients found app easy to use (4.75 ± 0.46), intended to use it in the future (4.37 ± 1.06) and found it easy to integrate into daily routine (4.5 ± 1.07)
Guo et al [178]	China	RCT	Mobile application = mAFA<br>- AF education<br>- CDSS (CHA_2DS_2-VASc, HAS-BLED, SAMe-TT_2R_2 scores)<br>- thromboprophylaxis guidance<br>- patient event tracker<br>- heart rhythm monitoring<br>- blood pressure monitoring<br>- symptom tracker<br>- lifestyle trackers<br>- medication adherence<br>- self-care protocols<br>- structured follow-up	3292<br>1646 (IC)<br>1646 (UC)	67 (mean)<br>67±15 (IC)<br>70±12 (UC)	38%<br>38% (IC)<br>38% (IC)	100%	291 days (mean)	Assessment of mobile application impact on long term outcomes	• Composite of ischemic stroke/systemic thromboembolism, death, and rehospitalization (1.9% in IC vs 6.0% in UC); HR 0.39 (95% CI 0.22-0.67); p<0.01<br>• Rehospitalization rate (1.2% in IC vs. 4.5% in UC); HR 0.32 (95% CI 0.17- 0.60); p<0.001
Guo et al [179]	China	RCT		1793<br>657 (IC)<br>1136 (UC)	64±24<br>N/D (IC)<br>N/D (UC)	33%<br>N/D (IC)<br>N/D (UC)	100%	12 months	Assessment of mobile application impact on long term outcomes	• Bleeding events (2.1% in IC vs 4.3% in UC, p<0.01)<br>• OAC use decreased significantly by 25% among AF patients receiving UC
Guo et al [180]	China	RCT		209<br>113 (IC)<br>96 (UC)	67.4 ±10.6<br>70.9 ±17.4	42.5%<br>44.8%	100%	1 and 3 months	Patient AF knowledge (11-item AF questionnaire)<br>Patient QoL (EQ-5D-Y)<br>Patient drug adherence (Pharmacy Quality Alliance adherence measure)<br>Patient OAC satisfaction (Anti-Clot Treatment Scale)	• Patient AF knowledge: improved vs. standard care (all p<0.05)<br>• Patient QoL: increased vs. standard care at baseline (86.5 vs. 71.3), 1 month (87.6 vs. 70.1) and 3 months (87.2 vs. 69.9) (all p<0.05)<br>• Patient drug adherence: increased vs. standard care at 1 month (0 (0–4) vs. 4 (0–11); p<0.001); 3 months (2 (0–4) vs.4 (0–11); p<0.001)

									Mobile app feasibility, usability, satisfaction	• Patient anticoagulation satisfaction: standard care expressed more OAC burden (all p< 0.05); mAFA pts reported significantly more OAC benefit at 1 month only (p=0.013)<br>• App usability: 90% reported app
Guo et al [181]	China	RCT		2473<br>1261 (IC)<br>1212 (UC)	67.0 (mean) (IC)<br>70.1 (mean) (UC)	38.0% (IC)<br>42.1% (UC)	100%	≥1 year	Stroke/thromboembolism<br>All-cause death<br>Rehospitalization<br>App usability	• Lower composite outcome of ischaemic stroke/systemic thromboembolism, death, and rehospitalization in IC vs UC (HR 0.18, 95% CI: 0.13–0.25, *p*< 0.001)<br>• App usability: 842 patients used mAFA, of which 70.8% had good management adherence, with the persistence of use of 91.7%
Hirschey et al [182]	USA	Prospective single arm	Mobile app<br>- AF education<br>- AF episodes tracker<br>- AF triggers tracker<br>-news from Cardiology Societies<br>- medication reminders<br>- heart rate monitoring<br>- appointment reminder	12	59<br>[37-67]	42%	100%	1 month	Mobile app feasibility, usability, satisfaction	• App satisfaction (reported by 92% of participants), ease of use (100%), software bugs (58%), similarity to other apps used before (83%)<br>• Perceptions of app usefulness: core needs of the patient segment, patient workflow while managing AF, app's ability to support the patient's evolving needs<br>• Usability improvement: clarity of app instructions and design, software bugs
Manimaran et al [183]	UK	Prospective single arm	Mobile app = Ortus-iHealth (virtual arrhythmia clinic appointment via video call)	46	62<br>[23-86]	36%	100%	3 months	Mobile app feasibility, usability, satisfaction	• Patient activation: high satisfaction with installation and registration process (in 62% patients), sense of reminders (100%) and clinical letters (83%) usefulness, sense of cost- and time-effectiveness (80%)
Stephan et al [184]	Brazil	Prospective single arm	Mobile application<br>- AF education<br>- CDSS (CHA_2DS_2-VASc, HAS-BLED, SAMe-TT_2R_2 scores)	20	67.7<br>±9.4	40%	100%	N/D	Patient AF knowledge<br>Treatment decision conflict	• Patient AF knowledge: increased from 4.7±1.8 to 7.2±1.0, p<0.001<br>• Mobile app used resulted in low decisional conflict (11 ± 16/100 points in decisional conflict scale)
Balsam et al [185]	Poland	Prospective single arm	Educational program<br>- AF education (video)	100	63±15	38%	62%	1 year	Patient AF knowledge (stroke risk, stroke reduction by drugs/OAC)	• Patient AF risk knowledge: increased from 22% (before), to 83% (immediately after), 79% (1 week after), 71% (1 year after movie)<br>• Patient drugs knowledge: increased from 88% (before) to 100% (immediately after), 100% (1 week after), 97% (1 year after)<br>• Patient OAC knowledge: increased from 70% (before) to 96% (immediately after), 92% (1 week after), 99% (1 year after)
Desteghe et al [186]	Belgium	RCT	Educational program<br>- AF education (platform)<br><br>1. on-line tailored education group (group 1)<br>2. standard care with online access (group 2)<br>3. standard care only (group 3)	120<br>35 (gr.1)<br>36 (gr.2)<br>49 (gr.3)	68±10.2<br>62±10.1 (gr.1)<br>65±8.9 (gr.2)<br>74±7.6 (gr.3)	35%<br>23% (gr.1)<br>31% (gr.2)<br>47% (gr.3)	100%	12 months	Patient AF knowledge (JAKQ)<br>Patient QoL (AFEQT)<br>Patient experience/ opinion	• Group 1: significantly improved AF knowledge (75.0 [66.7–85.0] %; p= 0.001); knowledge persisted at 6 weeks (77.5 [65.0–85.0]%; p=0.010) and 12 weeks (80.0 [70.0–90.0] %; p< 0.001) after DCCV/PVI procedure<br>• Group 2: no improvement in AF knowledge (65.0 [50.0–73.8]%; p=1.00); significant improvement between baseline and 6-week post-procedure (p= 0.010) and between hospitalization and 6-week post-procedure (p= 0.016)<br>• Group 3: no knowledge improvement (p =0.248)<br>• Group 1 and 2: significant increase in overall AFEQT score 6- and 12-week post-procedure compared to baseline and at hospitalization<br>• Group 3: no significant difference in overall AFEQT score over time (p=0.082)<br>• Usability: on-line platform rated positively on all aspects

Goni et al [187]	Spain	RCT	Educational program for Mediterranean diet enriched with extra virgin olive oil<br>- phone contacts<br>- web-based interventions with provision of dietary recommendations<br>- access to web page, mobile app and printed resources	720<br>365 (IC)<br>355 (UC)	59.7±10 .7<br>59.9±10 .5 (IC)<br>59.6±10 .9 (UC)	171 (24%)<br>82 (22.5%) (IC)<br>89 (25.1%) (UC)	N/D	2 years	14-item Mediterranean Diet Adherence Screener (MEDAS) questionnaire and a semi quantitative food frequency questionnaire	• Adherence to Mediterranean diet: higher improvement in IC compared with UC (net between-group difference: 1.8 points in the MEDAS questionnaire; p<.001)
Guhl et al [188]	USA	RCT	Educational program (animated character with speech, body gesture, facial expression)<br>- AF education<br>- AF symptom tracker<br>- heart rhythm monitoring (by ACK)	120<br>61 (IC)<br>59 (UC)	72±9.1<br>72+10.6 (IC)<br>73+7.3 (UC)	52%<br>53% (IC)<br>51% (UC)	100%	1 month	Patient QoL (AFEQT)<br>Patient experience/ opinion<br>Patient daily activity<br>Patient medication adherence	• Patient QoL: improved in IC (adjusted mean difference 4.5; 95% CI 0.6-8.3; p=0.03) compared with the UC<br>• Patient daily activity: improved in IC (adjusted mean difference 7.1; 95% CI 1.8-12.4; p=0.009) compared with the UC<br>• Patient medication adherence (3.5% in IC vs. 23.2% in UC; adjusted difference 16.6%; 95% CI 2.8%-30.4%; p<0.001).<br>• Qualitative assessments of acceptability identified that participants found the relational agent useful, informative, and trustworthy
Carter et al [189]	Canada	RCT	Educational program<br>- AF education via telephone	433<br>185 (UC)<br>228 (IC)	64±15<br>64±15 (IC)<br>64±15 (UC)	44%<br>43% (IC)<br>45% (UC)	100%	12 months	Assessment of AF knowledge on long term outcomes and OAC prescription	• Composite of death, cardiovascular hospitalization, and AF-related emergency department visits: 17.3% vs. 26.2% (IC vs. UC) OR 0.71 (95% CI 0.59 - 1.00); p=0.049<br>• Prescription of OAC increased in the $CHADS_2$ ≥2 group (88.4% in the IC vs. 58.5% in UC group, *p*<0.01).
Ferguson et al [190]	Australi a	Prospective single arm	Educational program<br>- thromboprophylaxis guidance and AF education based on 12 case scenarios	74	88% aged<45	82%	N/D	6 weeks	Nurse AF and OAC knowledge<br>Mobile app satisfaction	• There was a 54% mean improvement in knowledge levels post-intervention<br>• Improvement in the use of the CHA_2DS_2-VASc (2.5–37.5%) and HAS-BLED (2.5–35%) tools to assess stroke and bleeding risk (*p*<0.01)<br>• Mobile app satisfaction: very high satisfaction with the learning module (87%), with the content clarity (89%), ease in use (87%)
Mesquita et al [191]	Internati onal	RCT	Educational program<br>- online training for electrophysiologists regarding identification of AF driver sites	12<br>6 (IC)<br>6 (UC)	30 [28-32]<br>28 [26-30] (IC)<br>31 [30-33] (UC)	50%<br>50% (IC)<br>50% (UC)	100%	N/D	Impact of online training on identification of AF driver sites	• Baseline identification of AF termination sites increased from 35%±8% to 50.0%±8.2% (p=0.04) in training group, whereas no changes were observed in control group (37%±10% to 37.8%±10.5%, p=NS)<br>• Training improved overall performance by 13.1%±3.6%, (p<0.001) while accuracy did not change for first- and second reads in the control group (2.7%±3.4%, p=0.439)
Cox et al [192]	Canada	RCT	CDSS<br>- AF education<br>- patient health record<br>- thromboprophylaxis guidance<br>- heart rate and rhythm control guidance<br>- symptom tracker	11333<br>590 (IC)<br>543 (UC)	72+10<br>73+10 (IC)<br>72+9.9 (UC)	39.1%<br>60% (IC)<br>65% (UC)	100%	12 months	Assessment of CDS impact on long term outcomes	• Composite of unplanned emergency department visit or cardiovascular hospitalization 20.0% vs. 23.9% (IC vs. UC) HR 1.06 (95% CI 0.77 - 1.47); p=0.71<br>• Major bleeding 1.3% vs. 1.3% (IC vs. UC) HR 1.04 (95% CI 0.38 - 2.88); p=0.94
Eckman et al. [193]	USA	Prospective single arm	CDSS<br>- thromboprophylaxis guidance	65	66±10.5	35%	100%	1 month	Clinical decision support<br>Patient AF knowledge<br>Patient AF knowledge about personal stroke and bleeding risk	• Decisional conflict decreased from an average of 31 to 9; mean change was 22.3 (95% CI, 25.7 - 37.1)<br>• Satisfaction with decision increased from 4.0 to 4.5<br>• Patient AF knowledge increased from 8.4 to 9.1

									Patient medication adherence (MMAS-8)	• Patient AF knowledge bout personal stroke and bleeding risk increased from 1 to 1.5 ($p < 0.0001$)<br>• Patient medication adherence improved from 5.9 to 6.4 ($p<0.0001$).
Eckman et al. [194]	UK<br>USA	RCT	CDSS<br>- thromboprophylaxis guidance	1493<br>801 (IC)<br>692 (UC)	70<br>70 (IC)<br>70 (UC)	44%<br>44%<br>48%	100%	12 months	Clinical decision support	• Rate of discordant therapy decreased from 63% to 59% (p=0.02).
Hendriks et al [195]	The Netherlands	RCT	CDSS<br>- AF management (thromboprophylaxis guidance, related treatment)	712<br>356 (IC)<br>356 (UC)	67±12<br>66±13 (IC)<br>67±12 (UC)	41.3%<br>44.7% (IC)<br>37.9% (UC)	100%	22 months (mean)	Comparison of AF nurse-led and routine clinical care	• Composite of cardiovascular hospitalization or cardiovascular death 14.3% vs. 20.8% (IC vs.UC) HR 0.65 (95% CI 0.45_0.93); p=0.017
Karlsson et al [196]	Sweden	RCT	CDSS<br>- alert about high-risk patients left untreated	13379<br>7764 (IC)<br>6370 (UC)	59% of aged ≥75<br>58% (IC)<br>58% (UC)	43%<br>43% (IC)<br>43% (UC)	100%	12 months	Assessment of CDS impact on long term outcomes and guidelines adherence	• Adherence to guidelines increased from 70.3% (95% CI 62.9%–77.7%) to 73.0%, (95% CI 64.6%–81.4%) in IC and from 70.0% (95% CI 60.4%–79.6%) to 71.2% (95% CI 60.8%–81.6%) in UC, p = 0.013<br>• No difference in the incidence of stroke, transient ischemic attack, or systemic thromboembolism in the IC vs. UC (49 [95% CI 43–55] per 1,000 patients with AF in the IC compared to 47 [95% CI 39–55] per 1,000 patients with AF in UC, p= 0.64)<br>• IC had a lower incidence of significant bleeding, with events in 12 (95% CI 9–15) per 1,000 patients with AF compared to 16 (95% CI 12–20) per 1,000 patients with AF in UC (p = 0.04)
Sheibani et al [197]	Iran	Prospective single arm	CDSS<br>- thromboprophylaxis guidance	10 HCP<br>373 pts	43.8<br>[33-58]	30%	100% (newly diagnosed)	6 months before and 6 months after intervention	Provider adherence to OAC guidelines for AF	• CDSS improved adherence to guidelines for OAC for AF (from 48% to 65.5%, p < 0.0001)
Thomson et al [198] (DARTS-II)	UK	RCT	CDSS<br>- thromboprophylaxis guidance	109<br>53 (IC)<br>56 (UC)	73<br>73±6.7 (IC)<br>74±6.2 (UC)	44%<br>43% (IC)<br>45% (UC)	100%	3 months	Clinical decision support	• Decision conflict was lower in IC vs. UC; mean difference -0.18 (95% CI -0.34 to -0.01)<br>• Participants in IC not already on warfarin were much less likely to start warfarin than those in UC (25% vs. 93.8%); RR 0.27 (95% CI 0.11-0.63).
Wijtvielt et al [199]	The Netherlands	RCT	CDSS<br>- AF management (thromboprophylaxis guidance, related treatment)	1374<br>671 (IC)<br>683 (UC)	64±10<br>64±10 (IC)<br>64±11 (UC)	44%<br>33% (IC)<br>35% (UC)	100%	37 months	Comparison of AF nurse-led and routine clinical care	• Composite of cardiovascular death and cardiovascular hospital admissions 9.7% per year vs. 11.6% per year (IC vs. UC) HR 0.85 (95% CI 0.69-1.04); p=0.12<br>• In a pre-specified subgroup analysis by center experience; HR 0.52 (95% CI 0.37–to 0.71) in four experienced centers and 1.24 (95% CI 0.94–1.63) in four less experienced centers (p for interaction <0.001).
Rosier et al [200]	France	Retrospective single arm	AF detection system<br>- application filtering AF alerts	60	N/D	N/D	100%	9 months (mean)	Adequate classification of remote AF alerts	• 98% adequate classification of AF alerts<br>• 84% reduction in the workload of remote monitoring of AF alerts
Zoppo et al [201]	Italy	Prospective single arm	AF detection system (AFinder web-based software)	472	69±10	23%	44%	24 months	AF detection via web-based software scanning of CIED data remote transmissions	• AFinder enhanced AF detection sensitivity by 10%<br>• AFinder improved OAC optimal treatment by a factor of 6%
Shacham et al [202]	Israel	RCT	Remote patient-management system<br>(remote management of PAF by telephonically transmitted recommendations (group A)	646<br>576 (gr.A)<br>160 (gr. B)	73±11	48%	100%	2-3 months	Remote AF management	• Group A: 79.5% of PAF episodes (1326/1667) were converted into sinus rhythm; mean heart rate decreased from 85±15 to 66±10 beats per minute (p<0.001).<br>• Group B: 70% of PAF episodes (153/218) were converted into sinus rhythm; mean heart rate decreased from 92 ± 24 to 68 ± 21 beats per minute (p<0.001).

			or also included intervention by the attending physician (group B))							
Stegman et al [203]	Germany	RCT	Remote patient management (daily transmission of body weight, blood pressure, heart rate/rhythm, oxygen saturation, and self-rated health status)	571<br>282 (IC)<br>289 (UC)	74+8.0 (IC)<br>74+8.1 (UC)	32% (IC)<br>30% (UC)	100%	12 months	Remote AF management on long term outcomes	• Days lost due to unplanned cardiovascular hospital admissions or all-cause death: 5.64%,(95% CI 3.81-7.48) in IC vs. 9.37% (95% CI 6.98-11.76) in UC group, ratio 0.60, P = 0.015).<br>• All-cause mortality: 9.2% (95% CI 6.1–13.2) IC vs. 14.5% (95% CI 10.7–18.1) in the UC group (HR 0.60, CI 0.36–1.00, p= 0.050).
Jiang et al [204]	China	Prospective single arm	Remote patient-management system = HCFT-AF<br>- AF education (for patient, HCP)<br>- personal health record<br>- AF symptom tracker<br>- heart rate/rhythm monitoring<br>- blood pressure monitoring<br>- remote consultation	73	68±10.3	48%	100%	4 months	HCFT-AF program acceptability, feasibility, and usability	• HCFT-AF program satisfaction 5.21+1.43; ease of use 4.76+1.58, usefulness 5.45+1.40; overall usability 5.11+1.52<br>• Self-monitoring of blood pressure increased from 26% to 72% (p<0.001), heart rate from 8% to 52%(p<0.001), heart rhythm from 7% to 48%(p<0.001)<br>• Healthy lifestyle: moderate physical activity increased from 22% to 42% (p=0.09), quitting or reducing alcohol intake from 51 to 73% (p=0.005), quitting or reducing smoking from 62% to 72% (p=0.04)<br>• Low-salt, low-fat diet increased from 42% to 61% (p=0.04), more fruits or vegetables consumption increased from 25% to 76% (p<0.001)<br>• 94% of indicated patients received OAC therapy
Goette et al [205]	Germany	RCT	Remote patient-management system=transtelephonic ECG transmission	225<br>214 (IC)<br>211 (UC)	61+11.2 (IC)<br>62+10.2 (UC)	38% (IC)<br>45% (UC)	100%	12 months	AF burden	•
Stewart et al [206]	Australia	RCT	Remote patient-management system<br>- home visit and Holter monitoring 7-14 days after hospital discharge with additional telephone support	335<br>127 (IC)<br>137 (UC)	72±11<br>72±11 (IC)<br>71±12 (UC)	48%<br>50% (IC)<br>46% (UC)	100%	905 [773-1050] days	Comparison of AF nurse-led and routine clinical care	• Death or unplanned readmission 76% vs. 82% (IC vs. UC) HR 0.97 (95% CI 0.76 - 1.23); p=0.85
Vinereanu et al [207]	International	RCT	Remote patient-management system<br>- AF education with regular monitoring and feedback to HCP	2281<br>1187 (IC)<br>1094 (UC)	70±11<br>70±11 (IC)<br>69±12 (UC)	47.3%<br>48% (IC)<br>46% (UC)	100%	12 months	Change in the proportion of patients treated with OAC Reduction in stroke risk	• Change in proportion of patients on OAC: from 68% to 80% (IC) from 64% to 67% (UC)<br>• Absolute difference in the change between groups was 9.1% (95% CI 3.8-14.4); OR of change in the use of OAC between groups was 3.28 (95% CI 1.67-6.44; adjusted p value=0.0002).<br>• Kaplan-Meier estimates showed a reduction in the stroke in the IC vs. UC (HR 0.48, 95% CI 0.23-0.99; log-rank p value=0·0434).
Peleg et al [208]	Italy	Prospective single arm	Remote patient-management system<br>- AF education<br>- patient health record<br>- symptom tracking<br>- lifestyle tracking<br>- CDSS<br>- medication adherence<br>- heart rate/rhythm monitoring<br>- blood pressure monitoring	10	N/D	N/D	100%	127.2 ±68.6 days	Patient compliance to ECG and blood pressure measurement<br>Patient QoL (EuroQoL and AFEQT)<br>Clinician / patient compliance to DSS recommendations<br>Patient satisfaction (Likert scale)<br>Clinician satisfaction (Likert scale)	• Patient compliance to ECG and blood pressure measurement was 0.65±0.28 and 0.75±1.33, respectively<br>• The proportion of AF episodes in patient-initiated measurements was higher than that found in system-initiated requests (p=0.01)<br>• Patient QoL: increased from 77.6 ± 0.23 to 78.4 ± 0.23; 50% patients improved, 18% remained, 37.5% deteriorated (EuroQoL, utility coefficient), from 67.5 ± 18.6 to 80.1 ± 13.0; 62.5% patients improved, 12.5% remained, 25% deteriorated (EuroQoL, analogue score) from 73.0 ± 14.9 to 67.8 ± 11.1; 25% patients improved, 12.5% remained, 62.5% deteriorated AFEQT,

										(overall score), from 72.3±18.7 to 71.9±21.7; 37.5% patients improved, 37.5% remained; 25% deteriorated (AFEQT, treatment satisfaction)<br>• Clinician compliance to DSS recommendations (0.3)<br>• Patient compliance to DSS recommendations (>0.9)<br>• Patient satisfaction: system increased patient's confidence (in 50% of patients), made ability to adapt to context (86%), improved patients' peace of mind during travel (88%), improved their interaction with clinicians (>50%); was recommend to others (100%), was intended to use it in the future (89%); not complicated patients' lives (33%)<br>• Clinician satisfaction: system helped to identify priorities and increases patient safety (in 100% of clinicians), made it easier to manage patients (100%)

Abbreviations: AF, atrial fibrillation; AFDST, Atrial fibrillation Decision Support tool; AFEQT, Atrial Fibrillation Effect on Quality of life; AFSDM, Atrial fibrillation shared decision-making tool; AI, artificial intelligence; CDS, clinical decision support; CDS-AF, Clinical Decision Support for Atrial Fibrillation; CDSS, computerized decision support system; DARTS, Decision Analysis in Routine Treatment Study; ECG, electrocardiogram; IC, intervention care; INR, international Normalized Ratio; MMAS-8, Morisky 8-item Medication Adherence Scale; N/OAC, oral anticoagulant; PAM, Patient Activation Measure; RCT, randomized controlled trial; UC, usual care.

Table S8. Excluded studies and reason for exclusion.

Author, year, reference	Reason for exclusion
Lau 2013 [209]	Wrong intervention (development optimized algorithm for AF detection)
Lee 2013 [210]	Wrong intervention (development new AF detection technology)
McManus 2013 [211]	Wrong intervention (development new AF detection technology)
Shrivastav 2014 [212]	Wrong intervention (not focused only on AF population)
Chung 2015 [213]	Wrong intervention (development new QTc interval detection technology)
Marcolino 2015 [214]	Wrong intervention (description of teleservice collecting 12-lead ECG)
Nguyen 2015 [215]	Wrong intervention (not focused only on AF population)
Weidemann 2016 [216]	Wrong intervention (not focused only on AF population)
Coppetti 2017 [217]	Wrong intervention (not focused only on AF population)
Chong 2018 [218]	Wrong intervention (development new AF detection technology)
Lahdenoja 2018 [219]	Wrong intervention (development new AF detection technology)
Nguyen 2018 [220]	Wrong intervention (development new AF detection technology)
Baca-Motes 2019 [221]	Wrong intervention (methods for recruitment potential research participants)
Marinucci 2020 [222]	Wrong intervention (development new AF detection technology)
Luo 2021 [223]	Wrong intervention (development new AF detection technology)

Abbreviations: AF, atrial fibrillation; ECG, electrocardiogram.

References

[1] Birkemeyer R, Müller A, Wahler S, von der Schulenburg J-M. A cost-effectiveness analysis model of Preventicus atrial fibrillation screening from the point of view of statutory health insurance in Germany. Health Economics Review. 2020;10:1-18.

[2] Brasier N, Raichle CJ, Dörr M, Becke A, Nohturfft V, Weber S, et al. Detection of atrial fibrillation with a smartphone camera: first prospective, international, two-centre, clinical validation study (DETECT AF PRO). Ep Europace. 2019;21:41-7.

[3] Chan PH, Wong CK, Poh YC, Pun L, Leung WWC, Wong YF, et al. Diagnostic performance of a smartphone-based photoplethysmographic application for atrial fibrillation screening in a primary care setting. Journal of the American Heart Association. 2016;5:e003428.

[4] Fan Y-Y, Li Y-G, Li J, Cheng W-K, Shan Z-L, Wang Y-T, et al. Diagnostic performance of a smart device with photoplethysmography technology for atrial fibrillation detection: pilot study (Pre-mAFA II registry). JMIR mHealth and uHealth. 2019;7:e11437.

[5] Guo Y, Lane DA, Wang L, Zhang H, Wang H, Zhang W, et al. Mobile health technology to improve care for patients with atrial fibrillation. Journal of the American College of Cardiology. 2020;75:1523-34.

[6] Krivoshei L, Weber S, Burkard T, Maseli A, Brasier N, Kühne M, et al. Smart detection of atrial fibrillation. Europace. 2017;19:753-7.

[7] McManus DD, Chong JW, Soni A, Saczynski JS, Esa N, Napolitano C, et al. PULSE-SMART: pulse-based arrhythmia discrimination using a novel smartphone application. Journal of cardiovascular electrophysiology. 2016;27:51-7.

[8] Mutke MR, Brasier N, Raichle C, Ravanelli F, Doerr M, Eckstein J. Comparison and Combination of Single-Lead ECG and Photoplethysmography Algorithms for Wearable-Based Atrial Fibrillation Screening. Telemedicine and e-Health. 2020.

[9] Dörr M, Nohturfft V, Brasier N, Bosshard E, Djurdjevic A, Gross S, et al. The WATCH AF Trial: SmartWATCHes for Detection of Atrial Fibrillation. JACC Clin Electrophysiol. 2019;5:199-208.

[10] Proesmans T, Mortelmans C, Van Haelst R, Verbrugge F, Vandervoort P, Vaes B. Mobile Phone–Based Use of the Photoplethysmography Technique to Detect Atrial Fibrillation in Primary Care: Diagnostic Accuracy Study of the FibriCheck App. JMIR mHealth and uHealth. 2019;7:e12284.

[11] Rozen G, Vaid J, Hosseini SM, Kaadan MI, Rafael A, Roka A, et al. Diagnostic accuracy of a novel mobile phone application for the detection and monitoring of atrial fibrillation. The American journal of cardiology. 2018;121:1187-91.

[12] Van Haelst R. The diagnostic accuracy of smartphone applications to detect atrial fibrillation: a head-to-head comparison between Fibricheck and AliveCor. ACTA CARDIOLOGICA: TAYLOR & FRANCIS LTD 2-4 PARK SQUARE, MILTON PARK, ABINGDON OR14 4RN, OXON, ENGLAND: TAYLOR & FRANCIS LTD; 2017. p. 584-.

[13] Verbrugge FH, Proesmans T, Vijgen J, Mullens W, Rivero-Ayerza M, Van Herendael H, et al. Atrial fibrillation screening with photo-plethysmography through a smartphone camera. EP Europace. 2019;21:1167-75.

[14] Yan BP, Lai WH, Chan CK, Chan SCH, Chan LH, Lam KM, et al. Contact-free screening of atrial fibrillation by a smartphone using facial pulsatile photoplethysmographic signals. Journal of the American Heart Association. 2018;7:e008585.

[15] Battipaglia I, Gilbert K, Hogarth AJ, Tayebjee MH. Screening for atrial fibrillation in the community using a novel ECG recorder. Journal of atrial fibrillation. 2016;9.

[16] Desteghe L, Raymaekers Z, Lutin M, Vijgen J, Dilling-Boer D, Koopman P, et al. Performance of handheld electrocardiogram devices to detect atrial fibrillation in a cardiology and geriatric ward setting. Ep Europace. 2017;19:29-39.

[17] Jacobs MS, Kaasenbrood F, Postma MJ, van Hulst M, Tieleman RG. Cost-effectiveness of screening for atrial fibrillation in primary care with a handheld, single-lead electrocardiogram device in the Netherlands. Ep Europace. 2018;20:12-8.

[18] Kaasenbrood F, Hollander M, Rutten FH, Gerhards LJ, Hoes AW, Tieleman RG. Yield of screening for atrial fibrillation in primary care with a hand-held, single-lead electrocardiogram device during influenza vaccination. Ep Europace. 2016;18:1514-20.

[19] Pluymaekers NA, Dudink EA, Luermans JG, Meeder JG, Lenderink T, Widdershoven J, et al. Early or delayed cardioversion in recent-onset atrial fibrillation. New England Journal of Medicine. 2019;380:1499-508.

[20] Rivezzi F, Vio R, Bilato C, Pagliani L, Pasquetto G, Saccà S, et al. Screening of unknown atrial fibrillation through handheld device in the elderly. Journal of Geriatric Cardiology: JGC. 2020;17:495-501.

[21] Tavernier R, Wolf M, Kataria V, Phlips T, Huys R, Taghji P, et al. Screening for atrial fibrillation in hospitalised geriatric patients. Heart. 2018;104:588-93.

[22] Tieleman R, Plantinga Y, Rinkes D, Bartels G, Posma J, Cator R, et al. Validation and clinical use of a novel diagnostic device for screening of atrial fibrillation. Europace. 2014;16:1291-5.

[23] Vaes B, Stalpaert S, Tavernier K, Thaels B, Lapeire D, Mullens W, et al. The diagnostic accuracy of the MyDiagnostick to detect atrial fibrillation in primary care. BMC family practice. 2014;15:1-7.

[24] Anderson JR, Hunter T, Dinallo JM, Glaser D, Roybal LK, Segovia A, et al. Population screening for atrial fibrillation by student pharmacists at health fairs. Journal of the American Pharmacists Association. 2020.

[25] Andrade JG, Godin R, Nault I. Large-scale implementation of a pragmatic atrial fibrillation screening program in canadian community practice. Pacing and Clinical Electrophysiology. 2020.

[26] Bumgarner JM, Lambert CT, Hussein AA, Cantillon DJ, Baranowski B, Wolski K, et al. Smartwatch algorithm for automated detection of atrial fibrillation. Journal of the American College of Cardiology. 2018;71:2381-8.

[27] Caceres BA, Hickey KT, Bakken SB, Biviano AB, Garan H, Goldenthal IL, et al. Mobile electrocardiogram monitoring and health-related quality of life in patients with atrial fibrillation: findings from the iPhone Helping Evaluate Atrial Fibrillation Rhythm Through Technology (iHEART) study. Journal of Cardiovascular Nursing. 2020;35:327-36.

[28] Chan P-H, Wong C-K, Pun L, Wong Y-F, Wong MM-Y, Chu DW-S, et al. Head-to-head comparison of the AliveCor heart monitor and Microlife WatchBP Office AFIB for atrial fibrillation screening in a primary care setting. Circulation. 2017;135:110-2.

[29] Chan N-y, Choy C-c. Screening for atrial fibrillation in 13 122 Hong Kong citizens with smartphone electrocardiogram. Heart. 2017;103:24-31.

[30] Chan N-Y, Choy C-C, Chan C-K, Siu C-W. Effectiveness of a nongovernmental organization–led large-scale community atrial fibrillation screening program using the smartphone electrocardiogram: An observational cohort study. Heart Rhythm. 2018;15:1306-11.

[31] Chen Y, Huang Q-F, Sheng C-S, Zhang W, Shao S, Wang D, et al. Detection rate and treatment gap for atrial fibrillation identified through screening in community health centers in China (AF-CATCH): A prospective multicenter study. PLoS medicine. 2020;17:e1003146.

[32] Cunha S, Antunes E, Antoniou S, Tiago S, Relvas R, Fernandez-Llimós F, et al. Raising awareness and early detection of atrial fibrillation, an experience resorting to mobile technology centred on informed individuals. Research in Social and Administrative Pharmacy. 2020;16:787-92.

[33] Evans GF, Shirk A, Muturi P, Soliman EZ. Feasibility of using mobile ECG recording technology to detect atrial fibrillation in low-resource settings. Global heart. 2017;12:285-9.

[34] Godin R, Yeung C, Baranchuk A, Guerra P, Healey JS. Screening for atrial fibrillation using a mobile, single-lead electrocardiogram in Canadian primary care clinics. Canadian Journal of Cardiology. 2019;35:840-5.

[35] Goldenthal IL, Sciacca RR, Riga T, Bakken S, Baumeister M, Biviano AB, et al. Recurrent atrial fibrillation/flutter detection after ablation or cardioversion using the AliveCor KardiaMobile device: iHEART results. Journal of Cardiovascular Electrophysiology. 2019;30:2220-8.

[36] Gropler MR, Dalal AS, Van Hare GF, Silva JNA. Can smartphone wireless ECGs be used to accurately assess ECG intervals in pediatrics? A comparison of mobile health monitoring to standard 12-lead ECG. PLoS One. 2018;13:e0204403.

[37] Grubb NR, Elder D, Broadhurst P, Reoch A, Tassie E, Neilson A. Atrial fibrillation case finding in over 65 s with cardiovascular risk factors–Results of initial Scottish clinical experience. International journal of cardiology. 2019;288:94-9.

[38] Gwynn J, Gwynne K, Rodrigues R, Thompson S, Bolton G, Dimitropoulos Y, et al. Atrial fibrillation in Indigenous Australians: a multisite screening study using a single-lead ECG device in Aboriginal primary health settings. Heart, Lung and Circulation. 2020.

[39] Haberman ZC, Jahn RT, Bose R, Tun H, Shinbane JS, Doshi RN, et al. Wireless smartphone ECG enables large-scale screening in diverse populations. Journal of cardiovascular electrophysiology. 2015;26:520-6.

[40] Halcox JP, Wareham K, Cardew A, Gilmore M, Barry JP, Phillips C, et al. Assessment of remote heart rhythm sampling using the AliveCor heart monitor to screen for atrial fibrillation: the REHEARSE-AF study. Circulation. 2017;136:1784-94.

[41] Hermans ANL, Gawalko M, Pluymaekers N, Dinh T, Weijs B, van Mourik MJW, et al. Long-term intermittent versus short continuous heart rhythm monitoring for the detection of atrial fibrillation recurrences after catheter ablation. Int J Cardiol. 2021.

[42] Hickey KT, Biviano AB, Garan H, Sciacca RR, Riga T, Warren K, et al. Evaluating the utility of mHealth ECG heart monitoring for the detection and management of atrial fibrillation in clinical practice. Journal of Atrial Fibrillation. 2017;9.

[43] Kim NR, Choi CK, Kim H-S, Oh S-H, Yang J-H, Lee KH, et al. Screening for Atrial Fibrillation Using a Smartphone-Based Electrocardiogram in Korean Elderly. Chonnam Medical Journal. 2020;56:50-4.

[44] Koltowski L, Balsam P, Glłowczynska R, Rokicki JK, Peller M, Maksym J, et al. Kardia Mobile applicability in clinical practice: A comparison of Kardia Mobile and standard 12-lead electrocardiogram records in 100 consecutive patients of a tertiary cardiovascular care center. Cardiology Journal. 2019.

[45] Kropp CM, Huber NL, Sager D, Tripp C, Burch A, Naniwadekar A, et al. Mobile-ECG screening in rural pharmacies: rates of atrial fibrillation and associated risk factors. Heart & Lung. 2020.

[46] Lown M, Yue AM, Shah BN, Corbett SJ, Lewith G, Stuart B, et al. Screening for Atrial Fibrillation Using Economical and Accurate Technology (From the SAFETY Study). Am J Cardiol. 2018;122:1339-44.

[47] Lowres N, Mulcahy G, Gallagher R, Ben Freedman S, Marshman D, Kirkness A, et al. Self-monitoring for atrial fibrillation recurrence in the discharge period post-cardiac surgery using an iPhone electrocardiogram. European Journal of Cardio-Thoracic Surgery. 2016;50:44-51.

[48] Lowres N, Neubeck L, Salkeld G, Krass I, McLachlan AJ, Redfern J, et al. Feasibility and cost-effectiveness of stroke prevention through community screening for atrial fibrillation using iPhone ECG in pharmacies. Thrombosis and haemostasis. 2014;111:1167-76.

[49] Macniven R, Gwynn J, Fujimoto H, Hamilton S, Thompson SC, Taylor K, et al. Feasibility and acceptability of opportunistic screening to detect atrial fibrillation in Aboriginal adults. Australian and New Zealand Journal of Public Health. 2019;43:313-8.

[50] Magnani JW, Schlusser CL, Kimani E, Rollman BL, Paasche-Orlow MK, Bickmore TW. The Atrial Fibrillation Health Literacy Information Technology System: Pilot Assessment. JMIR Cardio. 2017;1:e7.

[51] Orchard J, Neubeck L, Freedman B, Li J, Webster R, Zwar N, et al. eHealth tools to provide structured assistance for atrial fibrillation screening, management, and Guideline-Recommended therapy in metropolitan general practice: the AF-SMART study. Journal of the American Heart Association. 2019;8:e010959.

[52] Orchard J, Freedman SB, Lowres N, Peiris D, Neubeck L. iPhone ECG screening by practice nurses and receptionists for atrial fibrillation in general practice: the GP-SEARCH qualitative pilot study. Australian family physician. 2014;43:315.

[53] Orchard J, Li J, Freedman B, Webster R, Salkeld G, Hespe C, et al. Atrial Fibrillation Screen, Management, and Guideline-Recommended Therapy in the Rural Primary Care Setting: A Cross-Sectional Study and Cost-Effectiveness Analysis of eHealth Tools to Support All Stages of Screening. Journal of the American Heart Association. 2020;9:e017080.

[54] Orchard J, Lowres N, Freedman SB, Ladak L, Lee W, Zwar N, et al. Screening for atrial fibrillation during influenza vaccinations by primary care nurses using a smartphone electrocardiograph (iECG): A feasibility study. European journal of preventive cardiology. 2016;23:13-20.

[55] Rajakariar K, Koshy AN, Sajeev JK, Nair S, Roberts L, Teh AW. Accuracy of a smartwatch based single-lead electrocardiogram device in detection of atrial fibrillation. Heart. 2020;106:665-70.

[56] Reed MJ, Grubb NR, Lang CC, O'Brien R, Simpson K, Padarenga M, et al. Multi-centre Randomised Controlled Trial of a Smartphone-based Event Recorder Alongside Standard Care Versus Standard Care for Patients Presenting to the Emergency Department with Palpitations and Pre-syncope: The IPED (Investigation of Palpitations in the ED) study. EClinicalMedicine. 2019;8:37-46.

[57] Reed MJ, Muir A, Cullen J, Murphy R, Pollard V, Zangana G, et al. Establishing a Smartphone Ambulatory ECG Service for Patients Presenting to the Emergency Department with Pre-Syncope and Palpitations. Medicina (Kaunas). 2021;57.

[58] Rischard J, Waldmann V, Moulin T, Sharifzadehgan A, Lee R, Narayanan K, et al. Assessment of Heart Rhythm Disorders Using the AliveCor Heart Monitor: Beyond the Detection of Atrial Fibrillation. Clinical Electrophysiology. 2020;6:1313-5.

[59] Rosenfeld LE, Amin AN, Hsu JC, Oxner A, Hills MT, Frankel DS. The heart rhythm Society/American College of physicians atrial fibrillation screening and education initiative. Heart Rhythm. 2019;16:e59-e65.

[60] Savickas V, Stewart AJ, Rees-Roberts M, Short V, Bhamra SK, Corlett SA, et al. Opportunistic screening for atrial fibrillation by clinical pharmacists in UK general practice during the influenza vaccination season: A cross-sectional feasibility study. PLoS medicine. 2020;17:e1003197.

[61] Selder J, Breukel L, Blok S, van Rossum A, Tulevski I, Allaart C. A mobile one-lead ECG device incorporated in a symptom-driven remote arrhythmia monitoring program. The first 5,982 Hartwacht ECGs. Netherlands Heart Journal. 2019;27:38-45.

[62] Soni A, Karna S, Fahey N, Sanghai S, Patel H, Raithatha S, et al. Age-and-sex stratified prevalence of atrial fibrillation in rural Western India: results of SMART-India, a population-based screening study. International journal of cardiology. 2019;280:84-8.

[63] Stavrakis S, Stoner JA, Kardokus J, Garabelli PJ, Po SS, Lazzara R. Intermittent vs. Continuous Anticoagulation theRapy in patiEnts with Atrial Fibrillation (iCARE-AF): a randomized pilot study. Journal of Interventional Cardiac Electrophysiology. 2017;48:51-60.

[64] Tarakji KG, Wazni OM, Callahan T, Kanj M, Hakim AH, Wolski K, et al. Using a novel wireless system for monitoring patients after the atrial fibrillation ablation procedure: the iTransmit study. Heart Rhythm. 2015;12:554-9.

[65] Wasserlauf J, You C, Patel R, Valys A, Albert D, Passman R. Smartwatch performance for the detection and quantification of atrial fibrillation. Circulation: Arrhythmia and Electrophysiology. 2019;12:e006834.

[66] Wegner FK, Kochhäuser S, Ellermann C, Lange PS, Frommeyer G, Leitz P, et al. Prospective blinded Evaluation of the smartphone-based AliveCor Kardia ECG monitor for Atrial Fibrillation detection: The PEAK-AF study. European Journal of Internal Medicine. 2020;73:72-5.

[67] William AD, Kanbour M, Callahan T, Bhargava M, Varma N, Rickard J, et al. Assessing the accuracy of an automated atrial fibrillation detection algorithm using smartphone technology: The iREAD Study. Heart rhythm. 2018;15:1561-5.

[68] Williams J, Pearce K, Benett I, Williams J, Manchester M, Pearce K, et al. The effectiveness of a mobile ECG device in identifying AF: sensitivity, specificity and predictive value. Br J Cardiol. 2015;22:70-2.

[69] Yan B, Tu H, Lam C, Swift C, Ho MS, Mok VC, et al. Nurse led smartphone electrographic monitoring for atrial fibrillation after ischemic stroke: SPOT-AF. Journal of Stroke. 2020;22:387.

[70] Zado ES, Pammer M, Parham T, Lin D, Frankel DS, Dixit S, et al. "As Needed" nonvitamin K antagonist oral anticoagulants for infrequent atrial fibrillation episodes following atrial fibrillation ablation guided by diligent pulse monitoring: A feasibility study. Journal of Cardiovascular Electrophysiology. 2019;30:631-8.

[71] Zaprutko T, Zaprutko J, Baszko A, Sawicka D, Szałek A, Dymecka M, et al. Feasibility of Atrial Fibrillation Screening With Mobile Health Technologies at Pharmacies. Journal of Cardiovascular Pharmacology and Therapeutics. 2020;25:142-51.

[72] Aljuaid M, Marashly Q, AlDanaf J, Tawhari I, Barakat M, Barakat R, et al. Smartphone ECG monitoring system helps lower emergency room and clinic visits in post–atrial fibrillation ablation patients. Clinical Medicine Insights: Cardiology. 2020;14:1179546820901508.

[73] Anczykowski J, Willems S, Hoffmann BA, Meinertz T, Blankenberg S, Patten M. Early Detection of Symptomatic Paroxysmal Cardiac Arrhythmias by Trans-Telephonic ECG Monitoring: Impact on Diagnosis and Treatment of Atrial Fibrillation. Journal of cardiovascular electrophysiology. 2016;27:1032-7.

[74] Boriani G, Schnabel RB, Healey JS, Lopes RD, Verbiest-van Gurp N, Lobban T, et al. Consumer-led screening for atrial fibrillation using consumer-facing wearables, devices and apps: A survey of health care professionals by AF-SCREEN international collaboration. European Journal of Internal Medicine. 2020.

[75] Busch MC, Gross S, Alte D, Kors JA, Völzke H, Ittermann T, et al. Impact of atrial fibrillation detected by extended monitoring—A population-based cohort study. Annals of Noninvasive Electrocardiology. 2017;22:e12453.

[76] Chen Y-H, Hung C-S, Huang C-C, Hung Y-C, Hwang J-J, Ho Y-L. Atrial fibrillation screening in nonmetropolitan areas using a telehealth surveillance system with an embedded cloud-computing algorithm: prospective pilot study. JMIR mHealth and uHealth. 2017;5:e135.

[77] Kemp Gudmundsdottir K, Fredriksson T, Svennberg E, Al-Khalili F, Friberg L, Frykman V, et al. Stepwise mass screening for atrial fibrillation using N-terminal B-type natriuretic peptide: the STROKESTOP II study. EP Europace. 2020;22:24-32.

[78] Gussak I, Vukajlovic D, Vukcevic V, George S, Bojovic B, Hadzievski L, et al. Wireless remote monitoring of reconstructed 12-lead ECGs after ablation for atrial fibrillation using a hand-held device. Journal of Electrocardiology. 2012;45:129-35.

[79] Jaakkola J, Jaakkola S, Lahdenoja O, Hurnanen T, Koivisto T, Pänkäälä M, et al. Mobile phone detection of atrial fibrillation with mechanocardiography: The mode-af study (mobile phone detection of atrial fibrillation). Circulation. 2018;137:1524-7.

[80] Liu J, Fang P-h, Hou Y, Li X-f, Liu Y, Wang Y-s, et al. The value of transtelephonic electrocardiogram monitoring system during the "Blanking Period" after ablation of atrial fibrillation. Journal of electrocardiology. 2010;43:667-72.

[81] Olsson A-S, Engdahl J. Detection of atrial fibrillation with intermittent handheld electrocardiogram in patients with ischemic stroke and transient ischemic attack. Journal of Stroke and Cerebrovascular Diseases. 2016;25:2648-52.

[82] Svennberg E, Engdahl J, Al-Khalili F, Friberg L, Frykman V, Rosenqvist M. Mass screening for untreated atrial fibrillation: the STROKESTOP study. Circulation. 2015;131:2176-84.

[83] Vukajlovic D, Gussak I, George S, Simic G, Bojovic B, Hadzievski L, et al. Wireless monitoring of reconstructed 12-lead ECG in atrial fibrillation patients enables differential diagnosis of recurrent arrhythmias. 2011 Annual International Conference of the IEEE Engineering in Medicine and Biology Society: IEEE; 2011. p. 4741-4.

[84] Al-Kaisey AM, Koshy AN, Ha FJ, Spencer R, Toner L, Sajeev JK, et al. Accuracy of wrist-worn heart rate monitors for rate control assessment in atrial fibrillation. Int J Cardiol. 2020;300:161-4.

[85] Bonomi AG, Schipper F, Eerikainen LM, Margarito J, Van Dinther R, Muesch G, et al. Atrial fibrillation detection using a novel cardiac ambulatory monitor based on photo-plethysmography at the wrist. Journal of the american heart association. 2018;7.

[86] Chen E, Jiang J, Su R, Gao M, Zhu S, Zhou J, et al. A new smart wristband equipped with an artificial intelligence algorithm to detect atrial fibrillation. Heart Rhythm. 2020;17:847-53.

[87] Conroy T, Guzman JH, Hall B, Tsouri G, Couderc JP. Detection of atrial fibrillation using an earlobe photoplethysmographic sensor. Physiol Meas. 2017;38:1906-18.

[88] Corino VDA, Laureanti R, Ferranti L, Scarpini G, Lombardi F, Mainardi LT. Detection of atrial fibrillation episodes using a wristband device. Physiol Meas. 2017;38:787-99.

[89] Eerikäinen LM, Dekker L, Bonomi AG, Vullings R, Schipper F, Margarito J, et al. Validating features for atrial fibrillation detection from photoplethysmogram under hospital and free-living conditions. 2017 Computing in Cardiology (CinC): IEEE; 2017. p. 1-4.

[90] Guo Y, Wang H, Zhang H, Liu T, Liang Z, Xia Y, et al. Mobile Photoplethysmographic Technology to Detect Atrial Fibrillation. J Am Coll Cardiol. 2019;74:2365-75.

[91] Hochstadt A, Chorin E, Viskin S, Schwartz AL, Lubman N, Rosso R. Continuous heart rate monitoring for automatic detection of atrial fibrillation with novel bio-sensing technology. J Electrocardiol. 2019;52:23-7.

[92] Huynh P, Shan R, Osuji N, Ding J, Marvel F, Sharma G, et al. ACCURACY OF APPLE WATCH HEART RATE MEASUREMENTS IN PATIENTS WITH OBSTRUCTIVE SLEEP APNEA AND ATRIAL FIBRILLATION. Journal of the American College of Cardiology. 2020;75:3563-.

[93] Huynh P, Shan R, Osuji N, Ding J, Isakadze N, Marvel FA, et al. Heart Rate Measurements in Patients with Obstructive Sleep Apnea and Atrial Fibrillation: Prospective Pilot Study Assessing Apple Watch's Agreement With Telemetry Data. JMIR Cardio. 2021;5:e18050.

[94] Inui T, Kohno H, Kawasaki Y, Matsuura K, Ueda H, Tamura Y, et al. Use of a Smart Watch for Early Detection of Paroxysmal Atrial Fibrillation: Validation Study. JMIR Cardio. 2020;4:e14857.

[95] Jacobsen M, Dembek TA, Ziakos AP, Gholamipoor R, Kobbe G, Kollmann M, et al. Reliable Detection of Atrial Fibrillation with a Medical Wearable during Inpatient Conditions. Sensors (Basel). 2020;20.

[96] Koshy AN, Sajeev JK, Nerlekar N, Brown AJ, Rajakariar K, Zureik M, et al. Smart watches for heart rate assessment in atrial arrhythmias. Int J Cardiol. 2018;266:124-7.

[97] Kwon S, Hong J, Choi EK, Lee B, Baik C, Lee E, et al. Detection of Atrial Fibrillation Using a Ring-Type Wearable Device (CardioTracker) and Deep Learning Analysis of Photoplethysmography Signals: Prospective Observational Proof-of-Concept Study. J Med Internet Res. 2020;22:e16443.

[98] Nemati S, Ghassemi MM, Ambai V, Isakadze N, Levantsevych O, Shah A, et al. Monitoring and detecting atrial fibrillation using wearable technology. Annu Int Conf IEEE Eng Med Biol Soc. 2016;2016:3394-7.

[99] Perez MV, Mahaffey KW, Hedlin H, Rumsfeld JS, Garcia A, Ferris T, et al. Large-Scale Assessment of a Smartwatch to Identify Atrial Fibrillation. N Engl J Med. 2019;381:1909-17.

[100] Selder JL, Proesmans T, Breukel L, Dur O, Gielen W, van Rossum AC, et al. Assessment of a standalone photoplethysmography (PPG) algorithm for detection of atrial fibrillation on wristband-derived data. Comput Methods Programs Biomed. 2020;197:105753.

[101] Seshadri DR, Bittel B, Browsky D, Houghtaling P, Drummond CK, Desai M, et al. Accuracy of the Apple Watch 4 to Measure Heart Rate in Patients With Atrial Fibrillation. IEEE J Transl Eng Health Med. 2020;8:2700204.

[102] Tison GH, Sanchez JM, Ballinger B, Singh A, Olgin JE, Pletcher MJ, et al. Passive Detection of Atrial Fibrillation Using a Commercially Available Smartwatch. JAMA Cardiol. 2018;3:409-16.

[103] Zhang H, Zhang J, Li HB, Chen YX, Yang B, Guo YT, et al. Validation of Single Centre Pre-Mobile Atrial Fibrillation Apps for Continuous Monitoring of Atrial Fibrillation in a Real-World Setting: Pilot Cohort Study. J Med Internet Res. 2019;21:e14909.

[104] Fukuma N, Hasumi E, Fujiu K, Waki K, Toyooka T, Komuro I, et al. Feasibility of a T-Shirt-Type Wearable Electrocardiography Monitor for Detection of Covert Atrial Fibrillation in Young Healthy Adults. Sci Rep. 2019;9:11768.

[105] Heo NJ, Rhee SY, Waalen J, Steinhubl S. Chronic kidney disease and undiagnosed atrial fibrillation in individuals with diabetes. Cardiovasc Diabetol. 2020;19:157.

[106] Reverberi C, Rabia G, De Rosa F, Bosi D, Botti A, Benatti G. The RITMIA™ Smartphone App for Automated Detection of Atrial Fibrillation: Accuracy in Consecutive Patients Undergoing Elective Electrical Cardioversion. Biomed Res Int. 2019;2019:4861951.

[107] Sabar MI, Ara F, Henderson A, Ahmed O, Potter C, John I, et al. A study to assess a novel automated electrocardiogram technology in screening for atrial fibrillation. Pacing Clin Electrophysiol. 2019;42:1383-9.

[108] Steinhubl SR, Waalen J, Edwards AM, Ariniello LM, Mehta RR, Ebner GS, et al. Effect of a Home-Based Wearable Continuous ECG Monitoring Patch on Detection of Undiagnosed Atrial Fibrillation: The mSToPS Randomized Clinical Trial. Jama. 2018;320:146-55.

[109] Torfs T, Smeets CJ, Geng D, Berset T, Van der Auwera J, Vandervoort P, et al. Clinical validation of a low-power and wearable ECG patch for long term full-disclosure monitoring. J Electrocardiol. 2014;47:881-9.

[110] Turakhia MP, Ullal AJ, Hoang DD, Than CT, Miller JD, Friday KJ, et al. Feasibility of extended ambulatory electrocardiogram monitoring to identify silent atrial fibrillation in high-risk patients: the Screening Study for Undiagnosed Atrial Fibrillation (STUDY-AF). Clin Cardiol. 2015;38:285-92.

[111] Wineinger NE, Barrett PM, Zhang Y, Irfanullah I, Muse ED, Steinhubl SR, et al. Identification of paroxysmal atrial fibrillation subtypes in over 13,000 individuals. Heart Rhythm. 2019;16:26-30.

[112] Atarashi H, Ogawa S, Inoue H, Hamada C. Dose-response effect of flecainide in patients with symptomatic paroxysmal atrial fibrillation and/or flutter monitored with trans-telephonic electrocardiography: a multicenter, placebo-controlled, double-blind trial. Circulation journal. 2007;71:294-300.

[113] Brunetti ND, De Gennaro L, Pellegrino PL, Dellegrottaglie G, Antonelli G, Di Biase M. Atrial fibrillation with symptoms other than palpitations: incremental diagnostic sensitivity with at-home tele-cardiology assessment for emergency medical service. Eur J Prev Cardiol. 2012;19:306-13.

[114] Hojager A, Tingsgaard JK, Andersen D, Soholm H, Taskiran M, Bock TG, et al. Silent atrial fibrillation detected by home-monitoring: Cardiovascular disease and stroke prevention in patients with diabetes. J Diabetes Complications. 2020;34:107711.

[115] Kimura T, Aizawa Y, Kurata N, Nakajima K, Kashimura S, Kunitomi A, et al. Assessment of atrial fibrillation ablation outcomes with clinic ECG, monthly 24-h Holter ECG, and twice-daily telemonitoring ECG. Heart Vessels. 2017;32:317-25.

[116] Lin CT, Chang KC, Lin CL, Chiang CC, Lu SW, Chang SS, et al. An intelligent telecardiology system using a wearable and wireless ECG to detect atrial fibrillation. IEEE Trans Inf Technol Biomed. 2010;14:726-33.

[117] Scalvini S, Piepoli M, Zanelli E, Volterrani M, Giordano A, Glisenti F. Incidence of atrial fibrillation in an Italian population followed by their GPs through a telecardiology service. Int J Cardiol. 2005;98:215-20.

[118] Wu CF, Yang CY, Li AH, Chuang WP, Chen KC, Liu YH, et al. Detection of asymptomatic paroxysmal atrial fibrillation with the trans-telephonic electrocardiograph system. Telemed J E Health. 2012;18:193-7.

[119] Gandolfo C, Balestrino M, Bruno C, Finocchi C, Reale N. Validation of a simple method for atrial fibrillation screening in patients with stroke. Neurol Sci. 2015;36:1675-8.

[120] Kearley K, Selwood M, Van den Bruel A, Thompson M, Mant D, Hobbs FR, et al. Triage tests for identifying atrial fibrillation in primary care: a diagnostic accuracy study comparing single-lead ECG and modified BP monitors. BMJ Open. 2014;4:e004565.

[121] Marazzi G, Iellamo F, Volterrani M, Lombardo M, Pelliccia F, Righi D, et al. Comparison of Microlife BP A200 Plus and Omron M6 blood pressure monitors to detect atrial fibrillation in hypertensive patients. Adv Ther. 2012;29:64-70.

[122] Omboni S, Verberk WJ. Opportunistic screening of atrial fibrillation by automatic blood pressure measurement in the community. BMJ Open. 2016;6:e010745.

[123] Stergiou GS, Karpettas N, Protogerou A, Nasothimiou EG, Kyriakidis M. Diagnostic accuracy of a home blood pressure monitor to detect atrial fibrillation. J Hum Hypertens. 2009;23:654-8.

[124] Wiesel J, Abraham S, Messineo FC. Screening for asymptomatic atrial fibrillation while monitoring the blood pressure at home: trial of regular versus irregular pulse for prevention of stroke (TRIPPS 2.0). Am J Cardiol. 2013;111:1598-601.

[125] Wiesel J, Fitzig L, Herschman Y, Messineo FC. Detection of atrial fibrillation using a modified microlife blood pressure monitor. Am J Hypertens. 2009;22:848-52.

[126] Wiesel J, Wiesel D, Suri R, Messineo FC. The use of a modified sphygmomanometer to detect atrial fibrillation in outpatients. Pacing Clin Electrophysiol. 2004;27:639-43.

[127] Lauschke J, Busch M, Haverkamp W, Bulava A, Schneider R, Andresen D, et al. New implantable cardiac monitor with three-lead ECG and active noise detection. Herz. 2017;42:585-92.

[128] Ciconte G, Saviano M, Giannelli L, Calovic Z, Baldi M, Ciaccio C, et al. Atrial fibrillation detection using a novel three-vector cardiac implantable monitor: the atrial fibrillation detect study. Europace. 2017;19:1101-8.

[129] Sanders P, Purerfellner H, Pokushalov E, Sarkar S, Di Bacco M, Maus B, et al. Performance of a new atrial fibrillation detection algorithm in a miniaturized insertable cardiac monitor: Results from the Reveal LINQ Usability Study. Heart Rhythm. 2016;13:1425-30.

[130] Purerfellner H, Sanders P, Sarkar S, Reisfeld E, Reiland J, Koehler J, et al. Adapting detection sensitivity based on evidence of irregular sinus arrhythmia to improve atrial fibrillation detection in insertable cardiac monitors. Europace. 2018;20:f321-f8.

[131] Hindricks G, Pokushalov E, Urban L, Taborsky M, Kuck KH, Lebedev D, et al. Performance of a new leadless implantable cardiac monitor in detecting and quantifying atrial fibrillation: Results of the XPECT trial. Circ Arrhythm Electrophysiol. 2010;3:141-7.

[132] Nolker G, Mayer J, Boldt LH, Seidl K, V VAND, Massa T, et al. Performance of an Implantable Cardiac Monitor to Detect Atrial Fibrillation: Results of the DETECT AF Study. J Cardiovasc Electrophysiol. 2016;27:1403-10.

[133] Asaithambi G, Monita JE, Annamalai MR, Ho BM, Marino EH, Hanson SK. Prevalence of atrial fibrillation with insertable cardiac monitors in cryptogenic stroke: A single-center experience. J Electrocardiol. 2018;51:973-6.

[134] Bergau L, Sohns C, Sossalla S, Munoz-Exposito P, Luethje L, Zabel M. Submuscular implantation of insertable cardiac monitors improves the reliability of detection of atrial fibrillation. J Interv Card Electrophysiol. 2015;42:143-9.

[135] Bertelsen L, Diederichsen SZ, Haugan KJ, Brandes A, Graff C, Krieger D, et al. Left Atrial Late Gadolinium Enhancement is Associated With Incident Atrial Fibrillation as Detected by Continuous Monitoring With Implantable Loop Recorders. JACC Cardiovasc Imaging. 2020;13:1690-700.

[136] Carrazco C, Golyan D, Kahen M, Black K, Libman RB, Katz JM. Prevalence and Risk Factors for Paroxysmal Atrial Fibrillation and Flutter Detection after Cryptogenic Ischemic Stroke. J Stroke Cerebrovasc Dis. 2018;27:203-9.

[137] Choe WC, Passman RS, Brachmann J, Morillo CA, Sanna T, Bernstein RA, et al. A Comparison of Atrial Fibrillation Monitoring Strategies After Cryptogenic Stroke (from the Cryptogenic Stroke and Underlying AF Trial). Am J Cardiol. 2015;116:889-93.

[138] Chorin E, Peterson C, Kogan E, Barbhaiya C, Aizer A, Holmes D, et al. Comparison of the Effect of Atrial Fibrillation Detection Algorithms in Patients With Cryptogenic Stroke Using Implantable Loop Recorders. Am J Cardiol. 2020;129:25-9.

[139] Christensen LM, Krieger DW, Hojberg S, Pedersen OD, Karlsen FM, Jacobsen MD, et al. Paroxysmal atrial fibrillation occurs often in cryptogenic ischaemic stroke. Final results from the SURPRISE study. Eur J Neurol. 2014;21:884-9.

[140] Cotter PE, Martin PJ, Ring L, Warburton EA, Belham M, Pugh PJ. Incidence of atrial fibrillation detected by implantable loop recorders in unexplained stroke. Neurology. 2013;80:1546-50.

[141] Dekker LR, Pokushalov E, Sanders P, Lindborg KA, Maus B, Purerfellner H. Continuous Cardiac Monitoring around Atrial Fibrillation Ablation: Insights on Clinical Classifications and End Points. Pacing Clin Electrophysiol. 2016;39:805-13.

[142] De With RR, Erkuner O, Rienstra M, Nguyen BO, Korver FWJ, Linz D, et al. Temporal patterns and short-term progression of paroxysmal atrial fibrillation: data from RACE V. Europace. 2020;22:1162-72.

[143] Diederichsen SZ, Haugan KJ, Brandes A, Graff C, Krieger D, Kronborg C, et al. Incidence and predictors of atrial fibrillation episodes as detected by implantable loop recorder in patients at risk: From the LOOP study. Am Heart J. 2020;219:117-27.

[144] Dion F, Saudeau D, Bonnaud I, Friocourt P, Bonneau A, Poret P, et al. Unexpected low prevalence of atrial fibrillation in cryptogenic ischemic stroke: a prospective study. J Interv Card Electrophysiol. 2010;28:101-7.

[145] Etgen T, Hochreiter M, Mundel M, Freudenberger T. Insertable cardiac event recorder in detection of atrial fibrillation after cryptogenic stroke: an audit report. Stroke. 2013;44:2007-9.

[146] Forkmann M, Schwab C, Edler D, Vevecka A, Butz S, Haller B, et al. Characteristics of early recurrences detected by continuous cardiac monitoring influencing the long-term outcome after atrial fibrillation ablation. J Cardiovasc Electrophysiol. 2019;30:1886-93.

[147] Haldar S, Khan HR, Boyalla V, Kralj-Hans I, Jones S, Lord J, et al. Catheter ablation vs. thoracoscopic surgical ablation in long-standing persistent atrial fibrillation: CASA-AF randomized controlled trial. Eur Heart J. 2020.

[148] Healey JS, Alings M, Ha A, Leong-Sit P, Birnie DH, de Graaf JJ, et al. Subclinical Atrial Fibrillation in Older Patients. Circulation. 2017;136:1276-83.

[149] Israel C, Kitsiou A, Kalyani M, Deelawar S, Ejangue LE, Rogalewski A, et al. Detection of atrial fibrillation in patients with embolic stroke of undetermined source by prolonged monitoring with implantable loop recorders. Thromb Haemost. 2017;117:1962-9.

[150] Jorfida M, Antolini M, Cerrato E, Caprioli MG, Castagno D, Garrone P, et al. Cryptogenic ischemic stroke and prevalence of asymptomatic atrial fibrillation: a prospective study. J Cardiovasc Med (Hagerstown). 2016;17:863-9.

[151] Kitsiou A, Rogalewski A, Kalyani M, Deelawar S, Tribunyan S, Greeve I, et al. Atrial fibrillation in patients with embolic stroke of undetermined source during 3 years of prolonged monitoring with an implantable loop recorder. Thromb Haemost. 2021.

[152] Kusiak A, Jastrzebski M, Bednarski A, Kulakowski P, Piotrowski R, Kozluk E, et al. Diagnostic value of implantable loop recorder in patients undergoing cryoballoon ablation of atrial fibrillation. Ann Noninvasive Electrocardiol. 2020;25:e12733.

[153] Lacour P, Dang PL, Huemer M, Parwani AS, Attanasio P, Pieske B, et al. Performance of the New BioMonitor 2-AF Insertable Cardiac Monitoring System: Can Better be Worse? Pacing Clin Electrophysiol. 2017;40:516-26.

[154] Makimoto H, Kurt M, Gliem M, Lee JI, Schmidt J, Muller P, et al. High Incidence of Atrial Fibrillation After Embolic Stroke of Undetermined Source in Posterior Cerebral Artery Territory. J Am Heart Assoc. 2017;6.

[155] Marks D, Ho R, Then R, Weinstock JL, Teklemariam E, Kakadia B, et al. Real-world experience with implantable loop recorder monitoring to detect subclinical atrial fibrillation in patients with cryptogenic stroke: The value of p wave dispersion in predicting arrhythmia occurrence. Int J Cardiol. 2020.

[156] Merce J, Garcia M, Ustrell X, Pellise A, de Castro R, Bardaji A. Implantable loop recorder: a new tool in the diagnosis of cryptogenic stroke. Rev Esp Cardiol (Engl Ed). 2013;66:665-6.

[157] Muller P, Ivanov V, Kara K, Klein-Wiele O, Forkmann M, Piorkowski C, et al. Total atrial conduction time to predict occult atrial fibrillation after cryptogenic stroke. Clin Res Cardiol. 2017;106:113-9.

[158] Nasir JM, Pomeroy W, Marler A, Hann M, Baykaner T, Jones R, et al. Predicting Determinants of Atrial Fibrillation or Flutter for Therapy Elucidation in Patients at Risk for Thromboembolic Events (PREDATE AF) Study. Heart Rhythm. 2017;14:955-61.

[159] Pedersen KB, Madsen C, Sandgaard NCF, Diederichsen ACP, Bak S, Brandes A. Subclinical atrial fibrillation in patients with recent transient ischemic attack. J Cardiovasc Electrophysiol. 2018;29:707-14.

[160] Poli S, Diedler J, Hartig F, Gotz N, Bauer A, Sachse T, et al. Insertable cardiac monitors after cryptogenic stroke--a risk factor based approach to enhance the detection rate for paroxysmal atrial fibrillation. Eur J Neurol. 2016;23:375-81.

[161] Prabhu S, Taylor AJ, Costello BT, Kaye DM, McLellan AJA, Voskoboinik A, et al. Catheter Ablation Versus Medical Rate Control in Atrial Fibrillation and Systolic Dysfunction: The CAMERA-MRI Study. J Am Coll Cardiol. 2017;70:1949-61.

[162] Reiffel JA, Verma A, Kowey PR, Halperin JL, Gersh BJ, Wachter R, et al. Incidence of Previously Undiagnosed Atrial Fibrillation Using Insertable Cardiac Monitors in a High-Risk Population: The REVEAL AF Study. JAMA Cardiol. 2017;2:1120-7.

[163] Reinke F, Bettin M, Ross LS, Kochhauser S, Kleffner I, Ritter M, et al. Refinement of detecting atrial fibrillation in stroke patients: results from the TRACK-AF Study. Eur J Neurol. 2018;25:631-6.

[164] Ritter MA, Kochhauser S, Duning T, Reinke F, Pott C, Dechering DG, et al. Occult atrial fibrillation in cryptogenic stroke: detection by 7-day electrocardiogram versus implantable cardiac monitors. Stroke. 2013;44:1449-52.

[165] Pokushalov E, Kozlov B, Romanov A, Strelnikov A, Bayramova S, Sergeevichev D, et al. Long-Term Suppression of Atrial Fibrillation by Botulinum Toxin Injection Into Epicardial Fat Pads in Patients Undergoing Cardiac Surgery: One-Year Follow-Up of a Randomized Pilot Study. Circ Arrhythm Electrophysiol. 2015;8:1334-41.

[166] Sanna T, Diener HC, Passman RS, Di Lazzaro V, Bernstein RA, Morillo CA, et al. Cryptogenic stroke and underlying atrial fibrillation. N Engl J Med. 2014;370:2478-86.
[167] Seow SC, How AK, Chan SP, Teoh HL, Lim TW, Singh D, et al. High Incidence of Occult Atrial Fibrillation in Asian Patients with Cryptogenic Stroke. J Stroke Cerebrovasc Dis. 2018;27:2182-6.
[168] Victor CU, Carolina PE, Jorge TR, Joaquin CR, Manuel SG, Marta CM, et al. Incidence and Predictive Factors of Hidden Atrial Fibrillation Detected by Implantable Loop Recorder After an Embolic Stroke of Undetermined Source. J Atr Fibrillation. 2018;11:2078.
[169] Wasserlauf J, You C, Patel R, Valys A, Albert D, Passman R. Smartwatch Performance for the Detection and Quantification of Atrial Fibrillation. Circ Arrhythm Electrophysiol. 2019;12:e006834.
[170] Watson RA, Wellings J, Hingorani R, Zhan T, Frisch DR, Ho RT, et al. Atrial fibrillation post central retinal artery occlusion: Role of implantable loop recorders. Pacing Clin Electrophysiol. 2020.
[171] Wechselberger S, Kronborg M, Huo Y, Piorkowski J, Neudeck S, Passler E, et al. Continuous monitoring after atrial fibrillation ablation: the LINQ AF study. Europace. 2018;20:f312-f20.
[172] Xu J, Sethi P, Biby S, Allred J, Seiler A, Sabir R. Predictors of atrial fibrillation detection and features of recurrent strokes in patients after cryptogenic stroke. J Stroke Cerebrovasc Dis. 2020;29:104934.
[173] Yaeger A, Keenan BT, Cash NR, Parham T, Deo R, Frankel DS, et al. Impact of a nurse-led limited risk factor modification program on arrhythmia outcomes in patients with atrial fibrillation undergoing catheter ablation. J Cardiovasc Electrophysiol. 2020;31:423-31.
[174] Ziegler PD, Rogers JD, Ferreira SW, Nichols AJ, Richards M, Koehler JL, et al. Long-term detection of atrial fibrillation with insertable cardiac monitors in a real-world cryptogenic stroke population. Int J Cardiol. 2017;244:175-9.

[175] Ziegler PD, Rogers JD, Ferreira SW, Nichols AJ, Sarkar S, Koehler JL, et al. Real-World Experience with Insertable Cardiac Monitors to Find Atrial Fibrillation in Cryptogenic Stroke. Cerebrovasc Dis. 2015;40:175-81.

[176] Desteghe L, Kluts K, Vijgen J, Koopman P, Dilling-Boer D, Schurmans J, et al. The Health Buddies App as a Novel Tool to Improve Adherence and Knowledge in Atrial Fibrillation Patients: A Pilot Study. JMIR Mhealth Uhealth. 2017;5:e98.

[177] Ghanbari H, Ansari S, Ghannam M, Lathkar-Pradhan S, Kratz A, Oral H, et al. Feasibility and Usability of a Mobile Application to Assess Symptoms and Affect in Patients with Atrial Fibrillation: A Pilot Study. J Atr Fibrillation. 2017;10:1672.

[178] Guo Y, Lane DA, Wang L, Zhang H, Wang H, Zhang W, et al. Mobile Health Technology to Improve Care for Patients With Atrial Fibrillation. J Am Coll Cardiol. 2020;75:1523-34.

[179] Guo Y, Lane DA, Chen Y, Lip GYH, m AFAIITi. Regular Bleeding Risk Assessment Associated with Reduction in Bleeding Outcomes: The mAFA-II Randomized Trial. Am J Med. 2020;133:1195-202 e2.

[180] Guo Y, Chen Y, Lane DA, Liu L, Wang Y, Lip GYH. Mobile Health Technology for Atrial Fibrillation Management Integrating Decision Support, Education, and Patient Involvement: mAF App Trial. Am J Med. 2017;130:1388-96 e6.

[181] Guo Y, Guo J, Shi X, Yao Y, Sun Y, Xia Y, et al. Mobile health technology-supported atrial fibrillation screening and integrated care: A report from the mAFA-II trial Long-term Extension Cohort. Eur J Intern Med. 2020.

[182] Hirschey J, Bane S, Mansour M, Sperber J, Agboola S, Kvedar J, et al. Evaluating the Usability and Usefulness of a Mobile App for Atrial Fibrillation Using Qualitative Methods: Exploratory Pilot Study. JMIR Hum Factors. 2018;5:e13.

[183] Manimaran M, Das D, Martinez P, Schwartz R, Schilling R, Finlay M. The impact of virtual arrhythmia clinics following catheter ablation for atrial fibrillation. Eur Heart J Qual Care Clin Outcomes. 2019;5:272-3.

[184] Stephan LS, Almeida ED, Guimaraes RB, Ley AG, Mathias RG, Assis MV, et al. Oral Anticoagulation in Atrial Fibrillation: Development and Evaluation of a Mobile Health Application to Support Shared Decision-Making. Arq Bras Cardiol. 2018;110:7-15.

[185] Balsam P, Borodzicz S, Malesa K, Puchta D, Tyminska A, Ozieranski K, et al. OCULUS study: Virtual reality-based education in daily clinical practice. Cardiol J. 2019;26:260-4.

[186] Desteghe L, Germeys J, Vijgen J, Koopman P, Dilling-Boer D, Schurmans J, et al. Effectiveness and usability of an online tailored education platform for atrial fibrillation patients undergoing a direct current cardioversion or pulmonary vein isolation. Int J Cardiol. 2018;272:123-9.

[187] Goni L, de la OV, Barrio-López MT, Ramos P, Tercedor L, Ibañez-Criado JL, et al. A Remote Nutritional Intervention to Change the Dietary Habits of Patients Undergoing Ablation of Atrial Fibrillation: Randomized Controlled Trial. J Med Internet Res. 2020;22:e21436.

[188] Guhl E, Althouse AD, Pusateri AM, Kimani E, Paasche-Orlow MK, Bickmore TW, et al. The Atrial Fibrillation Health Literacy Information Technology Trial: Pilot Trial of a Mobile Health App for Atrial Fibrillation. JMIR Cardio. 2020;4:e17162.

[189] Carter L, Gardner M, Magee K, Fearon A, Morgulis I, Doucette S, et al. An Integrated Management Approach to Atrial Fibrillation. J Am Heart Assoc. 2016;5.

[190] Ferguson C, Hickman LD, Phillips J, Newton PJ, Inglis SC, Lam L, et al. An mHealth intervention to improve nurses' atrial fibrillation and anticoagulation knowledge and practice: the EVICOAG study. Eur J Cardiovasc Nurs. 2019;18:7-15.

[191] Mesquita J, Maniar N, Baykaner T, Rogers AJ, Swerdlow M, Alhusseini MI, et al. Online webinar training to analyse complex atrial fibrillation maps: A randomized trial. PLoS One. 2019;14:e0217988.

[192] Cox JL, Parkash R, Foster GA, Xie F, MacKillop JH, Ciaccia A, et al. Integrated Management Program Advancing Community Treatment of Atrial Fibrillation (IMPACT-AF): A cluster randomized trial of a computerized clinical decision support tool. Am Heart J. 2020;224:35-46.

[193] Eckman MH, Costea A, Attari M, Munjal J, Wise RE, Knochelmann C, et al. Shared decision-making tool for thromboprophylaxis in atrial fibrillation - A feasibility study. Am Heart J. 2018;199:13-21.

[194] Eckman MH, Lip GY, Wise RE, Speer B, Sullivan M, Walker N, et al. Impact of an Atrial Fibrillation Decision Support Tool on thromboprophylaxis for atrial fibrillation. Am Heart J. 2016;176:17-27.

[195] Hendriks JM, de Wit R, Crijns HJ, Vrijhoef HJ, Prins MH, Pisters R, et al. Nurse-led care vs. usual care for patients with atrial fibrillation: results of a randomized trial of integrated chronic care vs. routine clinical care in ambulatory patients with atrial fibrillation. Eur Heart J. 2012;33:2692-9.

[196] Karlsson LO, Nilsson S, Bang M, Nilsson L, Charitakis E, Janzon M. A clinical decision support tool for improving adherence to guidelines on anticoagulant therapy in patients with atrial fibrillation at risk of stroke: A cluster-randomized trial in a Swedish primary care setting (the CDS-AF study). PLoS Med. 2018;15:e1002528.

[197] Sheibani R, Sheibani M, Heidari-Bakavoli A, Abu-Hanna A, Eslami S. The Effect of a Clinical Decision Support System on Improving Adherence to Guideline in the Treatment of Atrial Fibrillation: An Interrupted Time Series Study. J Med Syst. 2017;42:26.

[198] Thomson RG, Eccles MP, Steen IN, Greenaway J, Stobbart L, Murtagh MJ, et al. A patient decision aid to support shared decision-making on anti-thrombotic treatment of patients with atrial fibrillation: randomised controlled trial. Qual Saf Health Care. 2007;16:216-23.

[199] Wijtvliet E, Tieleman RG, van Gelder IC, Pluymaekers N, Rienstra M, Folkeringa RJ, et al. Nurse-led vs. usual-care for atrial fibrillation. Eur Heart J. 2020;41:634-41.

[200] Rosier A, Mabo P, Temal L, Van Hille P, Dameron O, Deleger L, et al. Personalized and automated remote monitoring of atrial fibrillation. Europace. 2016;18:347-52.

[201] Zoppo F, Facchin D, Molon G, Zanotto G, Catanzariti D, Rossillo A, et al. Improving atrial fibrillation detection in patients with implantable cardiac devices by means of a remote monitoring and management application. Pacing Clin Electrophysiol. 2014;37:1610-8.

[202] Shacham J, Birati EY, Malov N, Yanay Y, Steinberg DM, Tamari M, et al. Telemedicine for diagnosing and managing paroxysmal atrial fibrillation in outpatients. The phone in the pocket. Int J Cardiol. 2012;157:91-5.

[203] Stegmann T, Koehler K, Wachter R, Moeller V, Zeynalova S, Koehler F, et al. Heart failure patients with atrial fibrillation benefit from remote patient management: insights from the TIM-HF2 trial. ESC Heart Fail. 2020;7:2516-26.

[204] Jiang J, Gu X, Cheng CD, Li HX, Sun XL, Duan RY, et al. The Hospital-Community-Family-Based Telemedicine (HCFT-AF) Program for Integrative Management of Patients With Atrial Fibrillation: Pilot Feasibility Study. JMIR Mhealth Uhealth. 2020;8:e22137.

[205] Goette A, Schön N, Kirchhof P, Breithardt G, Fetsch T, Häusler KG, et al. Angiotensin II-antagonist in paroxysmal atrial fibrillation (ANTIPAF) trial. Circ Arrhythm Electrophysiol. 2012;5:43-51.

[206] Stewart S, Ball J, Horowitz JD, Marwick TH, Mahadevan G, Wong C, et al. Standard versus atrial fibrillation-specific management strategy (SAFETY) to reduce recurrent admission and prolong survival: pragmatic, multicentre, randomised controlled trial. Lancet. 2015;385:775-84.

[207] Vinereanu D, Lopes RD, Bahit MC, Xavier D, Jiang J, Al-Khalidi HR, et al. A multifaceted intervention to improve treatment with oral anticoagulants in atrial fibrillation (IMPACT-AF): an international, cluster-randomised trial. Lancet. 2017;390:1737-46.

[208] Peleg M, Shahar Y, Quaglini S, Broens T, Budasu R, Fung N, et al. Assessment of a personalized and distributed patient guidance system. Int J Med Inform. 2017;101:108-30.

[209] Lau JK, Lowres N, Neubeck L, Brieger DB, Sy RW, Galloway CD, et al. iPhone ECG application for community screening to detect silent atrial fibrillation: a novel technology to prevent stroke. Int J Cardiol. 2013;165:193-4.

[210] Lee J, Reyes BA, McManus DD, Maitas O, Chon KH. Atrial fibrillation detection using an iPhone 4S. IEEE Trans Biomed Eng. 2013;60:203-6.

[211] McManus DD, Lee J, Maitas O, Esa N, Pidikiti R, Carlucci A, et al. A novel application for the detection of an irregular pulse using an iPhone 4S in patients with atrial fibrillation. Heart Rhythm. 2013;10:315-9.

[212] Shrivastav M, Padte S, Arora V, Biffi M. Pilot evaluation of an integrated monitor-adhesive patch for long-term cardiac arrhythmia detection in India. Expert Rev Cardiovasc Ther. 2014;12:25-35.

[213] Chung EH, Guise KD. QTC intervals can be assessed with the AliveCor heart monitor in patients on dofetilide for atrial fibrillation. J Electrocardiol. 2015;48:8-9.

[214] Marcolino MS, Palhares DM, Benjamin EJ, Ribeiro AL. Atrial fibrillation: prevalence in a large database of primary care patients in Brazil. Europace. 2015;17:1787-90.

[215] Nguyen HH, Van Hare GF, Rudokas M, Bowman T, Silva JN. SPEAR Trial: Smartphone Pediatric ElectrocARdiogram Trial. PLoS One. 2015;10:e0136256.

[216] Weidemann F, Maier SK, Störk S, Brunner T, Liu D, Hu K, et al. Usefulness of an Implantable Loop Recorder to Detect Clinically Relevant Arrhythmias in Patients With Advanced Fabry Cardiomyopathy. Am J Cardiol. 2016;118:264-74.

[217] Coppetti T, Brauchlin A, Müggler S, Attinger-Toller A, Templin C, Schönrath F, et al. Accuracy of smartphone apps for heart rate measurement. Eur J Prev Cardiol. 2017;24:1287-93.

[218] Chong JW, Cho CH, Tabei F, Le-Anh D, Esa N, McManus DD, et al. Motion and Noise Artifact-Resilient Atrial Fibrillation Detection using a Smartphone. IEEE J Emerg Sel Top Circuits Syst. 2018;8:230-9.

[219] Lahdenoja O, Hurnanen T, Iftikhar Z, Nieminen S, Knuutila T, Saraste A, et al. Atrial Fibrillation Detection via Accelerometer and Gyroscope of a Smartphone. IEEE J Biomed Health Inform. 2018;22:108-18.

[220] Nguyen A, Ansari S, Hooshmand M, Lin K, Ghanbari H, Gryak J, et al. Comparative Study on Heart Rate Variability Analysis for Atrial Fibrillation Detection in Short Single-Lead ECG Recordings. Annu Int Conf IEEE Eng Med Biol Soc. 2018;2018:526-9.

[221] Baca-Motes K, Edwards AM, Waalen J, Edmonds S, Mehta RR, Ariniello L, et al. Digital recruitment and enrollment in a remote nationwide trial of screening for undiagnosed atrial fibrillation: Lessons from the randomized, controlled mSToPS trial. Contemp Clin Trials Commun. 2019;14:100318.

[222] Marinucci D, Sbrollini A, Marcantoni I, Morettini M, Swenne CA, Burattini L. Artificial Neural Network for Atrial Fibrillation Identification in Portable Devices. Sensors (Basel). 2020;20.

[223] Luo C, Li Q, Rao H, Huang X, Jiang H, Rao N. An improved Poincaré plot-based method to detect atrial fibrillation from short single-lead ECG. Biomedical Signal Processing and Control. 2020;64:102264.
