# Supplementary material for: Mobile health solutions for atrial fibrillation detection and management: a systematic review
Source: Clin Res Cardiol. 2021 Sep 21;111(5):479–91. doi: 10.1007/s00392-021-01941-9 (PMC8454991; doi:10.1007/s00392-021-01941-9)
Supplement: Supplementary file 2 — Supplementary file2 (DOCX 41 KB) [file 392_2021_1941_MOESM2_ESM.docx]

**Supplementary material**

**References 1-232**

1. Chugh SS, Roth GA, Gillum RF, Mensah GA. Global burden of atrial fibrillation in developed and developing nations. Glob Heart. 2014;9(1):113-9.

2. Dai H, Zhang Q, Much AA, Maor E, Segev A, Beinart R, et al. Global, regional, and national prevalence, incidence, mortality, and risk factors for atrial fibrillation, 1990-2017: results from the Global Burden of Disease Study 2017. Eur Heart J Qual Care Clin Outcomes. 2020.

3. Hindricks G, Potpara T, Dagres N, Arbelo E, Bax JJ, Blomstrom-Lundqvist C, et al. 2020 ESC Guidelines for the diagnosis and management of atrial fibrillation developed in collaboration with the European Association of Cardio-Thoracic Surgery (EACTS). Eur Heart J. 2020.

4. Varma N, Cygankiewicz I, Turakhia M, Heidbuchel H, Hu Y, Chen LY, et al. 2021 ISHNE/ HRS/ EHRA/ APHRS collaborative statement on mHealth in Arrhythmia Management: Digital Medical Tools for Heart Rhythm Professionals: From the International Society for Holter and Noninvasive Electrocardiology/Heart Rhythm Society/European Heart Rhythm Association/Asia Pacific Heart Rhythm Society. Ann Noninvasive Electrocardiol. 2021;26(2):e12795.

5. Moher D, Liberati A, Tetzlaff J, Altman DG, Group P. Preferred reporting items for systematic reviews and meta-analyses: the PRISMA statement. BMJ. 2009;339:b2535.

6. Proesmans T, Mortelmans C, Van Haelst R, Verbrugge F, Vandervoort P, Vaes B. Mobile Phone–Based Use of the Photoplethysmography Technique to Detect Atrial Fibrillation in Primary Care: Diagnostic Accuracy Study of the FibriCheck App. JMIR mHealth and uHealth. 2019;7(3):e12284.

7. Van Haelst R, editor The diagnostic accuracy of smartphone applications to detect atrial fibrillation: a head-to-head comparison between Fibricheck and AliveCor. ACTA CARDIOLOGICA; 2017: TAYLOR & FRANCIS LTD 2-4 PARK SQUARE, MILTON PARK, ABINGDON OR14 4RN, OXON ….

8. Verbrugge FH, Proesmans T, Vijgen J, Mullens W, Rivero-Ayerza M, Van Herendael H, et al. Atrial fibrillation screening with photo-plethysmography through a smartphone camera. EP Europace. 2019;21(8):1167-75.

9. Yan BP, Lai WH, Chan CK, Chan SCH, Chan LH, Lam KM, et al. Contact‐free screening of atrial fibrillation by a smartphone using facial pulsatile photoplethysmographic signals. Journal of the American Heart Association. 2018;7(8):e008585.

10. Rozen G, Vaid J, Hosseini SM, Kaadan MI, Rafael A, Roka A, et al. Diagnostic accuracy of a novel mobile phone application for the detection and monitoring of atrial fibrillation. The American journal of cardiology. 2018;121(10):1187-91.

11. Chan PH, Wong CK, Poh YC, Pun L, Leung WWC, Wong YF, et al. Diagnostic performance of a smartphone‐based photoplethysmographic application for atrial fibrillation screening in a primary care setting. Journal of the American Heart Association. 2016;5(7):e003428.

12. Brasier N, Raichle CJ, Dörr M, Becke A, Nohturfft V, Weber S, et al. Detection of atrial fibrillation with a smartphone camera: first prospective, international, two-centre, clinical validation study (DETECT AF PRO). Ep Europace. 2019;21(1):41-7.

13. Fan Y-Y, Li Y-G, Li J, Cheng W-K, Shan Z-L, Wang Y-T, et al. Diagnostic performance of a smart device with photoplethysmography technology for atrial fibrillation detection: pilot study (Pre-mAFA II registry). JMIR mHealth and uHealth. 2019;7(3):e11437.

14. Birkemeyer R, Müller A, Wahler S, von der Schulenburg J-M. A cost-effectiveness analysis model of Preventicus atrial fibrillation screening from the point of view of statutory health insurance in Germany. Health Economics Review. 2020;10(1):1-18.

15. Krivoshei L, Weber S, Burkard T, Maseli A, Brasier N, Kühne M, et al. Smart detection of atrial fibrillation. Europace. 2017;19(5):753-7.

16. Mutke MR, Brasier N, Raichle C, Ravanelli F, Doerr M, Eckstein J. Comparison and Combination of Single-Lead ECG and Photoplethysmography Algorithms for Wearable-Based Atrial Fibrillation Screening. Telemedicine and e-Health. 2020.

17. McManus DD, Chong JW, Soni A, Saczynski JS, Esa N, Napolitano C, et al. PULSE‐SMART: pulse‐based arrhythmia discrimination using a novel smartphone application. Journal of cardiovascular electrophysiology. 2016;27(1):51-7.

18. Chan PH, Wong CK, Poh YC, Pun L, Leung WW, Wong YF, et al. Diagnostic Performance of a Smartphone-Based Photoplethysmographic Application for Atrial Fibrillation Screening in a Primary Care Setting. J Am Heart Assoc. 2016;5(7).

19. Jacobs MS, Kaasenbrood F, Postma MJ, van Hulst M, Tieleman RG. Cost-effectiveness of screening for atrial fibrillation in primary care with a handheld, single-lead electrocardiogram device in the Netherlands. Ep Europace. 2018;20(1):12-8.

20. Pluymaekers N, Dudink E, Luermans J, Meeder JG, Lenderink T, Widdershoven J, et al. Early or Delayed Cardioversion in Recent-Onset Atrial Fibrillation. N Engl J Med. 2019;380(16):1499-508.

21. Battipaglia I, Gilbert K, Hogarth AJ, Tayebjee MH. Screening for atrial fibrillation in the community using a novel ECG recorder. Journal of atrial fibrillation. 2016;9(2).

22. Kaasenbrood F, Hollander M, Rutten FH, Gerhards LJ, Hoes AW, Tieleman RG. Yield of screening for atrial fibrillation in primary care with a hand-held, single-lead electrocardiogram device during influenza vaccination. Ep Europace. 2016;18(10):1514-20.

23. Rivezzi F, Vio R, Bilato C, Pagliani L, Pasquetto G, Saccà S, et al. Screening of unknown atrial fibrillation through handheld device in the elderly. Journal of Geriatric Cardiology: JGC. 2020;17(8):495-501.

24. Desteghe L, Raymaekers Z, Lutin M, Vijgen J, Dilling-Boer D, Koopman P, et al. Performance of handheld electrocardiogram devices to detect atrial fibrillation in a cardiology and geriatric ward setting. Europace. 2017;19(1):29-39.

25. Tavernier R, Wolf M, Kataria V, Phlips T, Huys R, Taghji P, et al. Screening for atrial fibrillation in hospitalised geriatric patients. Heart. 2018;104(7):588-93.

26. Vaes B, Stalpaert S, Tavernier K, Thaels B, Lapeire D, Mullens W, et al. The diagnostic accuracy of the MyDiagnostick to detect atrial fibrillation in primary care. BMC family practice. 2014;15(1):1-7.

27. Tieleman R, Plantinga Y, Rinkes D, Bartels G, Posma J, Cator R, et al. Validation and clinical use of a novel diagnostic device for screening of atrial fibrillation. Europace. 2014;16(9):1291-5.

28. Macniven R, Gwynn J, Fujimoto H, Hamilton S, Thompson SC, Taylor K, et al. Feasibility and acceptability of opportunistic screening to detect atrial fibrillation in Aboriginal adults. Aust N Z J Public Health. 2019;43(4):313-8.

29. Magnani JW, Schlusser CL, Kimani E, Rollman BL, Paasche-Orlow MK, Bickmore TW. The Atrial Fibrillation Health Literacy Information Technology System: Pilot Assessment. JMIR Cardio. 2017;1(2):e7.

30. Orchard J, Neubeck L, Freedman B, Li J, Webster R, Zwar N, et al. eHealth Tools to Provide Structured Assistance for Atrial Fibrillation Screening, Management, and Guideline-Recommended Therapy in Metropolitan General Practice: The AF - SMART Study. J Am Heart Assoc. 2019;8(1):e010959.

31. K TH, A BB, Garan H, Sciacca RR, Riga T, Warren K, et al. Evaluating the Utility of mHealth ECG Heart Monitoring for the Detection and Management of Atrial Fibrillation in Clinical Practice. J Atr Fibrillation. 2017;9(5):1546.

32. Zado ES, Pammer M, Parham T, Lin D, Frankel DS, Dixit S, et al. "As Needed" nonvitamin K antagonist oral anticoagulants for infrequent atrial fibrillation episodes following atrial fibrillation ablation guided by diligent pulse monitoring: A feasibility study. J Cardiovasc Electrophysiol. 2019;30(5):631-8.

33. Stavrakis S, Stoner JA, Kardokus J, Garabelli PJ, Po SS, Lazzara R. Intermittent vs. Continuous Anticoagulation theRapy in patiEnts with Atrial Fibrillation (iCARE-AF): a randomized pilot study. J Interv Card Electrophysiol. 2017;48(1):51-60.

34. Caceres BA, Hickey KT, Bakken SB, Biviano AB, Garan H, Goldenthal IL, et al. Mobile Electrocardiogram Monitoring and Health-Related Quality of Life in Patients With Atrial Fibrillation: Findings From the iPhone Helping Evaluate Atrial Fibrillation Rhythm Through Technology (iHEART) Study. J Cardiovasc Nurs. 2020.

35. Kropp CM, Huber NL, Sager D, Tripp C, Burch A, Naniwadekar A, et al. Mobile-ECG screening in rural pharmacies: rates of atrial fibrillation and associated risk factors. Heart Lung. 2020.

36. Anderson JR, Hunter T, Dinallo JM, Glaser D, Roybal LK, Segovia A, et al. Population screening for atrial fibrillation by student pharmacists at health fairs. J Am Pharm Assoc (2003). 2020.

37. Goldenthal IL, Sciacca RR, Riga T, Bakken S, Baumeister M, Biviano AB, et al. Recurrent atrial fibrillation/flutter detection after ablation or cardioversion using the AliveCor KardiaMobile device: iHEART results. J Cardiovasc Electrophysiol. 2019;30(11):2220-8.

38. Rosenfeld LE, Amin AN, Hsu JC, Oxner A, Hills MT, Frankel DS. The Heart Rhythm Society/American College of Physicians Atrial Fibrillation Screening and Education Initiative. Heart Rhythm. 2019;16(8):e59-e65.

39. Orchard J, Freedman SB, Lowres N, Peiris D, Neubeck L. iPhone ECG screening by practice nurses and receptionists for atrial fibrillation in general practice: the GP-SEARCH qualitative pilot study. Aust Fam Physician. 2014;43(5):315-9.

40. Yan B, Tu H, Lam C, Swift C, Ho MS, Mok VCT, et al. Nurse Led Smartphone Electrographic Monitoring for Atrial Fibrillation after Ischemic Stroke: SPOT-AF. J Stroke. 2020;22(3):387-95.

41. Chen Y, Huang QF, Sheng CS, Zhang W, Shao S, Wang D, et al. Detection rate and treatment gap for atrial fibrillation identified through screening in community health centers in China (AF-CATCH): A prospective multicenter study. PLoS Med. 2020;17(7):e1003146.

42. Halcox JPJ, Wareham K, Cardew A, Gilmore M, Barry JP, Phillips C, et al. Assessment of Remote Heart Rhythm Sampling Using the AliveCor Heart Monitor to Screen for Atrial Fibrillation: the REHEARSE-AF Study. Circulation. 2017;136(19):1784‐94.

43. Grubb NR, Elder D, Broadhurst P, Reoch A, Tassie E, Neilson A. Atrial fibrillation case finding in over 65s with cardiovascular risk factors - Results of initial Scottish clinical experience. Int J Cardiol. 2019;288:94-9.

44. Wasserlauf J, You C, Patel R, Valys A, Albert D, Passman R. Smartwatch Performance for the Detection and Quantification of Atrial Fibrillation. Circ Arrhythm Electrophysiol. 2019;12(6):e006834.

45. William AD, Kanbour M, Callahan T, Bhargava M, Varma N, Rickard J, et al. Assessing the accuracy of an automated atrial fibrillation detection algorithm using smartphone technology: The iREAD Study. Heart Rhythm. 2018;15(10):1561-5.

46. Selder JL, Breukel L, Blok S, van Rossum AC, Tulevski, II, Allaart CP. A mobile one-lead ECG device incorporated in a symptom-driven remote arrhythmia monitoring program. The first 5,982 Hartwacht ECGs. Neth Heart J. 2019;27(1):38-45.

47. Tarakji KG, Wazni OM, Callahan T, Kanj M, Hakim AH, Wolski K, et al. Using a novel wireless system for monitoring patients after the atrial fibrillation ablation procedure: the iTransmit study. Heart Rhythm. 2015;12(3):554-9.

48. Orchard J, Lowres N, Freedman SB, Ladak L, Lee W, Zwar N, et al. Screening for atrial fibrillation during influenza vaccinations by primary care nurses using a smartphone electrocardiograph (iECG): A feasibility study. Eur J Prev Cardiol. 2016;23(2 suppl):13-20.

49. Zaprutko T, Zaprutko J, Baszko A, Sawicka D, Szalek A, Dymecka M, et al. Feasibility of Atrial Fibrillation Screening With Mobile Health Technologies at Pharmacies. J Cardiovasc Pharmacol Ther. 2020;25(2):142-51.

50. Chan NY, Choy CC, Chan CK, Siu CW. Effectiveness of a nongovernmental organization-led large-scale community atrial fibrillation screening program using the smartphone electrocardiogram: An observational cohort study. Heart Rhythm. 2018;15(9):1306-11.

51. Godin R, Yeung C, Baranchuk A, Guerra P, Healey JS. Screening for Atrial Fibrillation Using a Mobile, Single-Lead Electrocardiogram in Canadian Primary Care Clinics. Can J Cardiol. 2019;35(7):840-5.

52. Chan NY, Choy CC. Screening for atrial fibrillation in 13 122 Hong Kong citizens with smartphone electrocardiogram. Heart. 2017;103(1):24-31.

53. Soni A, Karna S, Fahey N, Sanghai S, Patel H, Raithatha S, et al. Age-and-sex stratified prevalence of atrial fibrillation in rural Western India: results of SMART-India, a population-based screening study. International journal of cardiology. 2019;280:84-8.

54. Gwynn J, Gwynne K, Rodrigues R, Thompson S, Bolton G, Dimitropoulos Y, et al. Atrial Fibrillation in Indigenous Australians: A Multisite Screening Study Using a Single-Lead ECG Device in Aboriginal Primary Health Settings. Heart Lung Circ. 2021;30(2):267-74.

55. Kim NR, Choi CK, Kim HS, Oh SH, Yang JH, Lee KH, et al. Screening for Atrial Fibrillation Using a Smartphone-Based Electrocardiogram in Korean Elderly. Chonnam Med J. 2020;56(1):50-4.

56. Andrade JG, Godin R, Nault I. Large-scale implementation of a pragmatic atrial fibrillation screening program in Canadian community practice. Pacing Clin Electrophysiol. 2020;43(7):768-9.

57. Lown M, Yue AM, Shah BN, Corbett SJ, Lewith G, Stuart B, et al. Screening for Atrial Fibrillation Using Economical and Accurate Technology (From the SAFETY Study). Am J Cardiol. 2018;122(8):1339-44.

58. Orchard JJ, Neubeck L, Freedman B, Webster R, Patel A, Gallagher R, et al. Atrial Fibrillation Screen, Management And Guideline Recommended Therapy (AF SMART II) in the rural primary care setting: an implementation study protocol. BMJ Open. 2018;8(10):e023130.

59. Savickas V, Stewart AJ, Rees-Roberts M, Short V, Bhamra SK, Corlett SA, et al. Opportunistic screening for atrial fibrillation by clinical pharmacists in UK general practice during the influenza vaccination season: A cross-sectional feasibility study. PLoS Med. 2020;17(7):e1003197.

60. Chan PH, Wong CK, Pun L, Wong YF, Wong MM, Chu DW, et al. Head-to-Head Comparison of the AliveCor Heart Monitor and Microlife WatchBP Office AFIB for Atrial Fibrillation Screening in a Primary Care Setting. Circulation. 2017;135(1):110-2.

61. Lowres N, Neubeck L, Salkeld G, Krass I, McLachlan AJ, Redfern J, et al. Feasibility and cost-effectiveness of stroke prevention through community screening for atrial fibrillation using iPhone ECG in pharmacies. The SEARCH-AF study. Thromb Haemost. 2014;111(6):1167-76.

62. Rajakariar K, Koshy AN, Sajeev JK, Nair S, Roberts L, Teh AW. Accuracy of a smartwatch based single-lead electrocardiogram device in detection of atrial fibrillation. Heart. 2020;106(9):665-70.

63. Evans GF, Shirk A, Muturi P, Soliman EZ. Feasibility of Using Mobile ECG Recording Technology to Detect Atrial Fibrillation in Low-Resource Settings. Glob Heart. 2017;12(4):285-9.

64. Rischard J, Waldmann V, Moulin T, Sharifzadehgan A, Lee R, Narayanan K, et al. Assessment of Heart Rhythm Disorders Using the AliveCor Heart Monitor: Beyond the Detection of Atrial Fibrillation. JACC Clin Electrophysiol. 2020;6(10):1313-5.

65. Koltowski L, Balsam P, Gllowczynska R, Rokicki JK, Peller M, Maksym J, et al. Kardia Mobile applicability in clinical practice: A comparison of Kardia Mobile and standard 12-lead electrocardiogram records in 100 consecutive patients of a tertiary cardiovascular care center. Cardiol J. 2019.

66. Wegner FK, Kochhauser S, Ellermann C, Lange PS, Frommeyer G, Leitz P, et al. Prospective blinded Evaluation of the smartphone-based AliveCor Kardia ECG monitor for Atrial Fibrillation detection: The PEAK-AF study. Eur J Intern Med. 2020;73:72-5.

67. Bumgarner JM, Lambert CT, Hussein AA, Cantillon DJ, Baranowski B, Wolski K, et al. Smartwatch Algorithm for Automated Detection of Atrial Fibrillation. J Am Coll Cardiol. 2018;71(21):2381-8.

68. Haberman ZC, Jahn RT, Bose R, Tun H, Shinbane JS, Doshi RN, et al. Wireless Smartphone ECG Enables Large-Scale Screening in Diverse Populations. J Cardiovasc Electrophysiol. 2015;26(5):520-6.

69. Gropler MRF, Dalal AS, Van Hare GF, Silva JNA. Can smartphone wireless ECGs be used to accurately assess ECG intervals in pediatrics? A comparison of mobile health monitoring to standard 12-lead ECG. PLoS One. 2018;13(9):e0204403.

70. Cunha S, Antunes E, Antoniou S, Tiago S, Relvas R, Fernandez-Llimos F, et al. Raising awareness and early detection of atrial fibrillation, an experience resorting to mobile technology centred on informed individuals. Res Social Adm Pharm. 2019.

71. Lowres N, Mulcahy G, Gallagher R, Ben Freedman S, Marshman D, Kirkness A, et al. Self-monitoring for atrial fibrillation recurrence in the discharge period post-cardiac surgery using an iPhone electrocardiogram. Eur J Cardiothorac Surg. 2016;50(1):44-51.

72. Williams J, Pearce K, Benett I, Williams J, Manchester M, Pearce K, et al. The effectiveness of a mobile ECG device in identifying AF: sensitivity, specificity and predictive value. Br J Cardiol. 2015;22(2):70-2.

73. Hermans ANL, Gawalko M, Pluymaekers N, Dinh T, Weijs B, van Mourik MJW, et al. Long-term intermittent versus short continuous heart rhythm monitoring for the detection of atrial fibrillation recurrences after catheter ablation. Int J Cardiol. 2021.

74. Reed MJ, Muir A, Cullen J, Murphy R, Pollard V, Zangana G, et al. Establishing a Smartphone Ambulatory ECG Service for Patients Presenting to the Emergency Department with Pre-Syncope and Palpitations. Medicina (Kaunas). 2021;57(2).

75. Reed MJ, Grubb NR, Lang CC, O'Brien R, Simpson K, Padarenga M, et al. Multi-centre Randomised Controlled Trial of a Smartphone-based Event Recorder Alongside Standard Care Versus Standard Care for Patients Presenting to the Emergency Department with Palpitations and Pre-syncope: The IPED (Investigation of Palpitations in the ED) study. EClinicalMedicine. 2019;8:37-46.

76. Chen YH, Hung CS, Huang CC, Hung YC, Hwang JJ, Ho YL. Atrial Fibrillation Screening in Nonmetropolitan Areas Using a Telehealth Surveillance System With an Embedded Cloud-Computing Algorithm: Prospective Pilot Study. JMIR Mhealth Uhealth. 2017;5(9):e135.

77. Svennberg E, Engdahl J, Al-Khalili F, Friberg L, Frykman V, Rosenqvist M. Mass Screening for Untreated Atrial Fibrillation: The STROKESTOP Study. Circulation. 2015;131(25):2176-84.

78. Olsson AS, Engdahl J. Detection of Atrial Fibrillation with Intermittent Handheld Electrocardiogram in Patients with Ischemic Stroke and Transient Ischemic Attack. J Stroke Cerebrovasc Dis. 2016;25(11):2648-52.

79. Kemp Gudmundsdottir K, Fredriksson T, Svennberg E, Al-Khalili F, Friberg L, Frykman V, et al. Stepwise mass screening for atrial fibrillation using N-terminal B-type natriuretic peptide: the STROKESTOP II study. Europace. 2020;22(1):24-32.

80. Anczykowski J, Willems S, Hoffmann BA, Meinertz T, Blankenberg S, Patten M. Early Detection of Symptomatic Paroxysmal Cardiac Arrhythmias by Trans-Telephonic ECG Monitoring: Impact on Diagnosis and Treatment of Atrial Fibrillation. J Cardiovasc Electrophysiol. 2016;27(9):1032-7.

81. Busch MC, Gross S, Alte D, Kors JA, Völzke H, Ittermann T, et al. Impact of atrial fibrillation detected by extended monitoring—A population‐based cohort study. Annals of Noninvasive Electrocardiology. 2017;22(6):e12453.

82. Liu J, Fang PH, Hou Y, Li XF, Liu Y, Wang YS, et al. The value of transtelephonic electrocardiogram monitoring system during the "Blanking Period" after ablation of atrial fibrillation. J Electrocardiol. 2010;43(6):667-72.

83. Vukajlovic D, Gussak I, George S, Simic G, Bojovic B, Hadzievski L, et al. Wireless monitoring of reconstructed 12-lead ECG in atrial fibrillation patients enables differential diagnosis of recurrent arrhythmias. Annu Int Conf IEEE Eng Med Biol Soc. 2011;2011:4741-4.

84. Gussak I, Vukajlovic D, Vukcevic V, George S, Bojovic B, Hadzievski L, et al. Wireless remote monitoring of reconstructed 12-lead ECGs after ablation for atrial fibrillation using a hand-held device. J Electrocardiol. 2012;45(2):129-35.

85. Aljuaid M, Marashly Q, AlDanaf J, Tawhari I, Barakat M, Barakat R, et al. Smartphone ECG monitoring system helps lower emergency room and clinic visits in post–atrial fibrillation ablation patients. Clinical Medicine Insights: Cardiology. 2020;14:1179546820901508.

86. Pluymaekers NA, Dudink EA, Luermans JG, Meeder JG, Lenderink T, Widdershoven J, et al. Early or delayed cardioversion in recent-onset atrial fibrillation. New England Journal of Medicine. 2019;380(16):1499-508.

87. Desteghe L, Raymaekers Z, Lutin M, Vijgen J, Dilling-Boer D, Koopman P, et al. Performance of handheld electrocardiogram devices to detect atrial fibrillation in a cardiology and geriatric ward setting. Ep Europace. 2017;19(1):29-39.

88. Lowres N, Mulcahy G, Gallagher R, Ben Freedman S, Marshman D, Kirkness A, et al. Self-monitoring for atrial fibrillation recurrence in the discharge period post-cardiac surgery using an iPhone electrocardiogram. European Journal of Cardio-Thoracic Surgery. 2016;50(1):44-51.

89. Jaakkola J, Jaakkola S, Lahdenoja O, Hurnanen T, Koivisto T, Pänkäälä M, et al. Mobile phone detection of atrial fibrillation with mechanocardiography: The mode-af study (mobile phone detection of atrial fibrillation). Circulation. 2018;137(14):1524-7.

90. Perez MV, Mahaffey KW, Hedlin H, Rumsfeld JS, Garcia A, Ferris T, et al. Large-Scale Assessment of a Smartwatch to Identify Atrial Fibrillation. N Engl J Med. 2019;381(20):1909-17.

91. Guo Y, Wang H, Zhang H, Liu T, Liang Z, Xia Y, et al. Mobile Photoplethysmographic Technology to Detect Atrial Fibrillation. J Am Coll Cardiol. 2019;74(19):2365-75.

92. Inui T, Kohno H, Kawasaki Y, Matsuura K, Ueda H, Tamura Y, et al. Use of a Smart Watch for Early Detection of Paroxysmal Atrial Fibrillation: Validation Study. JMIR Cardio. 2020;4(1):e14857.

93. Hochstadt A, Chorin E, Viskin S, Schwartz AL, Lubman N, Rosso R. Continuous heart rate monitoring for automatic detection of atrial fibrillation with novel bio-sensing technology. J Electrocardiol. 2019;52:23-7.

94. Eerikäinen LM, Dekker L, Bonomi AG, Vullings R, Schipper F, Margarito J, et al., editors. Validating features for atrial fibrillation detection from photoplethysmogram under hospital and free-living conditions. 2017 Computing in Cardiology (CinC); 2017: IEEE.

95. Bonomi AG, Schipper F, Eerikäinen LM, Margarito J, van Dinther R, Muesch G, et al. Atrial Fibrillation Detection Using a Novel Cardiac Ambulatory Monitor Based on Photo-Plethysmography at the Wrist. J Am Heart Assoc. 2018;7(15):e009351.

96. Nemati S, Ghassemi MM, Ambai V, Isakadze N, Levantsevych O, Shah A, et al. Monitoring and detecting atrial fibrillation using wearable technology. Annu Int Conf IEEE Eng Med Biol Soc. 2016;2016:3394-7.

97. Zhang H, Zhang J, Li HB, Chen YX, Yang B, Guo YT, et al. Validation of Single Centre Pre-Mobile Atrial Fibrillation Apps for Continuous Monitoring of Atrial Fibrillation in a Real-World Setting: Pilot Cohort Study. J Med Internet Res. 2019;21(12):e14909.

98. Tison GH, Sanchez JM, Ballinger B, Singh A, Olgin JE, Pletcher MJ, et al. Passive Detection of Atrial Fibrillation Using a Commercially Available Smartwatch. JAMA Cardiol. 2018;3(5):409-16.

99. Chen E, Jiang J, Su R, Gao M, Zhu S, Zhou J, et al. A new smart wristband equipped with an artificial intelligence algorithm to detect atrial fibrillation. Heart Rhythm. 2020;17(5 Pt B):847-53.

100. Selder JL, Proesmans T, Breukel L, Dur O, Gielen W, van Rossum AC, et al. Assessment of a standalone photoplethysmography (PPG) algorithm for detection of atrial fibrillation on wristband-derived data. Comput Methods Programs Biomed. 2020;197:105753.

101. Corino VDA, Laureanti R, Ferranti L, Scarpini G, Lombardi F, Mainardi LT. Detection of atrial fibrillation episodes using a wristband device. Physiol Meas. 2017;38(5):787-99.

102. Kwon S, Hong J, Choi EK, Lee B, Baik C, Lee E, et al. Detection of Atrial Fibrillation Using a Ring-Type Wearable Device (CardioTracker) and Deep Learning Analysis of Photoplethysmography Signals: Prospective Observational Proof-of-Concept Study. J Med Internet Res. 2020;22(5):e16443.

103. Jacobsen M, Dembek TA, Ziakos AP, Gholamipoor R, Kobbe G, Kollmann M, et al. Reliable Detection of Atrial Fibrillation with a Medical Wearable during Inpatient Conditions. Sensors (Basel). 2020;20(19).

104. Conroy T, Guzman JH, Hall B, Tsouri G, Couderc JP. Detection of atrial fibrillation using an earlobe photoplethysmographic sensor. Physiol Meas. 2017;38(10):1906-18.

105. Dörr M, Nohturfft V, Brasier N, Bosshard E, Djurdjevic A, Gross S, et al. The WATCH AF Trial: SmartWATCHes for Detection of Atrial Fibrillation. JACC Clin Electrophysiol. 2019;5(2):199-208.

106. Koshy AN, Sajeev JK, Nerlekar N, Brown AJ, Rajakariar K, Zureik M, et al. Smart watches for heart rate assessment in atrial arrhythmias. Int J Cardiol. 2018;266:124-7.

107. Huynh P, Shan R, Osuji N, Ding J, Marvel F, Sharma G, et al. ACCURACY OF APPLE WATCH HEART RATE MEASUREMENTS IN PATIENTS WITH OBSTRUCTIVE SLEEP APNEA AND ATRIAL FIBRILLATION. Journal of the American College of Cardiology. 2020;75(11_Supplement_1):3563-.

108. Seshadri DR, Bittel B, Browsky D, Houghtaling P, Drummond CK, Desai M, et al. Accuracy of the Apple Watch 4 to Measure Heart Rate in Patients With Atrial Fibrillation. IEEE J Transl Eng Health Med. 2020;8:2700204.

109. Al-Kaisey AM, Koshy AN, Ha FJ, Spencer R, Toner L, Sajeev JK, et al. Accuracy of wrist-worn heart rate monitors for rate control assessment in atrial fibrillation. Int J Cardiol. 2020;300:161-4.

110. Huynh P, Shan R, Osuji N, Ding J, Isakadze N, Marvel FA, et al. Heart Rate Measurements in Patients with Obstructive Sleep Apnea and Atrial Fibrillation: Prospective Pilot Study Assessing Apple Watch's Agreement With Telemetry Data. JMIR Cardio. 2021;5(1):e18050.

111. Turakhia MP, Ullal AJ, Hoang DD, Than CT, Miller JD, Friday KJ, et al. Feasibility of extended ambulatory electrocardiogram monitoring to identify silent atrial fibrillation in high-risk patients: the Screening Study for Undiagnosed Atrial Fibrillation (STUDY-AF). Clin Cardiol. 2015;38(5):285-92.

112. Heo NJ, Rhee SY, Waalen J, Steinhubl S. Chronic kidney disease and undiagnosed atrial fibrillation in individuals with diabetes. Cardiovasc Diabetol. 2020;19(1):157.

113. Steinhubl SR, Waalen J, Edwards AM, Ariniello LM, Mehta RR, Ebner GS, et al. Effect of a Home-Based Wearable Continuous ECG Monitoring Patch on Detection of Undiagnosed Atrial Fibrillation: The mSToPS Randomized Clinical Trial. Jama. 2018;320(2):146-55.

114. Wineinger NE, Barrett PM, Zhang Y, Irfanullah I, Muse ED, Steinhubl SR, et al. Identification of paroxysmal atrial fibrillation subtypes in over 13,000 individuals. Heart Rhythm. 2019;16(1):26-30.

115. Sabar MI, Ara F, Henderson A, Ahmed O, Potter C, John I, et al. A study to assess a novel automated electrocardiogram technology in screening for atrial fibrillation. Pacing Clin Electrophysiol. 2019;42(10):1383-9.

116. Reverberi C, Rabia G, De Rosa F, Bosi D, Botti A, Benatti G. The RITMIA™ Smartphone App for Automated Detection of Atrial Fibrillation: Accuracy in Consecutive Patients Undergoing Elective Electrical Cardioversion. Biomed Res Int. 2019;2019:4861951.

117. Lin CT, Chang KC, Lin CL, Chiang CC, Lu SW, Chang SS, et al. An intelligent telecardiology system using a wearable and wireless ECG to detect atrial fibrillation. IEEE Trans Inf Technol Biomed. 2010;14(3):726-33.

118. Kimura T, Aizawa Y, Kurata N, Nakajima K, Kashimura S, Kunitomi A, et al. Assessment of atrial fibrillation ablation outcomes with clinic ECG, monthly 24-h Holter ECG, and twice-daily telemonitoring ECG. Heart Vessels. 2017;32(3):317-25.

119. Brunetti ND, De Gennaro L, Pellegrino PL, Dellegrottaglie G, Antonelli G, Di Biase M. Atrial fibrillation with symptoms other than palpitations: incremental diagnostic sensitivity with at-home tele-cardiology assessment for emergency medical service. Eur J Prev Cardiol. 2012;19(3):306-13.

120. Wu CF, Yang CY, Li AH, Chuang WP, Chen KC, Liu YH, et al. Detection of asymptomatic paroxysmal atrial fibrillation with the trans-telephonic electrocardiograph system. Telemed J E Health. 2012;18(3):193-7.

121. Scalvini S, Piepoli M, Zanelli E, Volterrani M, Giordano A, Glisenti F. Incidence of atrial fibrillation in an Italian population followed by their GPs through a telecardiology service. Int J Cardiol. 2005;98(2):215-20.

122. Atarashi H, Ogawa S, Inoue H, Hamada C. Dose-response effect of flecainide in patients with symptomatic paroxysmal atrial fibrillation and/or flutter monitored with trans-telephonic electrocardiography: a multicenter, placebo-controlled, double-blind trial. Circulation journal. 2007;71(3):294‐300.

123. Omboni S, Verberk WJ. Opportunistic screening of atrial fibrillation by automatic blood pressure measurement in the community. BMJ Open. 2016;6(4):e010745.

124. Wiesel J, Abraham S, Messineo FC. Screening for asymptomatic atrial fibrillation while monitoring the blood pressure at home: trial of regular versus irregular pulse for prevention of stroke (TRIPPS 2.0). Am J Cardiol. 2013;111(11):1598-601.

125. Wiesel J, Wiesel D, Suri R, Messineo FC. The use of a modified sphygmomanometer to detect atrial fibrillation in outpatients. Pacing Clin Electrophysiol. 2004;27(5):639-43.

126. Wiesel J, Fitzig L, Herschman Y, Messineo FC. Detection of atrial fibrillation using a modified microlife blood pressure monitor. Am J Hypertens. 2009;22(8):848-52.

127. Stergiou GS, Karpettas N, Protogerou A, Nasothimiou EG, Kyriakidis M. Diagnostic accuracy of a home blood pressure monitor to detect atrial fibrillation. J Hum Hypertens. 2009;23(10):654-8.

128. Kearley K, Selwood M, Van den Bruel A, Thompson M, Mant D, Hobbs FR, et al. Triage tests for identifying atrial fibrillation in primary care: a diagnostic accuracy study comparing single-lead ECG and modified BP monitors. BMJ Open. 2014;4(5):e004565.

129. Gandolfo C, Balestrino M, Bruno C, Finocchi C, Reale N. Validation of a simple method for atrial fibrillation screening in patients with stroke. Neurol Sci. 2015;36(9):1675-8.

130. Marazzi G, Iellamo F, Volterrani M, Lombardo M, Pelliccia F, Righi D, et al. Comparison of Microlife BP A200 Plus and Omron M6 blood pressure monitors to detect atrial fibrillation in hypertensive patients. Adv Ther. 2012;29(1):64-70.

131. Asaithambi G, Monita JE, Annamalai MR, Ho BM, Marino EH, Hanson SK. Prevalence of atrial fibrillation with insertable cardiac monitors in cryptogenic stroke: A single-center experience. J Electrocardiol. 2018;51(6):973-6.

132. Bertelsen L, Diederichsen SZ, Haugan KJ, Brandes A, Graff C, Krieger D, et al. Left Atrial Late Gadolinium Enhancement is Associated With Incident Atrial Fibrillation as Detected by Continuous Monitoring With Implantable Loop Recorders. JACC Cardiovasc Imaging. 2020;13(8):1690-700.

133. Carrazco C, Golyan D, Kahen M, Black K, Libman RB, Katz JM. Prevalence and Risk Factors for Paroxysmal Atrial Fibrillation and Flutter Detection after Cryptogenic Ischemic Stroke. J Stroke Cerebrovasc Dis. 2018;27(1):203-9.

134. Chorin E, Peterson C, Kogan E, Barbhaiya C, Aizer A, Holmes D, et al. Comparison of the Effect of Atrial Fibrillation Detection Algorithms in Patients With Cryptogenic Stroke Using Implantable Loop Recorders. Am J Cardiol. 2020;129:25-9.

135. Dekker LR, Pokushalov E, Sanders P, Lindborg KA, Maus B, Purerfellner H. Continuous Cardiac Monitoring around Atrial Fibrillation Ablation: Insights on Clinical Classifications and End Points. Pacing Clin Electrophysiol. 2016;39(8):805-13.

136. De With RR, Erkuner O, Rienstra M, Nguyen BO, Korver FWJ, Linz D, et al. Temporal patterns and short-term progression of paroxysmal atrial fibrillation: data from RACE V. Europace. 2020;22(8):1162-72.

137. Diederichsen SZ, Haugan KJ, Brandes A, Graff C, Krieger D, Kronborg C, et al. Incidence and predictors of atrial fibrillation episodes as detected by implantable loop recorder in patients at risk: From the LOOP study. Am Heart J. 2020;219:117-27.

138. Forkmann M, Schwab C, Edler D, Vevecka A, Butz S, Haller B, et al. Characteristics of early recurrences detected by continuous cardiac monitoring influencing the long-term outcome after atrial fibrillation ablation. J Cardiovasc Electrophysiol. 2019;30(10):1886-93.

139. Haldar S, Khan HR, Boyalla V, Kralj-Hans I, Jones S, Lord J, et al. Catheter ablation vs. thoracoscopic surgical ablation in long-standing persistent atrial fibrillation: CASA-AF randomized controlled trial. Eur Heart J. 2020.

140. Israel C, Kitsiou A, Kalyani M, Deelawar S, Ejangue LE, Rogalewski A, et al. Detection of atrial fibrillation in patients with embolic stroke of undetermined source by prolonged monitoring with implantable loop recorders. Thromb Haemost. 2017;117(10):1962-9.

141. Kusiak A, Jastrzebski M, Bednarski A, Kulakowski P, Piotrowski R, Kozluk E, et al. Diagnostic value of implantable loop recorder in patients undergoing cryoballoon ablation of atrial fibrillation. Ann Noninvasive Electrocardiol. 2020;25(4):e12733.

142. Marks D, Ho R, Then R, Weinstock JL, Teklemariam E, Kakadia B, et al. Real-world experience with implantable loop recorder monitoring to detect subclinical atrial fibrillation in patients with cryptogenic stroke: The value of p wave dispersion in predicting arrhythmia occurrence. Int J Cardiol. 2020.

143. Nasir JM, Pomeroy W, Marler A, Hann M, Baykaner T, Jones R, et al. Predicting Determinants of Atrial Fibrillation or Flutter for Therapy Elucidation in Patients at Risk for Thromboembolic Events (PREDATE AF) Study. Heart Rhythm. 2017;14(7):955-61.

144. Pedersen KB, Madsen C, Sandgaard NCF, Diederichsen ACP, Bak S, Brandes A. Subclinical atrial fibrillation in patients with recent transient ischemic attack. J Cardiovasc Electrophysiol. 2018;29(5):707-14.

145. Poli S, Diedler J, Hartig F, Gotz N, Bauer A, Sachse T, et al. Insertable cardiac monitors after cryptogenic stroke--a risk factor based approach to enhance the detection rate for paroxysmal atrial fibrillation. Eur J Neurol. 2016;23(2):375-81.

146. Prabhu S, Taylor AJ, Costello BT, Kaye DM, McLellan AJA, Voskoboinik A, et al. Catheter Ablation Versus Medical Rate Control in Atrial Fibrillation and Systolic Dysfunction: The CAMERA-MRI Study. J Am Coll Cardiol. 2017;70(16):1949-61.

147. Purerfellner H, Sanders P, Sarkar S, Reisfeld E, Reiland J, Koehler J, et al. Adapting detection sensitivity based on evidence of irregular sinus arrhythmia to improve atrial fibrillation detection in insertable cardiac monitors. Europace. 2018;20(FI_3):f321-f8.

148. Reiffel JA, Verma A, Kowey PR, Halperin JL, Gersh BJ, Wachter R, et al. Incidence of Previously Undiagnosed Atrial Fibrillation Using Insertable Cardiac Monitors in a High-Risk Population: The REVEAL AF Study. JAMA Cardiol. 2017;2(10):1120-7.

149. Sanders P, Purerfellner H, Pokushalov E, Sarkar S, Di Bacco M, Maus B, et al. Performance of a new atrial fibrillation detection algorithm in a miniaturized insertable cardiac monitor: Results from the Reveal LINQ Usability Study. Heart Rhythm. 2016;13(7):1425-30.

150. Seow SC, How AK, Chan SP, Teoh HL, Lim TW, Singh D, et al. High Incidence of Occult Atrial Fibrillation in Asian Patients with Cryptogenic Stroke. J Stroke Cerebrovasc Dis. 2018;27(8):2182-6.

151. Victor CU, Carolina PE, Jorge TR, Joaquin CR, Manuel SG, Marta CM, et al. Incidence and Predictive Factors of Hidden Atrial Fibrillation Detected by Implantable Loop Recorder After an Embolic Stroke of Undetermined Source. J Atr Fibrillation. 2018;11(3):2078.

152. Watson RA, Wellings J, Hingorani R, Zhan T, Frisch DR, Ho RT, et al. Atrial fibrillation post central retinal artery occlusion: Role of implantable loop recorders. Pacing Clin Electrophysiol. 2020.

153. Wechselberger S, Kronborg M, Huo Y, Piorkowski J, Neudeck S, Passler E, et al. Continuous monitoring after atrial fibrillation ablation: the LINQ AF study. Europace. 2018;20(FI_3):f312-f20.

154. Xu J, Sethi P, Biby S, Allred J, Seiler A, Sabir R. Predictors of atrial fibrillation detection and features of recurrent strokes in patients after cryptogenic stroke. J Stroke Cerebrovasc Dis. 2020;29(9):104934.

155. Ziegler PD, Rogers JD, Ferreira SW, Nichols AJ, Richards M, Koehler JL, et al. Long-term detection of atrial fibrillation with insertable cardiac monitors in a real-world cryptogenic stroke population. Int J Cardiol. 2017;244:175-9.

156. Ziegler PD, Rogers JD, Ferreira SW, Nichols AJ, Sarkar S, Koehler JL, et al. Real-World Experience with Insertable Cardiac Monitors to Find Atrial Fibrillation in Cryptogenic Stroke. Cerebrovasc Dis. 2015;40(3-4):175-81.

157. Bergau L, Sohns C, Sossalla S, Munoz-Exposito P, Luethje L, Zabel M. Submuscular implantation of insertable cardiac monitors improves the reliability of detection of atrial fibrillation. J Interv Card Electrophysiol. 2015;42(2):143-9.

158. Choe WC, Passman RS, Brachmann J, Morillo CA, Sanna T, Bernstein RA, et al. A Comparison of Atrial Fibrillation Monitoring Strategies After Cryptogenic Stroke (from the Cryptogenic Stroke and Underlying AF Trial). Am J Cardiol. 2015;116(6):889-93.

159. Cotter PE, Martin PJ, Ring L, Warburton EA, Belham M, Pugh PJ. Incidence of atrial fibrillation detected by implantable loop recorders in unexplained stroke. Neurology. 2013;80(17):1546-50.

160. Dion F, Saudeau D, Bonnaud I, Friocourt P, Bonneau A, Poret P, et al. Unexpected low prevalence of atrial fibrillation in cryptogenic ischemic stroke: a prospective study. J Interv Card Electrophysiol. 2010;28(2):101-7.

161. Etgen T, Hochreiter M, Mundel M, Freudenberger T. Insertable cardiac event recorder in detection of atrial fibrillation after cryptogenic stroke: an audit report. Stroke. 2013;44(7):2007-9.

162. Hindricks G, Pokushalov E, Urban L, Taborsky M, Kuck KH, Lebedev D, et al. Performance of a new leadless implantable cardiac monitor in detecting and quantifying atrial fibrillation: Results of the XPECT trial. Circ Arrhythm Electrophysiol. 2010;3(2):141-7.

163. Jorfida M, Antolini M, Cerrato E, Caprioli MG, Castagno D, Garrone P, et al. Cryptogenic ischemic stroke and prevalence of asymptomatic atrial fibrillation: a prospective study. J Cardiovasc Med (Hagerstown). 2016;17(12):863-9.

164. Merce J, Garcia M, Ustrell X, Pellise A, de Castro R, Bardaji A. Implantable loop recorder: a new tool in the diagnosis of cryptogenic stroke. Rev Esp Cardiol (Engl Ed). 2013;66(8):665-6.

165. Muller P, Ivanov V, Kara K, Klein-Wiele O, Forkmann M, Piorkowski C, et al. Total atrial conduction time to predict occult atrial fibrillation after cryptogenic stroke. Clin Res Cardiol. 2017;106(2):113-9.

166. Reinke F, Bettin M, Ross LS, Kochhauser S, Kleffner I, Ritter M, et al. Refinement of detecting atrial fibrillation in stroke patients: results from the TRACK-AF Study. Eur J Neurol. 2018;25(4):631-6.

167. Ritter MA, Kochhauser S, Duning T, Reinke F, Pott C, Dechering DG, et al. Occult atrial fibrillation in cryptogenic stroke: detection by 7-day electrocardiogram versus implantable cardiac monitors. Stroke. 2013;44(5):1449-52.

168. Sanna T, Diener HC, Passman RS, Di Lazzaro V, Bernstein RA, Morillo CA, et al. Cryptogenic stroke and underlying atrial fibrillation. N Engl J Med. 2014;370(26):2478-86.

169. Yaeger A, Keenan BT, Cash NR, Parham T, Deo R, Frankel DS, et al. Impact of a nurse-led limited risk factor modification program on arrhythmia outcomes in patients with atrial fibrillation undergoing catheter ablation. J Cardiovasc Electrophysiol. 2020;31(2):423-31.

170. Christensen LM, Krieger DW, Hojberg S, Pedersen OD, Karlsen FM, Jacobsen MD, et al. Paroxysmal atrial fibrillation occurs often in cryptogenic ischaemic stroke. Final results from the SURPRISE study. Eur J Neurol. 2014;21(6):884-9.

171. Romanov A, Pokushalov E, Ponomarev D, Bayramova S, Shabanov V, Losik D, et al. Long-term suppression of atrial fibrillation by botulinum toxin injection into epicardial fat pads in patients undergoing cardiac surgery: Three-year follow-up of a randomized study. Heart Rhythm. 2019;16(2):172-7.

172. Kitsiou A, Rogalewski A, Kalyani M, Deelawar S, Tribunyan S, Greeve I, et al. Atrial fibrillation in patients with embolic stroke of undetermined source during 3 years of prolonged monitoring with an implantable loop recorder. Thromb Haemost. 2021.

173. Ciconte G, Saviano M, Giannelli L, Calovic Z, Baldi M, Ciaccio C, et al. Atrial fibrillation detection using a novel three-vector cardiac implantable monitor: the atrial fibrillation detect study. Europace. 2017;19(7):1101-8.

174. Lacour P, Dang PL, Huemer M, Parwani AS, Attanasio P, Pieske B, et al. Performance of the New BioMonitor 2-AF Insertable Cardiac Monitoring System: Can Better be Worse? Pacing Clin Electrophysiol. 2017;40(5):516-26.

175. Lauschke J, Busch M, Haverkamp W, Bulava A, Schneider R, Andresen D, et al. New implantable cardiac monitor with three-lead ECG and active noise detection. Herz. 2017;42(6):585-92.

176. Healey JS, Alings M, Ha A, Leong-Sit P, Birnie DH, de Graaf JJ, et al. Subclinical Atrial Fibrillation in Older Patients. Circulation. 2017;136(14):1276-83.

177. Nolker G, Mayer J, Boldt LH, Seidl K, V VAND, Massa T, et al. Performance of an Implantable Cardiac Monitor to Detect Atrial Fibrillation: Results of the DETECT AF Study. J Cardiovasc Electrophysiol. 2016;27(12):1403-10.

178. The Majority of Americans’ Mobile Time Spent Takes Place in Apps. <https://www.emarketer.com/content/the-majority-of-americans-mobile-time-spent-takes-place-in-apps> Accessed on 30th of the November 2020.

179. The Growing Value of Digital Health. Evidence and Impact on Human Health and the Healthcare System Institute Report. <https://www.iqvia.com/insights/the-iqvia-institute/reports/the-growing-value-of-digital-health> Accessed on 30th of the November 2020.

180. Turchioe MR, Jimenez V, Isaac S, Alshalabi M, Slotwiner D, Creber RM. Review of mobile applications for the detection and management of atrial fibrillation. Heart Rhythm O2. 2020;1(1):35-43.

181. Desteghe L, Kluts K, Vijgen J, Koopman P, Dilling-Boer D, Schurmans J, et al. The Health Buddies App as a Novel Tool to Improve Adherence and Knowledge in Atrial Fibrillation Patients: A Pilot Study. JMIR Mhealth Uhealth. 2017;5(7):e98.

182. Ghanbari H, Ansari S, Ghannam M, Lathkar-Pradhan S, Kratz A, Oral H, et al. Feasibility and Usability of a Mobile Application to Assess Symptoms and Affect in Patients with Atrial Fibrillation: A Pilot Study. J Atr Fibrillation. 2017;10(2):1672.

183. Guo Y, Chen Y, Lane DA, Liu L, Wang Y, Lip GYH. Mobile Health Technology for Atrial Fibrillation Management Integrating Decision Support, Education, and Patient Involvement: mAF App Trial. Am J Med. 2017;130(12):1388-96 e6.

184. Hirschey J, Bane S, Mansour M, Sperber J, Agboola S, Kvedar J, et al. Evaluating the Usability and Usefulness of a Mobile App for Atrial Fibrillation Using Qualitative Methods: Exploratory Pilot Study. JMIR Hum Factors. 2018;5(1):e13.

185. Manimaran M, Das D, Martinez P, Schwartz R, Schilling R, Finlay M. The impact of virtual arrhythmia clinics following catheter ablation for atrial fibrillation. Eur Heart J Qual Care Clin Outcomes. 2019;5(3):272-3.

186. Stephan LS, Almeida ED, Guimaraes RB, Ley AG, Mathias RG, Assis MV, et al. Oral Anticoagulation in Atrial Fibrillation: Development and Evaluation of a Mobile Health Application to Support Shared Decision-Making. Arq Bras Cardiol. 2018;110(1):7-15.

187. Guo Y, Lane DA, Wang L, Zhang H, Wang H, Zhang W, et al. Mobile Health Technology to Improve Care for Patients With Atrial Fibrillation. J Am Coll Cardiol. 2020;75(13):1523-34.

188. Guo Y, Lane DA, Chen Y, Lip GYH, m AFAIITi. Regular Bleeding Risk Assessment Associated with Reduction in Bleeding Outcomes: The mAFA-II Randomized Trial. Am J Med. 2020;133(10):1195-202 e2.

189. Guo Y, Guo J, Shi X, Yao Y, Sun Y, Xia Y, et al. Mobile health technology-supported atrial fibrillation screening and integrated care: A report from the mAFA-II trial Long-term Extension Cohort. Eur J Intern Med. 2020.

190. Balsam P, Borodzicz S, Malesa K, Puchta D, Tyminska A, Ozieranski K, et al. OCULUS study: Virtual reality-based education in daily clinical practice. Cardiol J. 2019;26(3):260-4.

191. Desteghe L, Germeys J, Vijgen J, Koopman P, Dilling-Boer D, Schurmans J, et al. Effectiveness and usability of an online tailored education platform for atrial fibrillation patients undergoing a direct current cardioversion or pulmonary vein isolation. Int J Cardiol. 2018;272:123-9.

192. Guhl E, Althouse AD, Pusateri AM, Kimani E, Paasche-Orlow MK, Bickmore TW, et al. The Atrial Fibrillation Health Literacy Information Technology Trial: Pilot Trial of a Mobile Health App for Atrial Fibrillation. JMIR Cardio. 2020;4(1):e17162.

193. Carter L, Gardner M, Magee K, Fearon A, Morgulis I, Doucette S, et al. An Integrated Management Approach to Atrial Fibrillation. J Am Heart Assoc. 2016;5(1).

194. Ferguson C, Hickman LD, Phillips J, Newton PJ, Inglis SC, Lam L, et al. An mHealth intervention to improve nurses' atrial fibrillation and anticoagulation knowledge and practice: the EVICOAG study. Eur J Cardiovasc Nurs. 2019;18(1):7-15.

195. Mesquita J, Maniar N, Baykaner T, Rogers AJ, Swerdlow M, Alhusseini MI, et al. Online webinar training to analyse complex atrial fibrillation maps: A randomized trial. PLoS One. 2019;14(7):e0217988.

196. Goni L, de la OV, Barrio-López MT, Ramos P, Tercedor L, Ibañez-Criado JL, et al. A Remote Nutritional Intervention to Change the Dietary Habits of Patients Undergoing Ablation of Atrial Fibrillation: Randomized Controlled Trial. J Med Internet Res. 2020;22(12):e21436.

197. Zoppo F, Facchin D, Molon G, Zanotto G, Catanzariti D, Rossillo A, et al. Improving atrial fibrillation detection in patients with implantable cardiac devices by means of a remote monitoring and management application. Pacing Clin Electrophysiol. 2014;37(12):1610-8.

198. Cox JL, Parkash R, Foster GA, Xie F, MacKillop JH, Ciaccia A, et al. Integrated Management Program Advancing Community Treatment of Atrial Fibrillation (IMPACT-AF): A cluster randomized trial of a computerized clinical decision support tool. Am Heart J. 2020;224:35-46.

199. Eckman MH, Costea A, Attari M, Munjal J, Wise RE, Knochelmann C, et al. Shared decision-making tool for thromboprophylaxis in atrial fibrillation - A feasibility study. Am Heart J. 2018;199:13-21.

200. Eckman MH, Lip GY, Wise RE, Speer B, Sullivan M, Walker N, et al. Impact of an Atrial Fibrillation Decision Support Tool on thromboprophylaxis for atrial fibrillation. Am Heart J. 2016;176:17-27.

201. Hendriks JM, de Wit R, Crijns HJ, Vrijhoef HJ, Prins MH, Pisters R, et al. Nurse-led care vs. usual care for patients with atrial fibrillation: results of a randomized trial of integrated chronic care vs. routine clinical care in ambulatory patients with atrial fibrillation. Eur Heart J. 2012;33(21):2692-9.

202. Karlsson LO, Nilsson S, Bang M, Nilsson L, Charitakis E, Janzon M. A clinical decision support tool for improving adherence to guidelines on anticoagulant therapy in patients with atrial fibrillation at risk of stroke: A cluster-randomized trial in a Swedish primary care setting (the CDS-AF study). PLoS Med. 2018;15(3):e1002528.

203. Rosier A, Mabo P, Temal L, Van Hille P, Dameron O, Deleger L, et al. Personalized and automated remote monitoring of atrial fibrillation. Europace. 2016;18(3):347-52.

204. Sheibani R, Sheibani M, Heidari-Bakavoli A, Abu-Hanna A, Eslami S. The Effect of a Clinical Decision Support System on Improving Adherence to Guideline in the Treatment of Atrial Fibrillation: An Interrupted Time Series Study. J Med Syst. 2017;42(2):26.

205. Thomson RG, Eccles MP, Steen IN, Greenaway J, Stobbart L, Murtagh MJ, et al. A patient decision aid to support shared decision-making on anti-thrombotic treatment of patients with atrial fibrillation: randomised controlled trial. Qual Saf Health Care. 2007;16(3):216-23.

206. Wijtvliet E, Tieleman RG, van Gelder IC, Pluymaekers N, Rienstra M, Folkeringa RJ, et al. Nurse-led vs. usual-care for atrial fibrillation. Eur Heart J. 2020;41(5):634-41.

207. Goette A, Schön N, Kirchhof P, Breithardt G, Fetsch T, Häusler KG, et al. Angiotensin II-antagonist in paroxysmal atrial fibrillation (ANTIPAF) trial. Circ Arrhythm Electrophysiol. 2012;5(1):43-51.

208. Jiang J, Gu X, Cheng CD, Li HX, Sun XL, Duan RY, et al. The Hospital-Community-Family-Based Telemedicine (HCFT-AF) Program for Integrative Management of Patients With Atrial Fibrillation: Pilot Feasibility Study. JMIR Mhealth Uhealth. 2020;8(10):e22137.

209. Shacham J, Birati EY, Malov N, Yanay Y, Steinberg DM, Tamari M, et al. Telemedicine for diagnosing and managing paroxysmal atrial fibrillation in outpatients. The phone in the pocket. Int J Cardiol. 2012;157(1):91-5.

210. Stegmann T, Koehler K, Wachter R, Moeller V, Zeynalova S, Koehler F, et al. Heart failure patients with atrial fibrillation benefit from remote patient management: insights from the TIM-HF2 trial. ESC Heart Fail. 2020;7(5):2516-26.

211. Stewart S, Ball J, Horowitz JD, Marwick TH, Mahadevan G, Wong C, et al. Standard versus atrial fibrillation-specific management strategy (SAFETY) to reduce recurrent admission and prolong survival: pragmatic, multicentre, randomised controlled trial. Lancet. 2015;385(9970):775-84.

212. Vinereanu D, Lopes RD, Bahit MC, Xavier D, Jiang J, Al-Khalidi HR, et al. A multifaceted intervention to improve treatment with oral anticoagulants in atrial fibrillation (IMPACT-AF): an international, cluster-randomised trial. Lancet. 2017;390(10104):1737-46.

213. Peleg M, Shahar Y, Quaglini S, Broens T, Budasu R, Fung N, et al. Assessment of a personalized and distributed patient guidance system. Int J Med Inform. 2017;101:108-30.

214. Pearsons A, Hanson CL, Gallagher R, O'Carroll RE, Khonsari S, Hanley J, et al. Atrial fibrillation self-management: a mobile telephone app scoping review and content analysis. Eur J Cardiovasc Nurs. 2020.

215. Guo Y, Lane DA, Wang L, Chen Y, Lip GYH, m AFAIITi. Mobile Health (mHealth) technology for improved screening, patient involvement and optimising integrated care in atrial fibrillation: The mAFA (mAF-App) II randomised trial. Int J Clin Pract. 2019;73(7):e13352.

216. Guo Y, Wang H, Zhang H, Liu T, Liang Z, Xia Y, et al. Mobile Photoplethysmographic Technology to Detect Atrial Fibrillation. J Am Coll Cardiol. 2019;74(19):2365-75.

217. Kotecha D, Chua WWL, Fabritz L, Hendriks J, Casadei B, Schotten U, et al. European Society of Cardiology smartphone and tablet applications for patients with atrial fibrillation and their health care providers. Europace. 2018;20(2):225-33.

218. Giebel GD, Gissel C. Accuracy of mHealth Devices for Atrial Fibrillation Screening: Systematic Review. JMIR Mhealth Uhealth. 2019;7(6):e13641.

219. Yang TY, Huang L, Malwade S, Hsu CY, Chen YC. Diagnostic Accuracy of Ambulatory Devices in Detecting Atrial Fibrillation: Systematic Review and Meta-analysis. JMIR Mhealth Uhealth. 2021;9(4):e26167.

220. Lane DA, McMahon N, Gibson J, Weldon JC, Farkowski MM, Lenarczyk R, et al. Mobile health applications for managing atrial fibrillation for healthcare professionals and patients: a systematic review. Europace. 2020.

221. Hindricks G, Potpara T, Dagres N, Arbelo E, Bax JJ, Blomstrom-Lundqvist C, et al. 2020 ESC Guidelines for the diagnosis and management of atrial fibrillation developed in collaboration with the European Association for Cardio-Thoracic Surgery (EACTS). Eur Heart J. 2021;42(5):373-498.

222. Eerikainen LM, Bonomi AG, Schipper F, Dekker LRC, Vullings R, de Morree HM, et al. Comparison between electrocardiogram- and photoplethysmogram-derived features for atrial fibrillation detection in free-living conditions. Physiol Meas. 2018;39(8):084001.

223. O'Sullivan JW, Grigg S, Crawford W, Turakhia MP, Perez M, Ingelsson E, et al. Accuracy of Smartphone Camera Applications for Detecting Atrial Fibrillation: A Systematic Review and Meta-analysis. JAMA Netw Open. 2020;3(4):e202064.

224. Hermans ANL, van der Velden RMJ, Gawalko M, Verhaert DVM, Desteghe L, Duncker D, et al. On-demand mobile health infrastructures to allow comprehensive remote atrial fibrillation and risk factor management through teleconsultation. Clin Cardiol. 2020.

225. Giudicessi JR, Schram M, Bos JM, Galloway CD, Shreibati JB, Johnson PW, et al. Artificial Intelligence-Enabled Assessment of the Heart Rate Corrected QT Interval Using a Mobile Electrocardiogram Device. Circulation. 2021.

226. Caillol T, Strik M, Ramirez FD, Abu-Alrub S, Marchand H, Buliard S, et al. Accuracy of a Smartwatch-Derived ECG for Diagnosing Bradyarrhythmias, Tachyarrhythmias, and Cardiac Ischemia. Circ Arrhythm Electrophysiol. 2021;14(1):e009260.

227. Strik M, Caillol T, Ramirez FD, Abu-Alrub S, Marchand H, Welte N, et al. Validating QT-Interval Measurement Using the Apple Watch ECG to Enable Remote Monitoring During the COVID-19 Pandemic. Circulation. 2020;142(4):416-8.

228. Drexler M, Elsner C, Gabelmann V, Gori T, Münzel T. Apple Watch detecting coronary ischaemia during chest pain episodes or an apple a day may keep myocardial infarction away. Eur Heart J. 2020;41(23):2224.

229. Wegner FK, Kochhauser S, Frommeyer G, Lange PS, Ellermann C, Leitz P, et al. Prospective blinded evaluation of smartphone-based ECG for differentiation of supraventricular tachycardia from inappropriate sinus tachycardia. Clin Res Cardiol. 2021;110(6):905-12.

230. Yasin OZ, Attia Z, Dillon JJ, DeSimone CV, Sapir Y, Dugan J, et al. Noninvasive blood potassium measurement using signal-processed, single-lead ecg acquired from a handheld smartphone. J Electrocardiol. 2017;50(5):620-5.

231. Pluymaekers N, Hermans ANL, van der Velden RMJ, Gawałko M, den Uijl DW, Buskes S, et al. Implementation of an on-demand app-based heart rate and rhythm monitoring infrastructure for the management of atrial fibrillation through teleconsultation: TeleCheck-AF. Europace. 2020.

232. Gawałko M, Elliott A, Kadhim K, Sanders P, Linz D. A call for a more objective and longitudinal reporting of lifestyle components in cardiovascular research. Int J Cardiol Heart Vasc. 2020;27:100506.
